# Downregulation of Elovl5 promotes breast cancer metastasis through a lipid-droplet accumulation-mediated induction of TGF-β receptors

**DOI:** 10.1038/s41419-022-05209-6

**Published:** 2022-09-02

**Authors:** Trinh-Le-Vi Kieu, Léa Pierre, Valentin Derangère, Sabrina Perrey, Caroline Truntzer, Antoine Jalil, Sébastien Causse, Emma Groetz, Adélie Dumont, Laura Guyard, Laurent Arnould, Jean-Paul Pais de Barros, Lionel Apetoh, Cédric Rébé, Emeric Limagne, Tony Jourdan, Laurent Demizieux, David Masson, Charles Thomas, François Ghiringhelli, Mickaël Rialland

**Affiliations:** 1 Institut National de la Santé et de la Recherche Médicale (INSERM) UMR, 1231 Dijon, France; 2 UFR Sciences de la Vie, Terre et Environnement, Université de Bourgogne Franche-Comté, Dijon, France; 3 LipSTIC LabEx, Dijon, France; 4 UFR des sciences de santé, Université de Bourgogne Franche-Comté, Dijon, France; 5 Centre Georges François Leclerc, Dijon, France; 6 Lipidomic Analytic Platform, Université de Bourgogne, Dijon, France

**Keywords:** Breast cancer, Cancer metabolism, Mechanisms of disease

## Abstract

Metastatic breast cancer cannot be cured, and alteration of fatty acid metabolism contributes to tumor progression and metastasis. Here, we were interested in the elongation of very long-chain fatty acids protein 5 (Elovl5) in breast cancer. We observed that breast cancer tumors had a lower expression of Elovl5 than normal breast tissues. Furthermore, low expression of Elovl5 is associated with a worse prognosis in ER^+^ breast cancer patients. In accordance with this finding, decrease of Elovl5 expression was more pronounced in ER^+^ breast tumors from patients with metastases in lymph nodes. Although downregulation of Elovl5 expression limited breast cancer cell proliferation and cancer progression, suppression of Elovl5 promoted EMT, cell invasion and lung metastases in murine breast cancer models. The loss of Elovl5 expression induced upregulation of TGF-β receptors mediated by a lipid-droplet accumulation-dependent Smad2 acetylation. As expected, inhibition of TGF-β receptors restored proliferation and dampened invasion in low Elovl5 expressing cancer cells. Interestingly, the abolition of lipid-droplet formation by inhibition of diacylglycerol acyltransferase activity reversed induction of TGF-β receptors, cell invasion, and lung metastasis triggered by Elovl5 knockdown. Altogether, we showed that Elovl5 is involved in metastasis through lipid droplets-regulated TGF-β receptor expression and is a predictive biomarker of metastatic ER^+^ breast cancer.

## Introduction

Breast cancer is the most frequent cancer in women with 2.26 million of new cases and the leading cause of cancer death in women with almost 685,000 deaths worldwide in 2020 [1]. The incidence and mortality of breast cancer steadily increase in the world with geographical and socioeconomic inequalities [2, 3]. Breast cancer is an heterogeneous disease with different subtypes defined by the following molecular classification: Luminal A and B (estrogen receptor-positive ER^+^), HER-2 positive (HER-2^+^ and ER^−^), basal-like (triple-negative breast cancer TNBC; ER^−^, PR^−^, and HER-2^−^) breast cancers [4–6]. A non-metastatic breast cancer has a good prognosis and the 5-year relative survival rate is ~90% in women [7–9]. On the other hand, the 5-year relative survival rate decreases to less than 30% for metastatic breast cancer with a median overall survival of ~3 years [8, 10]. Metastasis involves a multistep process resulting in the dissemination of cancer cells from the primary tumor to secondary sites [11]. The epithelial-to-mesenchymal transition (EMT) might be a critical mechanism for metastasis and is characterized by downregulation of epithelial marker expression (i.e., E-cadherin or occludin) and upregulation of mesenchymal markers (i.e., vimentin or N-cadherin) [12].

Metabolic adaptation is a hallmark of cancers, and a reprogramming of fatty acid (FA) metabolism is observed in many cancer cells. Breast cancer is associated with quantitative and qualitative changes in FA composition [13]. Abolition of lipogenesis through inhibition of lipogenic enzyme activity decreases cancer cell proliferation and metastatic processes [13–16]. Exogenous fatty acids also contribute to the proliferation of breast cancer cells and the development of metastases [17, 18]. Modifications of cellular FA by desaturation and elongation are common in cancer cells, and alterations in the expression of these involved enzymes is described in breast cancer [13, 19]. Seven very long-chain fatty acid elongases (Elovl1–7) have been identified in the mouse, rat, and human genomes. These enzymes determine the rate of overall fatty acid elongation with substrate selectivity depending on carbon chain length and unsaturation degree; Elovl5 preferentially elongates C18 and C20 unsaturated FA [20].

In this study, we were interested in the role of Elovl5 in breast cancer progression. We developed different murine breast cancer models and showed that Elovl5 controlled tumor growth and metastasis process through lipid-droplet-mediated regulation of TGF-β receptor expression.

## Results

### Elovl5 is downregulated in breast cancer, and a low expression of Elovl5 is associated with poor clinical outcome

**Fig. 1:**
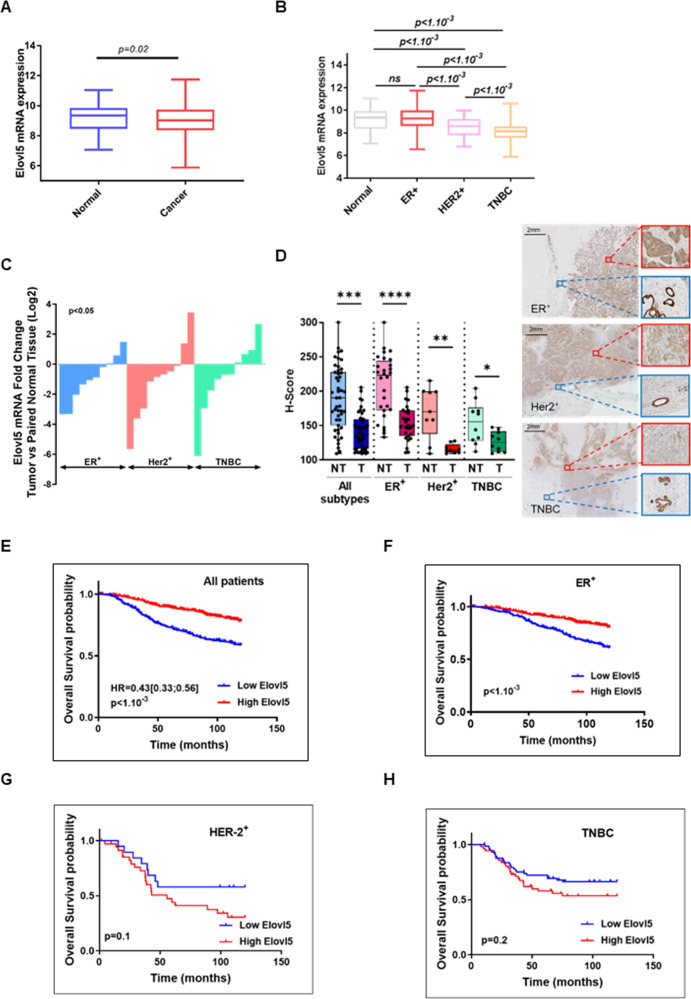
Expression of Elovl5 in women breast cancer patients. **A** Comparison of Elovl5 mRNA expression in normal (*n* = 144) and breast cancer (*n* = 957) tissues in the METABRIC cohort. The p value obtained using a Wilcoxon rank test is indicated. **B** Expression of Elovl5 mRNA in breast cancer subtypes (normal *n* = 144, ER^+^ *n* = 712, Her2^+^ *n* = 55, TNBC = 129) using METABRIC dataset. The *P* value was calculated using a Wilcoxon rank test. **C** Elovl5 mRNA expression in tumor sections relative to paired normal tissues are reported as Log2 of fold change. The *P* value was calculated using a Wilcoxon matched paired-rank test. **D** Box and whisker plots showing H-score of Elovl5 IHC staining in non-tumoral adjacent (NT) and tumoral (T) breast tissues from patients with different breast cancer subtypes. Median values are indicated with the horizontal line. The whiskers show the lowest and highest values. *P* < 0.05, **P < 0.01, ***P < 0.001, and ****P < 0.0001 and non-significant (ns) were obtained using a Wilcoxon rank test. Representative images are shown for each subtype with breast tumor tissues in red and normal adjacent tissues in blue. **E**–**H** Kaplan–Meyer curves for overall survival analysis in the METABRIC cohorts according to low (blue line) and high (red line) expression of Elovl5 mRNA. *P* value was calculated using the log-rank Mantel–Cox test.

The analysis of Elovl5 mRNA levels in the METABRIC dataset showed that the expression of Elovl5 mRNA is downregulated in breast cancer tissues (*n* = 957 samples) compared to non-matched normal breast tissues (*n* = 144 samples) (Fig. 1A). We also found that Elovl5 mRNA was differently expressed between the subtypes of breast cancer. Indeed, METABRIC dataset showed that ER^+^ breast cancer expressed higher Elovl5 mRNA levels than Her2^+^ and TNBC (Fig. 1B). Then, we analyzed Elovl5 mRNA expression in samples from 30 women with breast cancer for which we had breast tumor tissues and paired normal breast tissues (Supplementary Table S1). Overall, the analysis showed that the content of Elovl5 mRNA was significantly reduced in breast cancer tissues compared to matched normal tissues (Fig. 1C). We confirmed with a H-score for the IHC Elovl5 staining that the average expression of Elovl5 was downregulated in breast cancer (T) tissue compared to adjacent normal (NT) breast tissue and was higher in ER^+^ breast cancer samples than in ER^−^ breast cancer tissues (Her2^+^ and TNBC) (Fig. 1D and Supplementary Table S2). From the METABRIC dataset, we found that a lower expression of Elovl5 mRNA is associated with a shorter overall survival (OS) time for breast cancer patients without distinction of subtypes (Fig. 1E). In patients with ER^+^ cancer, low Elovl5 mRNA expression was associated with a worse OS prognosis compared to patients with high Elovl5 expression (Fig. 1F). No such association was observed for Her2^+^ and TNBC cancer patients (Fig. 1G, H).

### Silencing of Elovl5 expression inhibits breast cancer cell proliferation and tumor growth

**Fig. 2:**
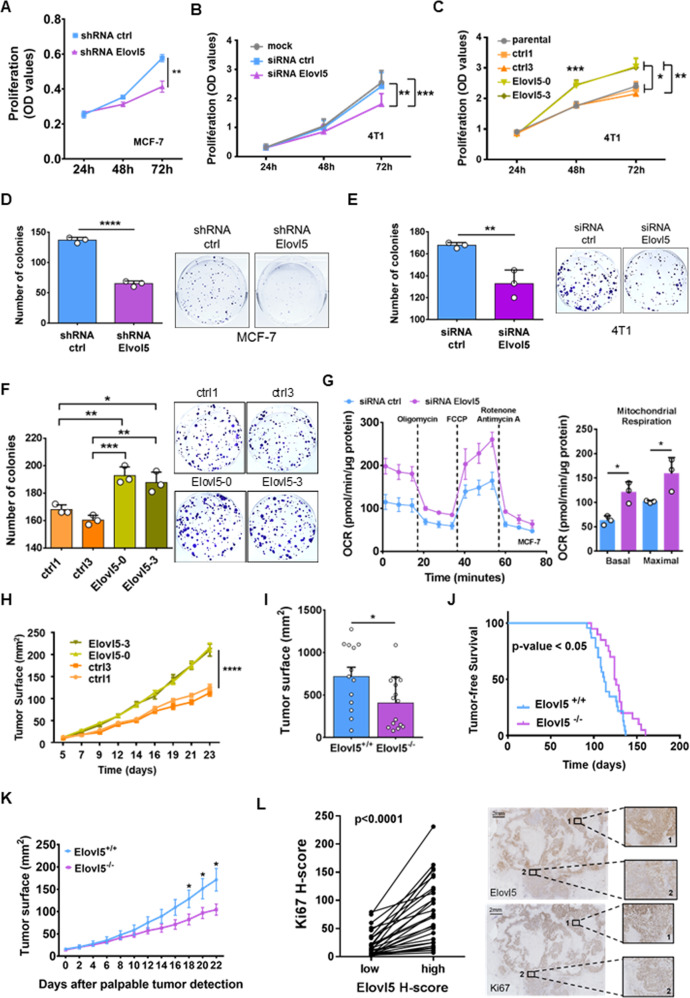
Cell proliferation and breast tumor growth under the control of Elovl5 expression. **A**–**C** Proliferation of Elovl5-depleted (**A**, **B**) and Elovl5-overexpressing cancer cells (**C**) evaluated by crystal violet staining. Error bars represent the mean ± SD of independent experiments (*n* = 3 (**A**), *n* = 4 (**B**) and *n* = 3 (**C**)) with *P < 0.05, **P < 0.01 and ***P < 0.001 using a one-way ANOVA analysis with Tukey test (**B**, **C**) and a Student’s *t* test at each time point for (**A**). **D**–**F** Colony-formation assay analyzed by crystal violet staining. Error bars represent the mean ± SD of three independent experiments with *P < 0.05, **P < 0.01, ***P < 0.001 and ****P < 0.0001 using a Student’s *t* test (**D**, **E**) or one-way ANOVA analysis with Tukey test (**F**). **G** Oxygen consumption rate measured with Seahorse XFe96 analyzer in Elovl5-depleted MCF-7 cells. Error bars represent the mean ± SD with *P < 0.05 according to Student’s *t* test. **H** Tumor growth of Elovl5-overexpressing 4T1 (Elovl5-0 and Elovl5-3) and control 4T1 (ctrl1 and ctrl3) cells injected in the mammary fat pad of female Balb-c mice (*n* = 10 per group). Statistical significance ****P < 0.0001 was calculated for Elovl5 and ctrl groups on day 23 using a one-way ANOVA with Tukey’s multiple comparison test. **I** Aggregated tumor surface analysis in MMTV-PyMT;Elovl5^+/+^ (Elovl5^+/+^) and MMTV-PyMT;Elovl5^–/–^ (Elovl5^–/–^) 6-month-old mice. Dots indicate the values of the cumulative surface of mammary tumor lesions for each individual mouse. Histograms and error bars indicate the mean ± SEM with *P < 0.05 according to a Mann–Whitney test. **J** Kaplan-Meier curves of tumor-free survival percentage in PyMT-Elovl5^+/+^ (*n* = 23) and PyMT-Elovl5^−/−^ (*n* = 20) mice. A log-rank (Mantel–Cox) statistical test was used. **K** Tumor growth analysis in MMTV-PyMT;Elovl5^+/+^ (Elovl5^+/+^, *n* = 13) and MMTV-PyMT;Elovl5^−/−^ (Elovl5^−/−^, *n* = 15) mice. Values used are the cumulative surface of tumors per mice. Curves and error bars represent the mean ± SEM with *P < 0.05 according to Student’s *t* test calculated for each time point. **L** Analysis of Ki67 H-score according to the lowest and highest values of Elovl5 H-score from tumor tissues of breast cancer patients. Each line links the lowest and highest value of Elovl5 H-score from the same patient. The *P* value was obtained using a paired Student’s *t* test. Representative images of Elovl5 and Ki67 IHC with the corresponding high (1) and low (2) H-score region.

To precise the role of Elovl5 in breast cancer cell proliferation, we used mammary cell lines with different basal Elovl5 expression levels, in which we further downregulated or overexpressed Elovl5 expression (Supplementary Fig. S1A–G). Lipidomic analysis of mono- and polyunsaturated FA composition by Gas Chromatography-Mass Spectrometry (GC-MS) between Elovl5-silenced breast cancer cells and their respective control breast cancer cells resulted in the accumulation of C16:1 n-7 and C18:3 n-6 FA (Supplementary Fig. S1H–J) whereas C16:1 n-7 and C18:3 n-6 content decreased in 4T1 cells stably overexpressing Elovl5 (Supplementary Fig. S1K). We then showed that a reduction of Elovl5 expression decreased the cell proliferation analyzed by crystal violet staining (Fig. 2A, B and Supplementary Fig. S2A). Conversely, a stable overexpression of Elovl5 in 4T1 cells (Elovl5-0 and Elovl5-3) resulted in an increase of in vitro proliferation compared to control 4T1 (ctrl1 and ctrl3) (Fig. 2C). The colony-formation assays showed that Elovl5 silencing (Fig. 2D, E) decreased the number of MCF-7 or 4T1 colonies whereas Elovl5 overexpression increased the formation of 4T1 colonies (Fig. 2F). The loss of Elovl5 expression did not trigger cell death (Supplementary Fig. S2B) but induced a cell cycle arrest in G0-G1 phase (Supplementary Fig. S2C) supporting the inhibition of proliferation in Elovl5-depleted breast cancer cells. A previous publication reported a mitochondrial dysfunction of Elovl5-silenced prostate cancer cells affecting their proliferation [21]. Unexpectedly, the transient or stable depletion of Elovl5 expression in the MCF-7 breast cancer cells led to the increase of the basal and maximal mitochondrial oxygen consumption (Fig. 2G and Supplementary Fig. S2D). Next, we assessed the effect of Elovl5 overexpression in tumor growth by grafting 4T1 cells stably overexpressing Elovl5 or control 4T1 cells in the fourth mammary fat pad of female Balb-c mice. Our results showed that Elovl5 expression increased the 4T1 tumor growth (Fig. 2H). We also used the MMTV-PyMT mammary cancer mouse model, which mimics human luminal B breast cancer [22]. To evaluate the role of Elovl5 invalidation in breast cancer progression, we crossed *Elovl5* full knockout female C57BL/6 mice with MMTV-PyMT male C57BL/6 mice. After the confirmation of Elovl5 gene invalidation (Supplementary Fig. S2E, F), we found as expected that mammary tumor tissues from MMTV-PyMT;Elovl5^−/−^ (hereafter termed Elovl5^−/−^) mice had a significantly increased in the percentage of C18:3 n-6 FA compared to mammary tumor tissues of MMTV-PyMT;Elovl5^+/+^ (hereafter termed Elovl5^+/+^) (Supplementary Fig. S2G). We then monitored tumor growth until the sacrifice of the mice at 180 days of age; all female mice developed at least one mammary tumor at this time point. The surface of the aggregated tumor lesions at 180 days was lower in Elovl5^−/−^ than in Elovl5^+/+^ mice (Fig. 2I). However, we observed a slight delay in tumor onset in Elovl5^−/−^ compared to Elovl5^+/+^ mice which could explain the difference in tumor surface (Fig. 2J). Therefore, we determined the tumor growth rate in the 22 days starting from the detection of the first tumor. In these 22 days, the mean aggregated tumor surface increased 11.6-fold in Elovl5^+/+^ mice and only 7.4-fold in Elovl5^−/−^ mice (Fig. 2K and Supplementary Fig. S2H). To evaluate a clinical relevance of Elovl5 expression in human breast tumor growth, we determined the breast tumoral zone with the lowest and with the highest Elovl5 H-score for each patient, and we found that cancer cell proliferation analyzed by a Ki67 H-score is positively correlated with Elovl5 H-score in the corresponding breast tumor lesion (Fig. 2L). Altogether these data show that Elovl5 controlled proliferation and tumor growth in breast cancer.

### Loss of Elovl5 promotes the development of lung metastases

**Fig. 3:**
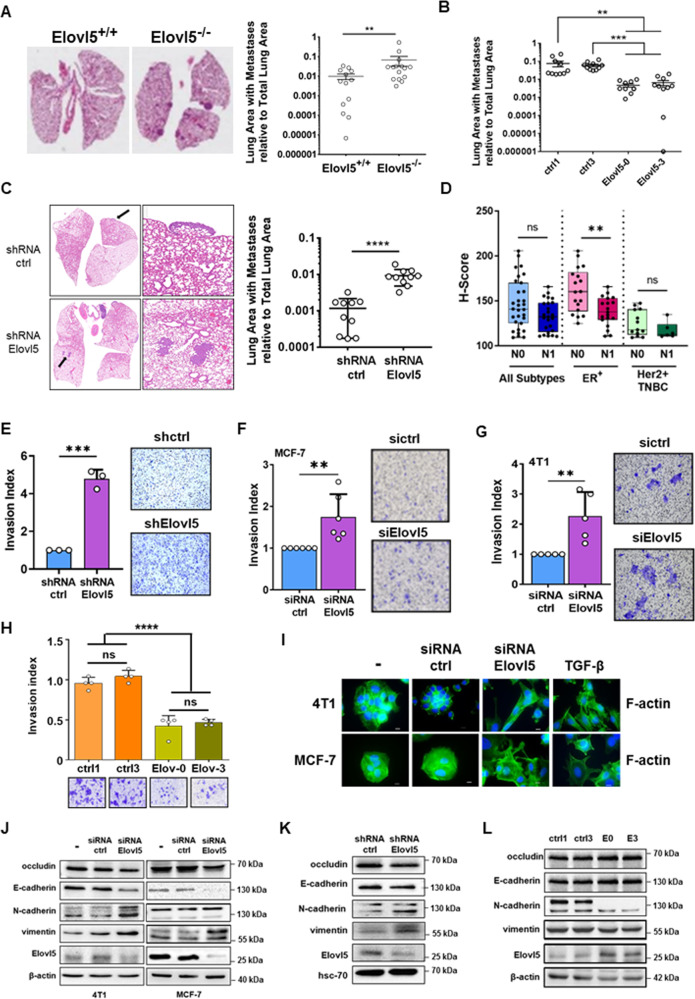
Elovl5 regulates metastasis, cell invasion, and expression of EMT markers. **A**–**C** QuPath analysis for quantification of lung surface with metastases in 6-month-old female MMTV-PyMT;Elovl5^+/+^ (Elovl5^+/+^) and MMTV-PyMT;Elovl5^−/−^ (Elovl5^−/−^) mice (**A**), in lungs of female Balb-c mice with fat pad injection of Elovl5-overexpressing (Elovl5-0 and Elovl5-3) or control (ctrl1 and ctrl3) 4T1 cells (**B**), in lungs of female NMRI-nude mice with tail vein injection of shRNA Elovl5 or shRNA control (ctrl) MCF-7 (**C**). Representative images of H&E staining are shown. Data represent the mean ± SEM. **D** Box and whisker plots showing H-score for Elovl5 IHC staining in breast cancer tissues of different subtypes (ER^+^, Her2^+^, and TNBC) from women patients with lymph node invasion (N1) or without invasion (N0). Median values are indicated with the horizontal line. The whiskers show the lowest and highest values. **E**, **F** Fold change of cell invasion for MCF-7 cells with stable (shRNA Elovl5) or transient (siRNA Elovl5) silencing of Elovl5 expression compared to respective control (shRNA ctrl or siRNA ctrl) MCF-7 cells. Invasive MCF-7 cells were stained with crystal violet for quantification. Histograms and error bars represent the mean ± SD. **G** Cell invasion analysis for Elovl5-silenced 4T1 cells (siRNA Elovl5) and non-targeting siRNA (siRNA ctrl). Histograms and error bars represent the mean ± SD. **H** Cell invasion index for Elovl5-overexpressing (Elov-0 and Elov-3) or control (ctrl1 and ctrl3) 4T1 cells. Data are represented as mean ± SD. **I** Fluorescence staining of actin fibers with FITC-phalloidin in 4T1 and MCF-7 cells treated for 48 h with Elovl5 and non-targeting (ctrl) siRNA. Nuclei were counterstained with Hoechst. TGF-β treatment is used as a positive control for EMT. **J**–**L** Analysis of EMT markers by western blotting in Elovl5-silenced MCF-7 and Elovl5-overexpressing 4T1 cells. Images are representative of at least two independent experiments. **P < 0.01, ***P < 0.001, ****P < 0.0001, and non-significant (ns) were calculated using Mann–Whitney test (**A**, **C**, **D**), Student’s *t* test (**E**, **F**, **G**), Kuskall–Wallis analysis with Dunn’s test (**B**) and one-way ANOVA analysis with Tukey’s test (**H**).

A weak expression of Elovl5 is associated with a worse prognosis in breast cancer. However, the breast cancer aggressiveness is not dependent on the cancer cell proliferation since low expression of Elovl5 inhibited proliferation (Fig. 2). Therefore, we investigated the role of Elovl5 in metastasis which remains the main risk of death in breast cancer patients [23]. We observed that the Elovl5^−/−^ mice developed more metastases on their lung surface than the Elovl5^+/+^ mice (Supplementary Fig. S3A, B). The quantification of metastases area in H&E-stained sections from lungs using QuPath analysis confirmed that the area occupied by metastases relative to the total lung area was greater in Elovl5^−/−^ than in Elovl5^+/+^ mice (Fig. 3A). Moreover, mice transplanted with Elovl5-overexpressing 4T1 cells in their mammary fat pad showed lower metastases counts on their lung surface and less lung area was invaded by metastases in comparison to control 4T1 tumor-bearing mice (Fig. 3B and Supplementary Fig. S3C, D). To further investigate the role of Elovl5 in the metastatic process, we injected MCF-7 or 4T1 cells with different Elovl5 expression levels in the tail vein, to partially mimic metastasis. As expected, we observed that stable downregulation of Elovl5 in MCF-7 (shRNA Elovl5) resulted in more metastases in the lungs of female NMRI-nude mice (Fig. 3C and Supplementary Fig. S3E). In contrast, tail vein injection of 4T1 cells stably overexpressing Elovl5 in female Balb-c mice led to a decrease in the number of metastases and lung surface with metastases in comparison to control 4T1 cells (Supplementary Fig. S3F, G). To confirm the association of Elovl5 expression and lymph node metastasis in breast cancer, we conducted an IHC analysis of patients with or without lymph node invasion (Table S3). The Elovl5 H-score defined with QuPath analysis on breast cancer tissues was significantly higher in ER^+^ patients without metastases in lymph nodes (N0) than with metastases in lymph nodes (N1). No differences in the Elovl5 H-score were observed between N0 and N1 status in patients with Her2^+^ or TNBC cancers (Fig. 3D and Supplementary Fig. S3H). Moreover, we confirmed that the Elovl5 H-score is higher in ER^+^ patients (both N0 and N1) than in Her2^+^ or TNBC patients (Fig. 3E). These results demonstrate that a decrease in Elovl5 expression correlates to lymph node invasion in ER^+^ breast cancers and promotes the formation of lung metastases in mouse breast cancer models.

### Downregulation of Elovl5 increases the invasiveness and expression of EMT markers

The metastatic process at the cellular level is associated with the acquisition of invasive properties and a mesenchymal phenotype [12]. Stable or transient Elovl5 downregulation significantly promoted invasiveness in low invasive MCF-7 cells (Fig. 3E, F). In 4T1 cells with high basal invasive properties, knockdown of Elovl5 expression (siRNA Elovl5) also increased cell invasion compared to control (siRNA ctrl) 4T1 cells (Fig. 3G), whereas Elovl5 overexpression (Elov-0 and Elov-3) limited invasiveness of 4T1 cells in comparison to control (ctrl1 and ctrl3) 4T1 cells (Fig. 3H). Changes in cell morphology through actin cytoskeleton remodeling is critical for invasion. Therefore, we visualized actin fibers (F-actin) with FITC-conjugated phalloidin staining and found that suppression of Elovl5 in 4T1 and MCF-7 cells induced a mesenchymal-like morphology, like TGF-β used as a positive control (Fig. 3I). Then, we analyzed by western blotting the expression of EMT markers which showed a decrease in the expression of epithelial markers (E-cadherin and occludin) and an increased expression of mesenchymal markers (vimentin and N-cadherin) in Elovl5-silenced breast cancer cells (Fig. 3J, K). In contrast, overexpression of Elovl5 in 4T1 cells (E0 and E3) reduced the expression of mesenchymal markers (vimentin and N-cadherin) and slightly increased the expression of epithelial markers (E-cadherin and occludin) (Fig. 3L). These data highlight that repression of Elovl5 expression sustains invasiveness and EMT in breast cancer cells.

### Elovl5-regulated expression of TGF-β receptors controls cell proliferation and invasion

**Fig. 4:**
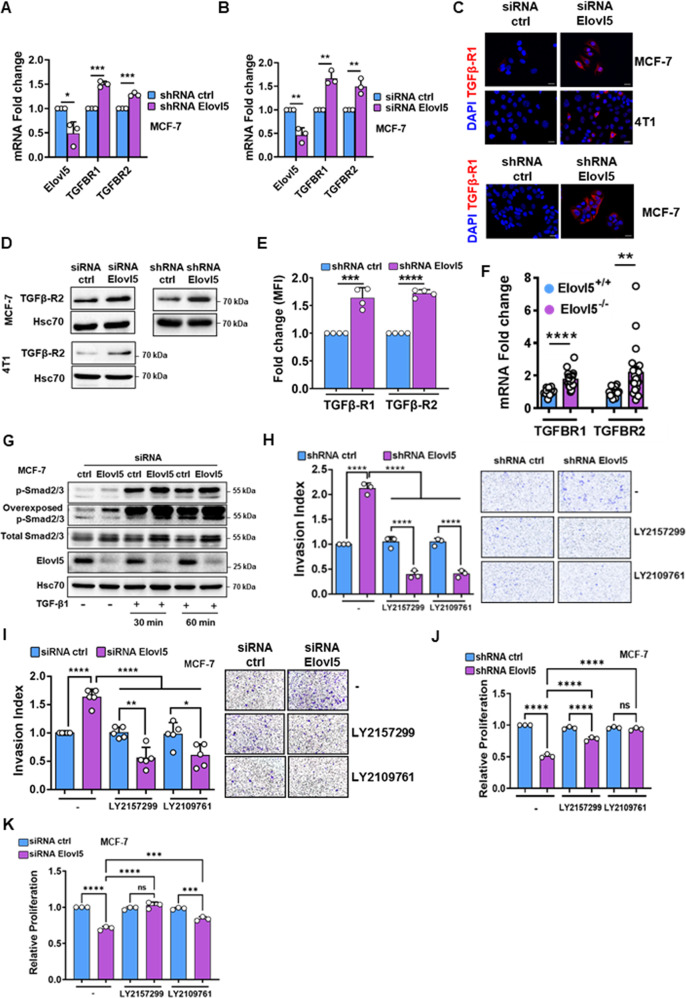
Elovl5 modulates the expression of TGF-β receptors. **A**, **B** Analysis of TGFBR1 and TGFBR2 mRNA expression at 24 h by RT-qPCR in Elovl5-silenced MCF-7 cells by shRNA (**A**) and siRNA (**B**) relative to control shRNA or siRNA-treated MCF-7 cells. Histograms and error bars represent the mean ± SD of three independent experiments. **C** Immunofluorescence staining of TGF-β receptor 1 (TGFβ-R1) in Elovl5-silenced MCF-7 and 4T1 cells compared to a control siRNA or shRNA at 48 h. Nuclei are stained with Hoechst. Images are representative of three independent experiments. **D** Analysis of TGF-β receptor 2 (TGFβ-R2) in control and Elovl5-silenced MCF-7 and 4T1 cells at 48 h by western blotting. Images are representative of three independent experiments. **E** Analysis of TGF-β receptor 1 and 2 expression at 48 h by flow cytometry in Elovl5-targeting shRNA and control shRNA-treated MCF-7 cells. Values are the fold change in the mean of fluorescence intensity (MFI) of shRNA Elovl5 cells relative to shRNA control cells. Histograms and error bars represent the mean ± SD of four independent experiments. **F** Analysis of TGF-β receptor 1 and 2 expression by RT-qPCR in tumors from 140 days-old MMTV-PyMT;Elovl5^+/+^ (Elovl5^+/+^; *n* = 11) and MMTV-PyMT;Elovl5^−/−^ (Elovl5^−/−^; *n* = 20) mice. Data are expressed as the mean ± SEM. **G** Analysis of Smad2/3 phosphorylation (p-Smad2/3) and total Smad2/3 expression in Elovl5-silenced MCF-7 cells treated with TGF-β1 (5 ng/ml) for the indicated times by western blotting. **H**, **I** Analysis of cell invasion for Elovl5-depleted MCF-7 cells using shRNA (**H**) or transient siRNA (**I**) against Elovl5 relative to MCF-7 cells treated with a control shRNA or siRNA (ctrl). Histograms and error bars represent the mean ± SD of three independent experiments. Representative images are shown. **J**, **K** Analysis of proliferation by crystal violet staining in Elovl5-depleted MCF-7 cells using an shRNA (**J**) or siRNA (**K**) against Elovl5 relative to MCF-7 cells treated with a control shRNA or siRNA (ctrl). Histograms and error bars are expressed as the mean ± SD of three independent experiments. *P < 0.05, **P < 0.01, ***P < 0.001, ****P < 0.0001 and non-significant (ns) were calculated using Student’s *t* test (**A**, **B**, **E**, **F**) and one-way ANOVA analysis with Tukey’s test (**H**, **I**, **J**, **K**).

The transforming growth factor-β (TGF-β) pathway is a major contributor of EMT and metastasis. Thus, we aimed to investigate the expression of TGF-β receptors in breast cancer cells upon modulation of Elovl5 expression. Silencing of Elovl5 expression in both MCF-7 and 4T1 cells induced the expression of TGFBR1 and TGFBR2 mRNA (Fig. 4A, B and Supplementary Fig. S4A). Conversely, the stable overexpression of Elovl5 in 4T1 cells (Elovl5-0 and Elovl5-3) decreased the expression of TGFBR1 and TGFBR2 mRNA (Supplementary Fig. S4B). We confirmed at the protein level that the expression of TGF-β receptor 1 (TGFβ-R1) by immunofluorescence staining (Fig. 4C) and expression of TGF-β receptor 2 (TGFβ-R2) by western blotting (Fig. 4D) were enhanced in Elovl5-depleted breast cancer cells. Furthermore, we demonstrated an increase in TGFβ-R1 and TGFβ-R2 expression at the plasma membrane by flow cytometry analysis of breast cancer cells with suppressed Elovl5 expression (Fig. 4E and Supplementary Fig. S4C) while stable overexpression of Elovl5 reduced their exposure at the plasma membrane (Supplementary Fig. S4D). Moreover, we were able to show an increase of TGFBR1 and TGFBR2 mRNA expression in breast tumors from Elovl5^−/−^ mice compared to tumors from Elovl5^+/+^ (Fig. 4F). Given that TGFβ-R1 and TGFβ-R2 expression were upregulated in Elovl5-silenced breast cancer cells, such cancer cells should present a higher activation of downstream signaling in response to TGF-β treatment. To evaluate the activation of the TGF-β pathway, we analyzed phosphorylation of smad2/3 (p-smad2/3) by western blotting. We showed that TGF-β1 treatment (5 ng/ml) for 30 or 60 min in Elovl5-silenced cancer cells induced a higher p-smad2/3 expression highlighting that the TGF-β pathway was more activatable (Fig. 4G and Supplementary Fig. S4E). It is noteworthy that there was an induction of p-smad2/3 with downregulation of Elovl5 in untreated conditions suggesting the presence of TGF-β in the extracellular microenvironment. We then evaluated the mRNA expression of the different TGF-β isoforms, and we found an increase in TGF-β3 transcripts in Elovl5-depleted MCF-7 and 4T1 cells (Supplementary Fig. S4F, G). To demonstrate the role of TGF-β receptors in Elovl5-dependent cell invasion and proliferation, we inhibited TGF-β receptor activity with pharmacological drugs. The treatment with LY2157299 and LY2109761 prevented the increase of Elovl5-depleted MCF-7 and 4T1 cell invasion compared to control cells (Fig. 4H, I and Supplementary Fig. S4H). Moreover, treatment with the inhibitors of TGF-β receptors (LY2157299 and LY2109761) alleviated the repression of proliferation induced by Elovl5 silencing in breast cancer cells (Fig. 4J, K and Supplementary Fig. S4I). Collectively, these data suggest regulation of TGF-β receptor expression by Elovl5.

### Suppression of Elovl5 drives DGAT1/2-dependent accumulation of lipid droplets

**Fig. 5:**
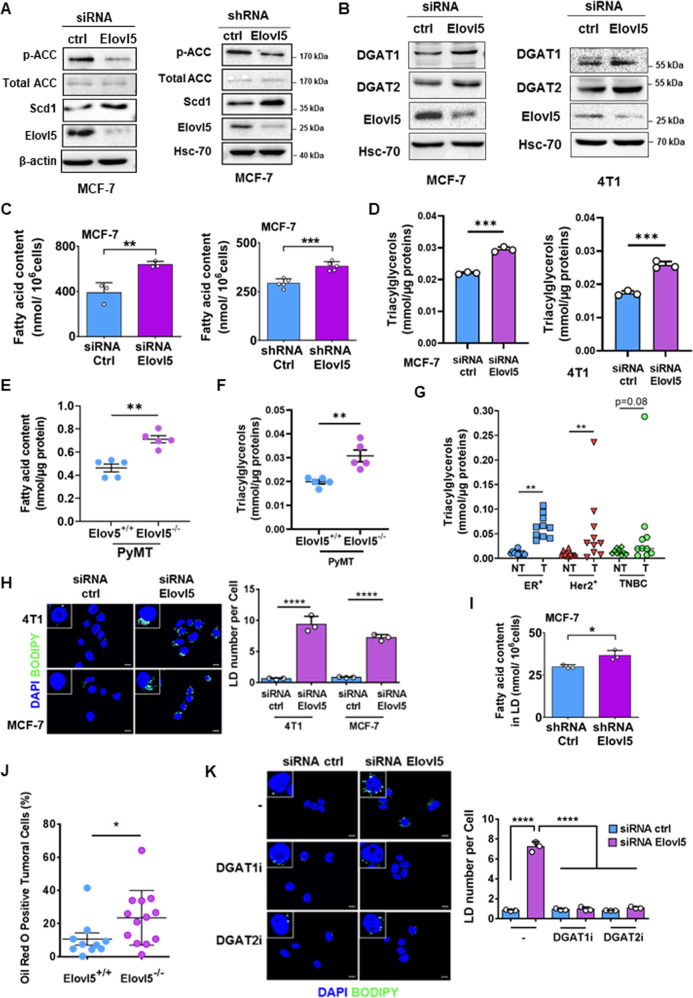
Elovl5 downregulation increases lipid-droplet abundance. **A** Expression of lipogenic enzymes analyzed by western blotting in Elovl5-silenced MCF-7 cells. **B** Expression of DGAT1 and DGAT2 enzymes analyzed by western blotting in MCF-7 and 4T1 cells transfected with a control or Elovl5-targeting siRNA. **C** Determination of total fatty acid content in stable or transient Elovl5-depleted MCF-7 cells. Data are expressed as the mean ± SD of three independent experiments. **P < 0.01 and ***P < 0.001 were determined by a Student’s *t* test. **D** Analysis of triacylglycerol content in Elovl5-silenced MCF-7 or 4T1 cells treated with siRNA targeting Elovl5 or a control siRNA. Data are expressed as the mean ± SD of three independent experiments. ***P < 0.001 was determined by a Student’s *t* test. **E**, **F** Determination of total fatty acid content by GC-MS (**E**) and triacylglycerol content by enzymatic assay (**F**) in tumors from MMTV-PyMT;Elovl5^+/+^ (Elovl5^+/+^; *n* = 5) and MMTV-PyMT;Elovl5^−/−^ (Elovl5^−/−^; *n* = 5). Data are expressed as the mean ± SEM. **P < 0.01 was determined by Student’s *t* test. **G** Analysis of triacylglycerol content in patient breast cancer subtypes (T) and paired non-tumoral breast (NT) tissues. **P < 0.01 was determined according to the Wilcoxon test. **H** Lipid-droplet staining with Bodipy 493/503 in 4T1 and MCF-7 cells transfected with siRNA targeting Elovl5 or a control siRNA (ctrl). Histograms are average of the lipid-droplet number per cell. Data are expressed as the mean ± SD of three independent experiments. ****P < 0.0001 was determined by a Student’s *t* test. Scale bar: 20 µm. **I** Amount of total fatty acids in lipid droplets purified from control MCF-7 cells or with stable Elovl5 depletion. Data are expressed as the mean ± SD of three independent experiments. *P < 0.05 were determined by Student’s *t* test. **J** Oil Red O lipid-droplet staining in mammary tumors of MMTV-PyMT;Elovl5^+/+^ (Elovl5^+/+^; *n* = 10) and MMTV-PyMT;Elovl5^−/−^ (Elovl5^−/−^; *n* = 13) mice. Data represent the percentage of positive-stained tumor cells with *P < 0.05 determined by a Mann–Whitney test. **K** Lipid-droplet quantification by fluorescence microscopy after Bodipy490/503 staining in Elovl5-depleted MCF-7 in the presence or absence of DGAT inhibitors. Histograms show the average number of lipid droplets per cell and error bars represent the mean ± SD of three independent experiments with ****P < 0.0001 according to one-way ANOVA analysis with Tukey’s test. Scale bar: 20 µm.

In order to evaluate the modification of lipid metabolism under the Elovl5 regulation, we analyzed the expression of lipogenic and triacylglycerols (TAG) synthesis enzymes by western blotting. The Elovl5 depletion in MCF-7 cells induced a lipogenic program characterized by a decrease of the inhibitory phosphorylation of ACC and an increase of Scd1 expression (Fig. 5A). Furthermore, we evaluated the expression of Diacylglycerol Acyltransferases (DGAT) 1 and 2 which participate in TAG synthesis, and found an increase in DGAT1 and DGAT2 expression in Elovl5-silenced cancer cells (Fig. 5B and Supplementary Fig. S5A). We then determined the total FA content by GC-MS and TAG content by enzymatic assay in breast cancer cells. We observed that transient depletion of Elovl5 in breast cancer cells using a siRNA for 48 h or its stable depletion by a shRNA in MCF-7 cells led to an intracellular accumulation of total FA (Fig. 5C) and TAG (Fig. 5D). Mammary tumor tissues from Elovl5^−/−^ mice also harbored higher FA and TAG content compared to mammary tumor tissues from Elovl5^+/+^ mice (Fig. 5E, F). In addition, we analyzed TAG content in breast tumor tissues in which Elovl5 expression was lower compared to their paired non-tumoral counterparts from patients with different cancer subtypes (Fig. 5G). The TAG content was significantly higher in breast tumor tissues than in paired non-tumoral tissues from patients with ER^+^ and Her2^+^ breast cancer; a non-significant increase in TAG concentration was also observed in tumor tissues of the TNBC subtype compared to paired normal tissues (Fig. 5G). Moreover, the average TAG concentration was different between subtypes with the highest concentration in the ER^+^ subtype (Fig. 5G). TAG are a major neutral lipid constituent of lipid droplets (LD), storing the excess of FA, and the final step of their synthesis depends on DGAT1/2 activity [24]. We showed that the breast cancer cells (MCF-7 and 4T1) with depletion of Elovl5 expression increased their content of intracellular LD (green dots in the cells) detected by fluorescence microscopy using Bodipy 493/503 or Nile red staining (Fig. 5H and Supplementary Fig. S5B–D). In accordance, the amount of total fatty acids in lipid droplets isolated from Elovl5-silenced MCF-7 cells is increased compared to control MCF-7 cells (Fig. 5I). The total fatty acids in LD from control MCF-7 cells contained 81.6% of SFA, 16.2% of MUFA and 2.2% of PUFA whereas 95.7% of SFA, 3.4% of MUFA and 0.9% of PUFA are found in LD from Elovl5-silenced MCF-7 cells (Supplementary Table S4 and Supplementary Fig. S5E–G). On the contrary, the depletion of Elovl5 expression did not drastically change the cellular SFA (62.8% vs 62.1%), MUFA (31.4% vs 29.4%; *P* < 0.05) and PUFA (7.1% vs 7.7%; *P* < 0.05) proportion compared to the control MCF-7 cells (Supplementary Table S4). Interestingly, the cellular content in the C24:5 and C24:6-C30:6 fatty acids increased in Elovl5-depleted MCF-7 cells (Supplementary Fig. S5G). Among the ≥C24 PUFA, their percentages decreased in LD from Elovl5-silenced MCF-7 cells except for the C26:6 (Supplementary Fig. S5G). The effect of Elovl5 invalidation on LD content in mammary tumors was investigated, and the analysis showed more Oil Red O stained-positive cancer cells in mammary tumors of Elovl5^-/-^ mice compared to the Elovl5^−/−^ mice (Fig. 5J and Supplementary Fig. S5H). Finally, to explore the role of DGAT isoforms in LD formation in an Elovl5-dependent manner, we inhibited DGAT1 and DGAT2 activity with pharmacological agents A922500 (DGAT1i) and PF-06424439 (DGAT2i) respectively and observed a decrease in LD accumulation in Elovl5-depleted breast cancer cells treated with the DGAT inhibitors analyzed by fluorescence microscopy or flow cytometry (Fig. 5K and Supplementary Fig. S5I–N). Altogether, the data highlight the role of Elovl5 in DGAT1/2-dependent LD formation.

### Elovl5 extinction-induced lipid droplets regulate the expression of TGF-β receptors through Smad2 acetylation

**Fig. 6:**
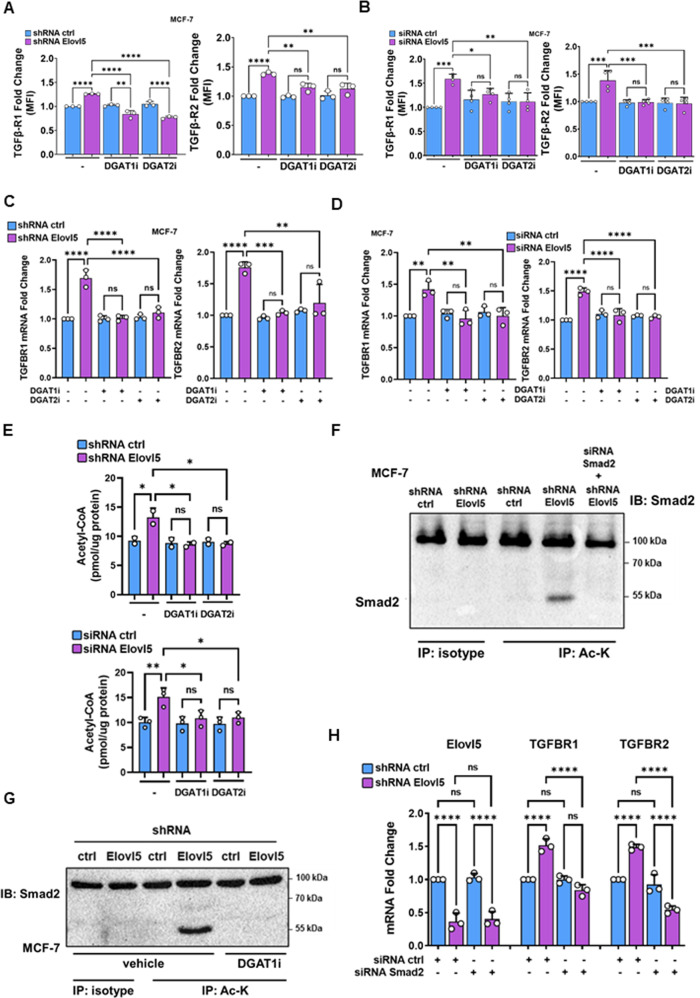
Lipid droplets mediate the dependent expression of TGF-β receptors in Elovl5-silenced breast cancer cells. **A**, **B** Analysis of TGFβ-1 and 2 receptor expression in MCF-7 cells by flow cytometry. MCF-7 cells with stable shRNA or siRNA silencing of Elovl5 expression were treated for 48 h with DGAT1 and 2 inhibitors and collected for anti-TGFβ-R1 and TGFβ-R2 staining. Values are fold change of mean fluorescence intensities (MFI) relative to MCF-7 cells treated with vehicle (DMSO) and control shRNA or siRNA (ctrl). Histograms and error bars are expressed as the mean ± SD of at least three independent experiments. **C**, **D** Analysis of TGFBR1 and TGFBR2 mRNA expression in MCF-7 cells. MCF-7 cells with stable shRNA or transient siRNA silencing of Elovl5 expression were treated for 48 h with DGAT1 and 2 inhibitors. Values used are fold changes of Elovl5 mRNA expression relative to MCF-7 cells treated with vehicle (DMSO) and control shRNA or siRNA (ctrl). Histograms and error bars are expressed as the mean ± SD of three independent experiments. **E** Quantification of acetyl-CoA in Elovl5-silenced MCF-7 cells using an siRNA or shRNA and treated with DGAT1 and DGAT2 inhibitors. Histograms and error bars represent the mean ± SD of five independent experiments. **F** Level of Smad2 acetylation in low Elovl5 expressing (shRNA Elovl5) and control (shRNA ctrl) MCF-7 cells. A sample of siRNA Smad2-treated MCF-7 cells was used as control of Smad2 silencing. Images are representative of two independent experiments. **G** Analysis of Smad2 acetylation in shRNA control and shRNA Elovl5 MCF-7 treated with DGAT1 inhibitor. Images are representative of two independent experiments. **H** Analysis of TGFBR1 and TGFBR2 mRNA expression at 48 h by RT-qPCR in Elovl5-targeting shRNA and control shRNA MCF-7 cells treated by siRNA ctrl or against smad2. Values are the fold change relative to shRNA control MCF-7 cells transfected with siRNA control. Histograms and error bars represent the mean ± SD of three independent experiments. *P < 0.05, **P < 0.01, ***P < 0.001, ****P < 0.0001 and non-significant (ns) were determined by one-way ANOVA analysis with Tukey’s test (**A**, **B**, **C**, **D**, **E**, **H**).

To further investigate the functions of LD in Elovl5-dependent effects, we first analyzed levels of TGF-β receptors at the plasma membrane of breast cancer cells lacking Elovl5 expression and exposed to DGAT inhibitors. Treatments with inhibitors of DGAT1 (A922500, DGAT1i) and DGAT2 (PF-06424439, DGAT2i) activity for 48 h were able to counteract the increase of plasma membrane TGFβ-R1 and TGFβ-R2 levels in Elovl5-depleted breast cancer cells (Fig. 6A, B and Supplementary Fig. S6A). Similarly, preventing LD accumulation by A922500 and PF-06424439 for 48 h abrogated the induction of TGFBR1 and TGFBR2 mRNA expression in MCF-7 and 4T1 cells without Elovl5 expression (Fig. 6C, D and Supplementary Fig. S6B). LD are a reservoir of substrates for fatty acid oxidation contributing to acetyl-CoA production [24]. Thus, we showed that silencing of Elovl5 expression in MCF-7 cells led to increase of acetyl-CoA levels which is reversed by inhibition of DGAT1 and DGAT2 activity (Fig. 6E). Moreover, acetyl-CoA is a source of acetyl for post-translational Smad2 acetylation which activates its signaling [15]. Therefore, we analyzed Smad2 acetylation in MCF-7 cells with Elovl5 suppression (shRNA Elovl5) and found an increase of acetylated Smad2 content compared to control (shRNA control) MCF-7 cells (Fig. 6F). Furthermore, we showed that DGAT1 inhibition suppressed Smad2 acetylation induced by Elovl5 downregulation (Fig. 6G). We postulated that Smad2 regulates the expression of TGF-β receptors due to its acetylation. Thus, the inhibition of Smad2 expression using siRNA transfection (Supplementary Fig. S6C) in Elovl5-silenced MCF-7 cells counteracted induction of TGF-β receptor expression mRNA (Fig. 6H) and protein (Supplementary Fig. S6D). Overall, the data support the role of Smad2 in the regulation of TGF-β receptor expression through its acetylation.

### Elovl5 loss-dependent lipid-droplet accumulation promotes EMT, cell invasion, and lung metastasis

**Fig. 7:**
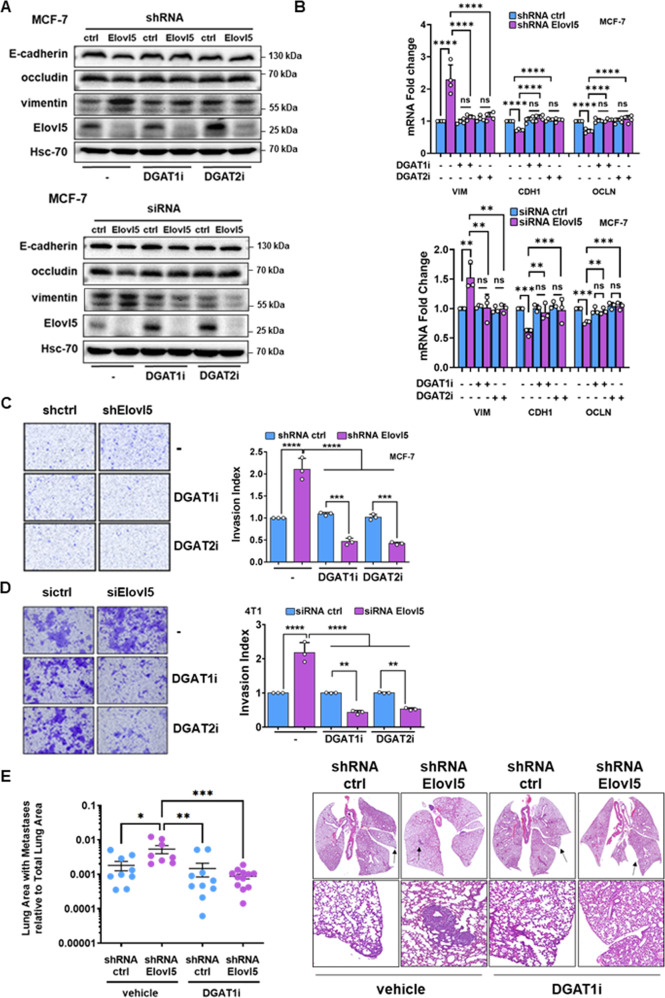
Elovl5 loss-induced lipid droplets enhance EMT, cell invasion, and lung metastasis. **A** Expression of EMT markers analyzed by western blotting in Elovl5-depleted MCF-7 cells using an shRNA or siRNA treated with vehicle (DMSO) or DGAT inhibitors (DGAT1i and DGAT2i) for 48 h. Images are representative of two independent experiments. **B** Expression of EMT marker mRNA (VIM, vimentin; CDH1, cadherin-1, and OCLN, occludin) analyzed by RT-qPCR in Elovl5-silenced MCF-7 cells treated with vehicle (DMSO) or DGAT inhibitors (DGAT1i and DGAT2i) for 48 h. Data are expressed as the mean ± SD of three independent experiments. *P < 0.05, **P < 0.01, ***P< 0.001, ****P < 0.0001 and non-significant (ns) were determined by one-way ANOVA analysis with Tukey’s test. **C**, **D** Analysis of cell invasion through a Matrigel-coated membrane for Elovl5-depleted MCF-7 (**C**) or 4T1 (**D**) breast cancer cells treated with DGAT inhibitors (DGAT1i and DGAT2i) relative to their respective breast cancer cells treated with a control shRNA or siRNA (ctrl) and vehicle (DMSO). Data are the mean ± SD of three independent experiments with **P < 0.01 and ****P < 0.0001 determined by one-way ANOVA analysis with Tukey’s test. Representative images are shown. **E** QuPath analysis for quantification of lung surface with metastases in female NMRI-nude mice with tail vein injection of shRNA Elovl5 or shRNA control (ctrl) MCF-7 cells. DGAT1 inhibitor (10 µM) was loaded in MCF-7 cells 24 h before injection. Lungs were collected 35 days post injection. Data represent the mean ± SEM with *P < 0.05, **P < 0,01 and ***P < 0.001 determined by Kruskall–Wallis analysis with a Dunn’s multiple comparison test. Representative images are shown.

We evaluated the impact of LD reduction on the expression of EMT markers in breast cancer cells with Elovl5 knockdown. A mesenchymal phenotype with an upregulation of vimentin and downregulation of epithelial markers (E-cadherin and occludin) was obtained in breast cancer cells with stable or transient silencing of Elovl5 expression as assessed at 48 h by western blotting (Fig. 7A) and RT-qPCR (Fig. 7B) while the treatment with DGAT inhibitors (DGAT1i and DGAT2i) reversed the EMT triggered by Elovl5 extinction in breast cancer cells. Furthermore, we clearly confirmed that ablation of Elovl5 expression with siRNA or shRNA in breast cancer cells inhibited cell proliferation (Supplementary Fig. S7A–C) and promoted cell invasion (Fig. 7C, D and Supplementary Fig. S7D). Nevertheless, the prevention of LD accumulation using DGAT inhibitors (DGAT1i and DGAT2i) in Elvol5-depleted 4T1 and MCF-7 cells restored cell proliferation (Supplementary Fig. S7A–C) and decreased cell invasion (Fig. 7C, D and Supplementary Fig. S7D). To determine the role of Elovl5 downregulation-induced LD accumulation in lung metastasis, we pre-treated shRNA ctrl or shRNA Elovl5 MCF-7 cells with DGAT1 inhibitor (DGAT1i, 10 µM) or vehicle (DMSO) for 24 h and then injected DGAT1i-treated MCF-7 cells in the tail vein of female Nude NMRI mice. Lungs were collected 35 days after injection of MCF-7 cells, and we confirmed that shRNA Elovl5 MCF-7 cells led to the development of more metastases in lungs compared to shRNA ctrl MCF-7 (Fig. 7E and Supplementary Fig. S7E). However, the repression of LD accumulation by DGAT1i loading in shRNA Elovl5 MCF-7 reduced the number of lung metastases (Fig. 7E and Supplementary Fig. S7E). Collectively, the data demonstrate that LD accumulation induced by Elovl5 downregulation controls TGF-β receptor upregulation and drives metastasis.

## Discussion

In this study, we demonstrated a role of Elovl5 in breast cancer growth and metastasis through the expression of TGF-β receptors, mediated by the storage of fat in lipid droplets. Our results showed that the downregulation of Elovl5 expression inhibited the proliferation of breast cancer cells and reduced mammary tumor growth in murine models, which is consistent with a recent publication on prostate cancer [21]. Indeed, the depletion of Elovl5 expression in prostate cancer cells led to inhibition of cell proliferation and metastasis [21]. Interestingly, we demonstrated that the formation of metastases and metastasis-associated features (cell invasion, TGF-β receptor expression and EMT) in breast cancer models were promoted by dampening Elovl5 expression. In contrast, overexpression of Elovl5 in murine 4T1 cells decreased the development of lung metastases. The data obtained from breast cancer patients confirmed a relationship between Elovl5 expression and metastasis. A significant downregulation of Elovl5 expression was observed in patients with metastatic ER^+^ breast cancers (N1) compared to non-metastatic ER^+^ breast cancers (N0). We did not find any differences in Elovl5 expression between patients with metastatic Her2^+^/TNBC breast cancers (N1) compared to non-metastatic Her2^+^/TNBC breast cancers (N0). Of note, Elovl5 expression in patients with an N0 status is already much lower in Her2^+^ and TNBC breast cancer tissues compared to ER^+^ breast cancer tissues and is closer to the downregulated Elovl5 expression in ER^+^ breast cancer tissues from patients with N1 status. It is reported that more than 75% of breast cancer deaths are caused by metastases [23]. Therefore, the correlation between a low expression of Elovl5 and a worse prognosis in patients with ER^+^ breast cancers could be explained by the fact that metastasis is promoted by Elovl5 silencing. In contrast, low expression of Elovl5 is not associated with a worse prognosis in Her2^+^ and TNBC patients. The Elovl5 expression as a prognostic biomarker in Her2^+^ and TNBC patients using the METABRIC database must be carefully interpreted since the analysis was performed on small TNBC and Her2^+^ cohorts (only 55 TNBC and 129 Her2^+^ patients). Moreover, we cannot exclude that Elovl5 interacts with a peculiar oncogenic pathway according to the breast cancer subtype and modifies the tumor cell secretome which might create a distinct microenvironment for tumor growth and metastasis.

TGF-β signaling exerts ambivalent properties in cancer with tumor suppressive effects at early stages and pro-metastatic action at a later stage [25]. TGF-β signaling has the ability to induce cell cycle arrest and/or apoptosis as well as to promote EMT and cell invasion in cancer cells, including breast cancer cells [26, 27]. Active TGF-β elicits its biological effects by binding to serine/threonine kinase TGF-β receptors (TGFβ-R) 1 and 2 directly or with the help of the accessory TGFβ-R3. Binding of TGF-β on the receptor complex triggers TGFβ-R2-induced transphosphorylation and activation of TGFβ-R1. Then, the canonical TGF-β transduction signal depends on TGFβ-R1-mediated phosphorylation of Smad2 and Smad3. The phospho-Smad2/3 heterodimer complex interacts with Smad4 for nuclear translocation of the heterotrimeric complex which runs a transcriptional program [25]. We demonstrated an increase in the expression of TGF-β isoforms and receptors in breast cancer cells lacking Elovl5 expression. Furthermore, Elovl5 depletion induced an increase in phospho-Smad2/3 content suggesting activation of the TGF-β pathway, and anti-proliferative and pro-metastastic effects of TGF-β signaling which were reversed by the inhibitors of TGFβ-R1 and TGFβ-R2. Metabolic adaptations are triggered during activation of the TGF-β pathway leading to an increase of mitochondrial oxygen consumption [28–30]. Thus, it is not surprising to observe an induction of the mitochondrial oxygen consumption in Elovl5-silenced breast cancer cells. Drugs against TGF-β signaling might offer a therapeutic response to cancer cell dissemination, and several inhibitors of the TGF-β pathway are tested for clinical evaluation [31]. However, the ambivalent role of TGF-β requires a precise selection of eligible patients with advanced breast cancer. Furthermore, dosing of anti-TGF-β signaling drugs must be personalized due to possible cardiac and cutaneous side effects [31, 32]. Elovl5 expression might be a predictive biomarker and help to determine eligible recipients of anti-TGF-β signaling therapy.

Alteration of lipid metabolism contributes to cancer progression and pre-clinical studies demonstrated the rationale of targeting lipid metabolism [13, 33, 34]. Elongation of fatty acid is under the control of seven fatty acid elongases (Elovl) and expression of Elovl1 or Elovl6 is increased in breast cancer tissues [19]. Yamashita et al. also described the increase of Elovl5 mRNA expression in breast cancer tissues while they did not find any changes in protein levels of Elovl5 in IHC staining comparing breast tumors to normal paired tissues [19]. In our study, we were able to show a decrease of Elovl5 expression by RT-qPCR as well as by IHC staining in breast cancer tissues. A consequence of Elovl5 downregulation is an accumulation of LD in breast cancer cells which controlled their proliferation and invasion. The increase in the total FA and TAG content in breast cancer cells upon a depletion of Elovl5 was consistent with an activation of lipogenesis and LD formation. The main primary product of de novo lipogenesis is the saturated C16:0 FA which drastically increased in LD from Elovl5-silenced MCF-7 and can accumulate as C16:0-TAG protecting the cells from lipotoxicity [35]. The blockade of Elovl5-dependent elongation of fatty acids in breast cancer cells also induced an accumulation of C24:6-30:6 FA in the cellular FA fraction. The elongation of the C30:6 n-3 FA by Elovl4 led to the biosynthesis of C32:6-C34:6 and to their newly identified di-hydroxylated derivatives (elovanoids) [36]. The Elovl4 silencing-dependent decrease of C32:6 and C34:6 fatty acids triggered a reduction of lipid-droplet number in neuroblastoma cells while the accumulation of LD is associated with a better prognosis [37]. However, the increase of LD abundance is more frequently correlated with an aggressive cancer [38–41] and breast cancer cell lines show an increasing trend in LD content when going from the non-malignant MCF10A cells, to the intermediately aggressive MCF-7 cells to the highly aggressive MDA-MB-231 cells [42]. In accordance with these observations, we demonstrated that the LD accumulation in breast cancer cells is essential for the Elovl5 silencing-mediated promotion of EMT and invasion since a repression of LD formation by a blockade of DGAT1 or DGAT2 activity reversed the induction of EMT and cell invasion. These results highlighted that DGAT1 or DGAT2 have nonredundant properties and play a key role in LD biogenesis. Interfering with exogenous lipid uptake or lipogenesis also results in a decrease of LD abundance and affects the migratory and invasive properties of cancer cells [43–45]. Interestingly, the prevention of LD accumulation in Elovl5-silenced MCF-7 cells by a treatment with DGAT1 inhibitor reduced lung metastases in mice highlighting a therapeutic role of anti-lipid-droplet drugs against breast cancer metastasis. Moreover, LD are able to manage cell lipotoxicity and are a reservoir of FA for acetyl-CoA production during fatty acid oxidation [46]. Thus, we indicated that Elovl5-depleted breast cancer cells increased their mitochondrial oxygen consumption and contained more acetyl-CoA while the abrogation of LD formation by DGAT inhibitors deprived acetyl-CoA levels. The availability in acetyl-CoA contributes to the induction of TGF-β signaling through Smad2 activation by acetylation, impacting EMT and metastasis [15, 44] and this is in line with our data indicating that LD induced by downregulation of Elovl5 expression mediated upregulation of EMT and cell invasion. Finally, we evidenced that Smad2 acetylation also regulates the expression of TGF-β receptors since silencing of Smad2 expression in Elovl5-depleted MCF-7 cells prevented their overexpression.

This work suggests that treatment with DGAT inhibitors could block LD formation and the subsequent TGF-β signaling through repression of TGF-β receptor expression in Elovl5-depleted breast cancer cells. As we discussed above, drugs targeting components of TGF-β signaling (i.e., TGF-β isoforms or TGF-β receptors) have an interesting potential against metastasis. However, the use of DGAT inhibitors might also improve the efficacy of anti-cancer drugs for the treatment of patients with a high risk of metastases associated to low levels of Elovl5 by suppressing the resistance or buffering of these anti-cancer drugs by LD [47–49].

**Fig. 8:**
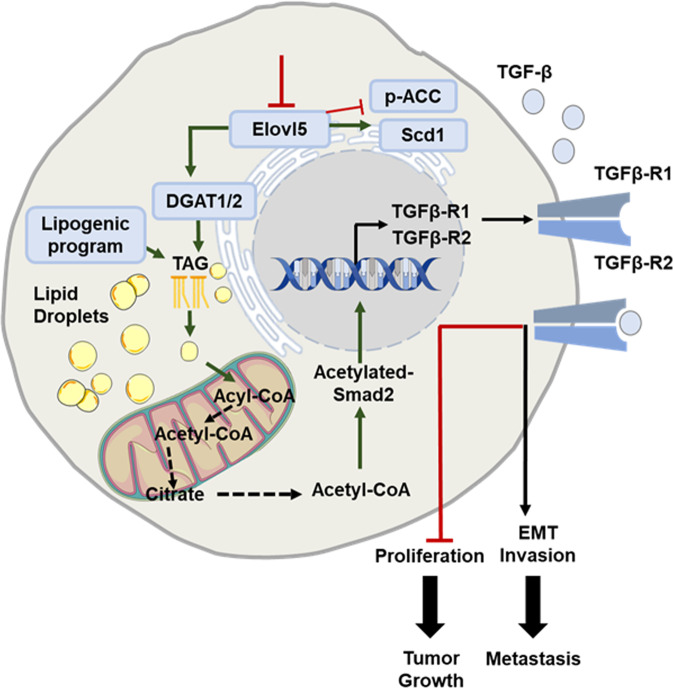
Schematic illustration depicting the role of Elovl5 in the regulation of breast tumor growth and metastasis. A downregulation of Elovl5 expression induced a lipogenic program (decrease of p-ACC expression and increase of Scd1 expression) supporting an increase of fatty acid content. In addition, the loss of Elovl5 expression activated the DGAT1/2 expression and the synthesis of triacylglycerols (TAG) resulting in a lipid-droplet accumulation. The content of Acetyl-CoA increased in a DGAT1/2-dependent manner triggering the acetylation of Smad2 which upregulated the expression of TGF-β receptors 1 and 2. The activation of TGF-β receptors contributed to the repression of cell proliferation and to the induction of metastatic properties (EMT and invasion).

In conclusion, we demonstrate the role of Elovl5 in breast cancer cells through a regulation of lipid-droplet content and expression of TGF-β receptors (Fig. 8). Furthermore, this study proves to be very interesting from a clinical perspective by suggesting a predictive potential of Elovl5 expression as prognosis marker in the risk of development of metastases in patients with breast cancer and by describing a therapeutic potential for drugs preventing the formation of lipid droplets in cancer cells.

## Materials and methods

### Cell culture

Human adenocarcinoma MCF-7 (HTB-22-ATCC), MDA-MB-231 (HTB-26-ATCC), mouse 4T1 mammary carcinoma (CRL-2539-ATCC) and mouse NMuMG mammary cystadenoma (CRL-1636-ATCC) cell lines were used within the first 25 passages and tested for mycoplasma contamination (Lonza, Mycoalert Detection Kit, and Mycoalert Assay Control set). MDA-MB-231 and 4T1 cell lines were maintained in RPMI supplemented with 2 mM l-glutamine, 10% heat-decomplemented FBS, and antibiotics (Penicillin, streptomycin, and amphotericin B). MCF-7 and NMuMG cell lines were maintained in DMEM supplemented with 2 mM l-glutamine, 10% heat-decomplemented FBS, and antibiotics (Penicillin, streptomycin, and amphotericin B). Cells were grown in a humidified 5% CO_2_ incubator at *37* *°*C.

### Lentivirus production and MCF-7 cell line transduction

The pLKO.5-puro lentiviral vectors expressing a control shRNA (SHC202) or an Elovl5-targeting shRNA (TRCN0000317892) were purchased from Sigma-Aldrich. Lentivirus was produced in HEK293T cells by transfection using Lipofectamine 2000 transfection reagent (ThermoFisher Scientific) of VSV-G envelope and PAX2 packaging vectors (Addgene). Lentivirus was collected 48 h after transfection for transduction of MCF-7 cells with 8 μg/mL of polybrene (Sigma-Aldrich). After 2 days, transduced MCF-7 cells were selected with 2 μg/ml puromycin (Fisher Scientific) to establish a control MCF-7 cell line or cell line with a stable Elovl5 knockdown.

### Elovl5-overexpressing 4T1 cell line

Linearized empty pcDNA3.1 or pcDNA3.1 encoding the ORF of the murine Elovl5 gene sequence (Genscript) were transfected into 4T1 cells using Lipofectamine 2000 transfection reagent (ThermoFisher Scientific) according to the manufacturer’s protocol. Two days post transfection, non-clonal 4T1 cell lines that have stably integrated the plasmids were selected using 600 μg/ml G418 sulfate (Euromedex).

### RNA interference and treatments with inhibitors

Cells were reverse-transfected using Lipofectamine™ RNAiMAX transfection reagent (ThermoFisher Scientific) with non-targeting siRNA (#4390846; Dharmacon), siRNA targeting human ELOVL5 (#s34075; Dharmacon), mouse ELOVL5 (#s87101; Dharmacon) or human smad2 (#s8397; Dharmacon) according to the manufacturer’s instructions. Treatments with 10 µM A922500 (Sigma-Aldrich), 10 µM PF-06424439 (Sigma-Aldrich), 1 µM LY2157299 (Selleck) and 1 µM LY2109761 (Sigma-Aldrich) were performed in complete culture medium. Breast cancer cells were incubated with 5 ng/ml recombinant human TGF-β1 (Miltenyi Biotec) in complete cell culture medium for the indicated times.

### Total fatty acid analysis by gas chromatography-mass spectrometry (GC-MS)

Cell lines and tissue samples were homogenized, and lipids were extracted in 1 ml of ethanol/butylated hydroxytoluene (50 mg/ml); 60 μl of 10 10 M potassium hydroxide, and 25 μl of SI-internal standard (#SI S20-040; CDN Isotopes-Cayman) were added to the prepared-suspensions followed by saponification at 56 °C for 45 min. The aqueous phase was collected after the addition of 1 ml of 1.2 M hydrochloric acid and 3 ml of hexane. This was evaporated in a vacuum dry centrifuge at 100 °C, 2200 × *g*. The dry residues were resuspended in 10% v/v N, N-diisopropylethylamine, 10% v/v pentafluorobenzyl bromide diluted in acetonitrile and incubated at 37 °C for 30 min to obtain pentafluorobenzyl esters. In all, 1 ml water and 2 ml hexane were used to support the esters extraction. The hexane phase was collected and evaporated with liquid nitrogen. The fatty acid ester pellets were solubilized in hexane, and 1 µl was injected into a GC-MS system (#7890A Gas Chromatograph, #7683 injector, #5975C Mass Selective Detector; Agilent Technologies) in pulsed split mode by using fused silica capillary column (#HP-5MS; Agilent Technologies). GC-MS running-mode contained helium gas with a flowrate at 1.8 ml/min, injector temperature at 250 °C, pulsed split 10, the oven temperature was set at 140 °C for 1 min and then increased at a rate of 5 °C/min, then left at 300 °C for 7 min. The machine was set in negative chemical ionization mode, in the presence of methane as the reactant gas, with the temperature of ion source and quadrupole set at 150 °C. All the experimental steps were performed in glass-based containers. The FAs were first normalized using the closest internal standard by relative ratios using MassHunter and Qualitative software (Agilent Technologies). These FAs were then normalized to the total cell counts and the protein concentration from the initial sample.

### Lipid-droplet isolation

To perform the lipidomic analysis on lipid droplet, MCF-7 cells were washed and harvested. After normalization of each sample by cellular counting, the lipid droplets were extracted by using lipid-droplet isolation kit (MET-5011; Cell Biolabs) according to the manufacturer’s instructions. Floating lipid-droplet samples were collected for further lipidomic analysis.

### Triacylglycerol (TAG) quantification

Cells and tissue samples were homogenized in one volume of methanol. Four volumes of dimethoxymethane were added, and the samples were continuously vortexed for 1 h. The organic solvents were evaporated under N2 flow, and TAG were resuspended in CHCl3-2% v/v Triton X-100. After CHCl_3_ evaporation, deionized water was added to the Triton X-100 and TAG solution before TAG quantification using a Triglycerides FS enzymatic assay kit (Diagnosis systems) according to the manufacturer’s instruction. TAG content was normalized to the protein content of the initial sample, obtained by BCA assay quantification.

### Acetyl-CoA quantification

Total cell lysates were collected and deproteinized using the Deproteinization kit (#ab204708; Abcam). Intracellular Acetyl-CoA content was determined using the Acetyl-CoA assay kit (#PicoProbe ab87546; Abcam), following the manufacturer’s instructions. Acetyl-CoA concentration was normalized to the protein content of the corresponding samples.

### Bodipy 493/503 staining

Cells on coverslips were fixed with 4% paraformaldehyde (PFA) for 10 min at RT and incubated with 10 μM of Bodipy 493/503 (ThermoFisher Scientific) solution for 30 min. After washing, coverslips were mounted with ProlongTM diamond antifade mountant (Molecular Probes) containing 20 µM Hoechst (ThermoFisher Scientific). The detection of lipid droplets using Bodipy 493/503 was carried out using 470-nm excitation and 525-nm emission wavelengths. The staining was observed with Axio Imager 2 (Carl Zeiss Microscopy GmbH, Jena, Germany) connected to an Apotome 2 module (Carl Zeiss GmbH). Images were taken with a AxioCam MRm monochrome CCD camera (Carl Zeiss GmbH). The lipid-droplet number was determined by assessing the number of total lipid droplets and the cell number (minimum 100 cells per experimental condition) in the field for the calculation of the average lipid-droplet number per cell. In addition, a quantification of the average fluorescence intensity per cell was performed in the randomly recorded fields using imageJ software.

### Oil Red O staining

Mouse mammary tumors were collected in Optimal Cutting Temperature compound (OCT) (ThermoFisher Scientific) and placed on dry ice. The OCT tumor samples were fixed with 4% PFA for 10 min at RT. The fixed OCT sections were stained with 0.3% Oil Red O solution for 30 min. After washing, the tumor sections were stained with hematoxylin and mounted with an aqueous mounting solution.

The score of lipid droplets in tumors was performed using QuPath 0.2.3 software [50] after scanning the slides with a Nanozoomer using the NDP.view scan software. Data are presented as the percentage of positive lipid-droplet cancer cells over total breast cancer cells in the tumoral zone.

### Phalloidin staining

Cells on coverslips were washed with cold PBS and fixed with 4% PFA for 10 min at RT. After cell permeabilization with 0.1% v/v Triton X-100 (Euromedex) in PBS, cells were incubated with FITC-Phalloidin (50 μg/ml) for 40 min. After washing with PBS, cells were mounted with ProlongTM diamond antifade mountant (Molecular Probes) containing 20 µM Hoechst (ThermoFisher Scientific) and observed with Axio Imager 2 (Carl Zeiss Microscopy GmbH, Jena, Germany) connected to an Apotome 2 module (Carl Zeiss GmbH). Images were taken with an AxioCam MRm monochrome CCD camera (Carl Zeiss GmbH). The quantification of cellular morphology modification was based on the ratio between the number of cells with an epithelial-like morphology and the number of cells with a mesenchymal-like morphology in the recorded fields.

### Immunofluorescence staining of TGF-β receptors

For fluorescence microscopy analysis, cells were grown and treated on coverslips, washed with PBS, and fixed with 4% PFA for 10 min. Cells were permeabilized with 0.2% saponin (Sigma-Aldrich) in PBS and saturated with 3% BSA in PBS for 20 min at RT. Cells were incubated with a primary antibody against TGF-β R1 (PA5-3871; Invitrogen) overnight at 4 °C in 3% BSA in PBS. Samples were incubated with an Alexa568-conjugated anti-rabbit antibody (Invitrogen) for 30 min at RT in 3% BSA in PBS. Coverslips were mounted with ProlongTM diamond antifade mountant (Molecular Probes) containing 20 µM Hoechst (ThermoFisher Scientific) and observed with Axio Imager 2 (Carl Zeiss Microscopy GmbH, Jena, Germany) connected to an Apotome 2 module (Carl Zeiss GmbH). Images were taken with AxioCam MRm monochrome CCD camera (Carl Zeiss GmbH).

For flow cytometry analysis, cells were washed in cold PBS and collected by scraping, then saturated with 10% FBS in PBS for 10 min. After a washing step, cells were stained for 40 min on ice with an isotype control IgG-PE (Biolegend), or an anti-mouse TGF-β R2-PE (R&D Systems) or an anti-human TGF-β R2-PE (Biolegend). Flow cytometry analysis was performed with a BD LSR II flow cytometer (BD Biosciences). For TGF-β R1 staining, human and mouse cells were incubated with anti-TGF-β R1 (Merck Millipore) for 40 min on ice, and after a washing step, cells were incubated with an isotype control Alexa568-conjugated IgG or a secondary Alexa568-conjugated anti-rabbit antibody (Invitrogen) for 20 min at RT. Cells were washed and collected in PBS for analysis on an LSR Fortessa cytometer and the BD FACSDiva software (BD Biosciences). Data were analyzed using the FlowJo software (Tree Star, USA) and presented in the mean of fluorescence intensity.

### RNA extraction and RT-qPCR

Total RNA was extracted using TRIzol (Invitrogen) according to the manufacturer’s instructions. Reverse transcription was performed with an iScript cDNA Synthesis Kit (Bio-Rad). PCR was performed using Powerup SYBR Green master mix (Applied Biosystems, Life Technologies) on StepOnePlus™ Real-Time PCR System (Applied Biosystems). Relative gene expression was calculated using ΔΔCt values. Target mRNA levels were normalized to β-actin mRNA and 18 S RNA. Primers (Supplementary Table S5) were purchased from Life technologies.

### Cell proliferation assay

Cells were washed with PBS and fixed with 100% cold ethanol. Coloration with 4% crystal violet in 10% methanol was carried out to visualize adherent cells. After washing with water, crystal violet was dissolved in 33% acetic acid for OD quantification on a TECAN spectrophotometer at 540 nm.

### Colony-formation assay

Cells were plated at low density (150 4T1 cells and 200 MCF-7 cells/well) in six-well plates. The culture medium was renewed every 3 days. Cells were fixed and stained with crystal violet for counting of colonies after 1 week for 4T1 cells and 3 weeks for MCF-7 cells. Data were presented as an average of total counted colonies per well.

### Oxygen consumption rate

The analysis of the real-time Oxygen Consumption Rate was performed in Elovl5-downregulated expression MCF-7 cells (4 × 10^4^ cells/well) with a Seahorse XFe96 Extracellular Flux Analyzer (Agilent Technologies). The measure under basal conditions and with the sequential addition of respiratory chain complex inhibitors (Mito Stress assay [oligomycin, FCCP, antimycin A/rotenone]; Agilent Technologies) were carried out in DMEM-based medium (without glucose, pyruvate and l-glutamine) according to the manufacturer’s instructions. OCR data were normalized per µg of protein per well.

### Western blotting

Cells were lysed in RIPA supplemented with protease (#P8340; Sigma-Aldrich) and phosphatase (#P5726; Sigma-Aldrich) inhibitors. Protein lysates in Laemmli loading buffer were boiled for 5 min at 95 °C and separated by SDS-PAGE electrophoresis. Proteins were wet-transferred onto nitrocellulose membranes (AmershamTM ProtranTM 0.45-µm NC; Amersham). Membranes were saturated with 5% w/v non-fat milk or 5% BSA in TBST (Tris-buffered saline plus 0.1% Tween 20) for 1 h at RT. Primary antibodies (listed in Supplementary Table S6) were diluted in the appropriated saturation solutions for overnight incubation at 4 °C. After TBST washes, membranes were incubated with horseradish peroxidase (HRP)-conjugated secondary antibodies (1:5000^eme^; Cell Signaling Technologies). Membranes were images using Enhanced Chemi Luminescence (ClarityTM Western ECL Substrate; Bio-Rad) in a sChemiDocTM XRS ^+^ imaging system (Biorad). Images were analyzed with Image Lab 5.1 software (Biorad, France).

### Co-immunoprecipitation

Cells were lysed in non-denaturing lysis buffer (20 mM Tris HCl pH 8.0, 137 mM NaCl, 10% glycerol, 2 mM EDTA and 1% Triton X-100) supplemented with protease (#P8340; Sigma-Aldrich) and phosphatase (#P5726; Sigma-Aldrich) inhibitors. Pre-clearing step of 1 mg cell lysates were performed with 5 µg rabbit IgG (#02-6102; ThermoFisher Scientific) and 20 µl Pierce Protein A/G Magnetic beads (#88802; ThermoFisher Scientific) on a rotating shaker for 1 h and 30 min, at 4 °C. The lysates were then incubated overnight at 4 °C with 5 µg of rabbit IgG or anti-acetylated lysine (#9441; Cell Signaling Technology). The immunoprecipitated proteins were collected after 1h30 incubation with 20 µl Pierce Protein A/G Magnetic beads (4 °C), followed by the elution with 50 µl of Elution buffer (0.1 M Glycine, pH 2.0) and neutralization with a buffer containing 1 M Tris HCl, pH 8.0. Samples were added 1× Laemmli loading buffer β-mercaptoethanol-free, without heating, and separated by SDS-PAGE electrophoresis. As described above, western blotting was performed using anti-Smad2 antibody (#5339; Cell Signaling Technology; 1:1000).

### Invasion assay

Cell invasion assay was carried out in Transwell insert (Greiner Bio-One) with a Matrigel-coated 8-µm pore membrane (#356237; Corning). Cells in culture medium with 0.1% FBS were placed on top of the Matrigel-coated inserts, and complete culture medium (10% FBS) was added in the lower chamber. After 48 h, cells were fixed with 4% PFA and stained with a crystal violet solution (Sigma-Aldrich) for 30 min. The inserts were washed to remove non-invasive cells. Images of randomly selected fields were recorded by an AxioScope A1 microscope (ZEISS Microscopy) coupled camera (Jenoptik; GRYPHAX NAOS) using the GRYPHAX software. Cell counting was performed using ImageJ software.

### Immunohistochemistry analysis

Paraffin-embedded tumor breast tissue samples from breast cancer patients with or without metastasis were deparaffinized and rehydrated. Tissue sections were saturated with peroxidase blocking buffer and incubated with a primary antibody against ELOVL5 (#HPA047752; Sigma-Aldrich) for 1 h. After washing, HRP polymer-conjugated secondary antibodies (K4002; Dako) were then incubated with the tissue sections for 20 min followed by a 5-min coloration with 1 g/ml of DM827-3, 3’-diaminobenzidine (DAB). Hematoxylin and eosin staining was used to visualize nuclei. Tissue sections were mounted with an organic mounting solution from Dako. Quantification of ELOVL5 staining was analyzed with QuPath 0.2.3 [50] after slides were scanned by Nanozoomer controlled by NDP.view scan software (Hamamatsu). Data are presented as the average H-score of ELOVL5 expression in each slide comparing tumor and adjacent non-tumoral tissues.

For Ki67 staining, the protocol is as above using a primary antibody against Ki67 (#MIB-1 clone; Agilent) and an anti-mouse HRP polymer-conjugated secondary antibody (K4000; Dako). The H-score correlations between ELOVL5 and Ki67 are demonstrated by comparing the tumoral matching zones of the same patient.

### MMTV-PyMT and Elovl5 mice

Mice were bred and maintained in the animal facility of the University of Burgundy according to the center instructions, all the experiments were carried out following the instructions of the Federation of European Animal Science Associations. Experimental designs involving animals were approved by the Ethics Committee of the University of Burgundy (projects #17461, #14557 and #22358). *Elovl5*^*−/−*^ C57BL/6N mice were obtained from the Mutant Mouse Regional Resource Center at UC Davis (Davis, CA) and were generated by inserting a genetrap cassette in the exon 3 of the *Elovl5* gene. B6.FVB-Tg (MMTV-PyVT)634Mul/LellJ (MMTV-PyMT) mice were obtained from the Jackson Laboratory. Male MMTV-PyMT mice were crossed with female Elovl5^-/-^ to obtain MMTV-PyMT;Elovl5^+/−^ mice. Then, experimental female mice were obtained by crossing male MMTV-PyMT;Elovl5^+/-^ mice with female Elovl5^−/+^ mice. Mouse genotyping was performed using extraction solution (E7526; Sigma-Aldrich) and neutralization solution B (N3910; Sigma-Aldrich) and with PCR primer sets (Supplementary Table S5) for the detection of MMTV-PyMT transgene and Elovl5 invalidation. Female mice were monitored by palpation and tumor growth measured every 2 days. Tumor surface (mm^2^) were calculated by multiplying the measured length and width of the tumor. Upon reaching 180 days of age, mice were sacrificed, and tissues (lung and tumors) were collected for analysis.

### Mammary gland grafting and tail vein injection

4T1 cells (0.5 × 10^6^ cells in 100 µl PBS per mice) were injected into the inguinal mammary fat pad of 8-week-old mice (BALB/cByJ; Charles River) using a 26Gx7/8” needle and 1 ml syringe. Tumors were measured three times per week and tumor size (mm^2^) was calculated by multiplying the measured length and width of the tumor. On the 23rd day post injection, mice were sacrificed, and tumors and lungs were collected for downstream experiments.

For tail vein injection of MCF-7 cells, female 8-week-old nude mice (NMRI; Charles River) were pre-treated with β-estradiol (Sigma-Aldrich) at 4 µg/100 µl PBS/mice by intraperitoneal injection 24 and 48 ho before cell injection. Elovl5 knockdown and control MCF-7 cells (5 × 10^6^ cells in 100 µl PBS per mice) were injected into the tail vein using insulin syringes (30 G × 1/2”). Injection with β-estradiol were carried out three times/week. Mice were sacrificed 35 days after MCF-7 cell injection, and lungs were collected for metastatic quantification.

For tail vein injection of 4T1 cells, 1 × 10^5^ 4T1 cells in 50 µl PBS per mice were injected into the tail vein of 8-week-old mice (BALB/cByJ; Charles River) by using insulin syringes (30 G × 1/2”). The mice were sacrificed 11 days post-cell injection and lungs were collected for metastase analysis.

Lungs were fixed in formalin and paraffin-embedded. Tissues were dewaxed and rehydrated for hematoxylin and eosin staining. Tissue sections were then mounted with an organic mounting solution. Scoring the invasive tumoral area over the total lung area was performed using QuPath 0.2.3 software after scanning the slides with a Nanozoomer using the NDP.view scan software (Hamamatsu). Data are presented as the ratio of total tumor area over the total lung area.

### Patients and METABRIC datasets

Research on patient samples was conducted in agreement with the Declaration of Helsinki and approved by the Ethics Committee of the Centre Georges-François Leclerc, University Hospital François Mitterrand Dijon-Bourgogne (Dijon, France), and the Burgundy Advisory Committee for the Protection of People in Biomedical Research. The patient’s informed consent was obtained before project enrollment. The clinical and pathological characteristics of patients are summarized in Supplementary Tables 2–4.

Discovery Metabric datasets, generated by the Molecular Taxonomy of Breast Cancer International Consortium, was used as a public dataset. Normalized data, obtained with the Illumina HT 12 platform, were requested, and downloaded from the European Genome-phenome Archive (EGA) under the identifiers EGAD00010000210 and EGAD00010000211. Overall survival and molecular classification were available.

### Statistical analysis

Statistical analyses were performed with the GraphPad Prism 9.1 software. After verifying data for normal distribution and variance, we applied one of the following statistical tests: Mann–Whitney test, Student’s *t* test or one-way ANOVA. *P* values were determined as statistically significant with *P < 0.05; **P < 0.01; ***P < 0.001; ****P < 0.0001. Data are presented as mean ± SD or SEM.

## Supplementary information

Supplementary Figures S1-7 and Legends of Figures

Table S1

Table S2

Table S3

Table S4

Table S5

Table S6

Supplementary Materials and methods

Original western-blots

Reproducibility Checklist:

## Data Availability

The data generated or analyzed during this study are available from the corresponding author upon request.
