# Supplementary material for: Downregulation of Elovl5 promotes breast cancer metastasis through a lipid-droplet accumulation-mediated induction of TGF-β receptors
Source: Cell Death Dis. 2022 Sep 2;13(9):758. doi: 10.1038/s41419-022-05209-6 (PMC9440092; doi:10.1038/s41419-022-05209-6)
Supplement: Supplementary file 1 — Supplementary Figures S1-7 and Legends of Figures [file 41419_2022_5209_MOESM1_ESM.pdf]

Fig. S1

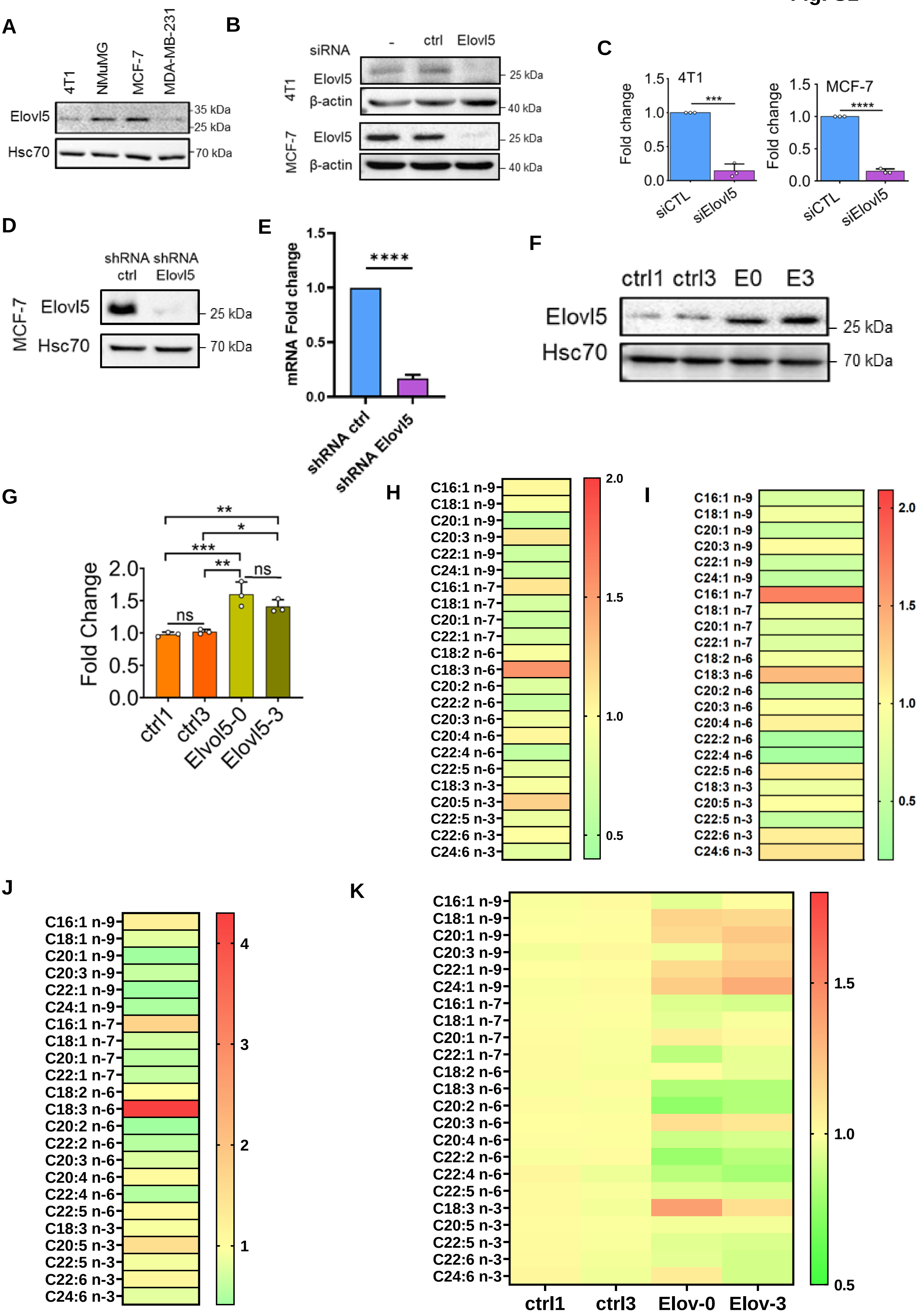
A
4T1
NMuMG
MCF-7
MDA-MB-231
Elovl5
Hsc70
35 kDa
25 kDa
70 kDa
B
siRNA
-
ctrl
Elovl5
4T1
Elovl5
β-actin
MCF-7
Elovl5
β-actin
25 kDa
40 kDa
25 kDa
40 kDa
C
4T1
Fold change
***
siCTL
siElovl5
MCF-7
Fold change
****
siCTL
siElovl5
D
shRNA ctrl
shRNA Elovl5
MCF-7
Elovl5
Hsc70
25 kDa
70 kDa
E
mRNA Fold change
****
shRNA ctrl
shRNA Elovl5
F
ctrl1
ctrl3
E0
E3
Elovl5
Hsc70
25 kDa
70 kDa
G
Fold Change
**
*
***
**
ns
ns
ctrl1
ctrl3
Elovl5-0
Elovl5-3
H
I
J
K
C16:1 n-9
C18:1 n-9
C20:1 n-9
C20:3 n-9
C22:1 n-9
C24:1 n-9
C16:1 n-7
C18:1 n-7
C20:1 n-7
C22:1 n-7
C18:2 n-6
C18:3 n-6
C20:2 n-6
C22:2 n-6
C20:3 n-6
C20:4 n-6
C22:4 n-6
C22:5 n-6
C18:3 n-3
C20:5 n-3
C22:5 n-3
C22:6 n-3
C24:6 n-3
ctrl1
ctrl3
Elov-0
Elov-3

Figure S1: Validation of breast cancer cell models with modulation of Elovl5 expression. A. Elovl5 expression in murine 4T1 breast carcinoma cells, murine NMUMG normal mammary epithelial cells and human (MCF-7 and MDA-MB-231) breast cancer cell lines analyzed by western-blotting. B-C. Elovl5 expression 48 hours after transfection with a control (ctrl) or Elovl5-targeting siRNA analyzed by western-blotting (B) and RT-qPCR (C). ***p<0,001 and ****p<0,0001 (Student's t test). D-E. Validation of stable Elovl5 extinction using an Elovl5-targeting shRNA Elovl5 compared to a control shRNA (ctrl) in MCF-7 cells analyzed by western-botting (D) and RT-qPCR (E). F. Analysis by western-blotting of stable overexpression of Elovl5 in 4T1 cells (Elovl5-0 -E0- and Elovl5-3 -E3) compared to control (ctrl1 and ctrl3) 4T1 cells. G. Expression of Elovl5 mRNA in Elovl5-overexpressing 4T1 cells (Elovl5-0 and Elovl5-3) and control (ctrl1 and ctrl3) 4T1 cells. *p<0,05, **p<0,01, ***p<0,001 and non-significant (ns) were calculated using a one-way Anova analysis using Tukey's multiple comparison test. H-K. Heatmaps showing the fold change of mono- and polyunsaturated fatty acid content in Elovl5 siRNA-treated MCF-7 cells relative to control siRNA-treated MCF-7 cells (H), in Elovl5 shRNA MCF-7 cells relative to control shRNA MCF-7 cells (I), in Elovl5 siRNA-treated 4T1 cells relative to control siRNA-treated 4T1 cells (J) and in Elovl5-overexpressing 4T1 cells (Elovl-0 and Elovl-3) relative to the mean of control (ctrl1 and ctrl3) 4T1 cells (K).

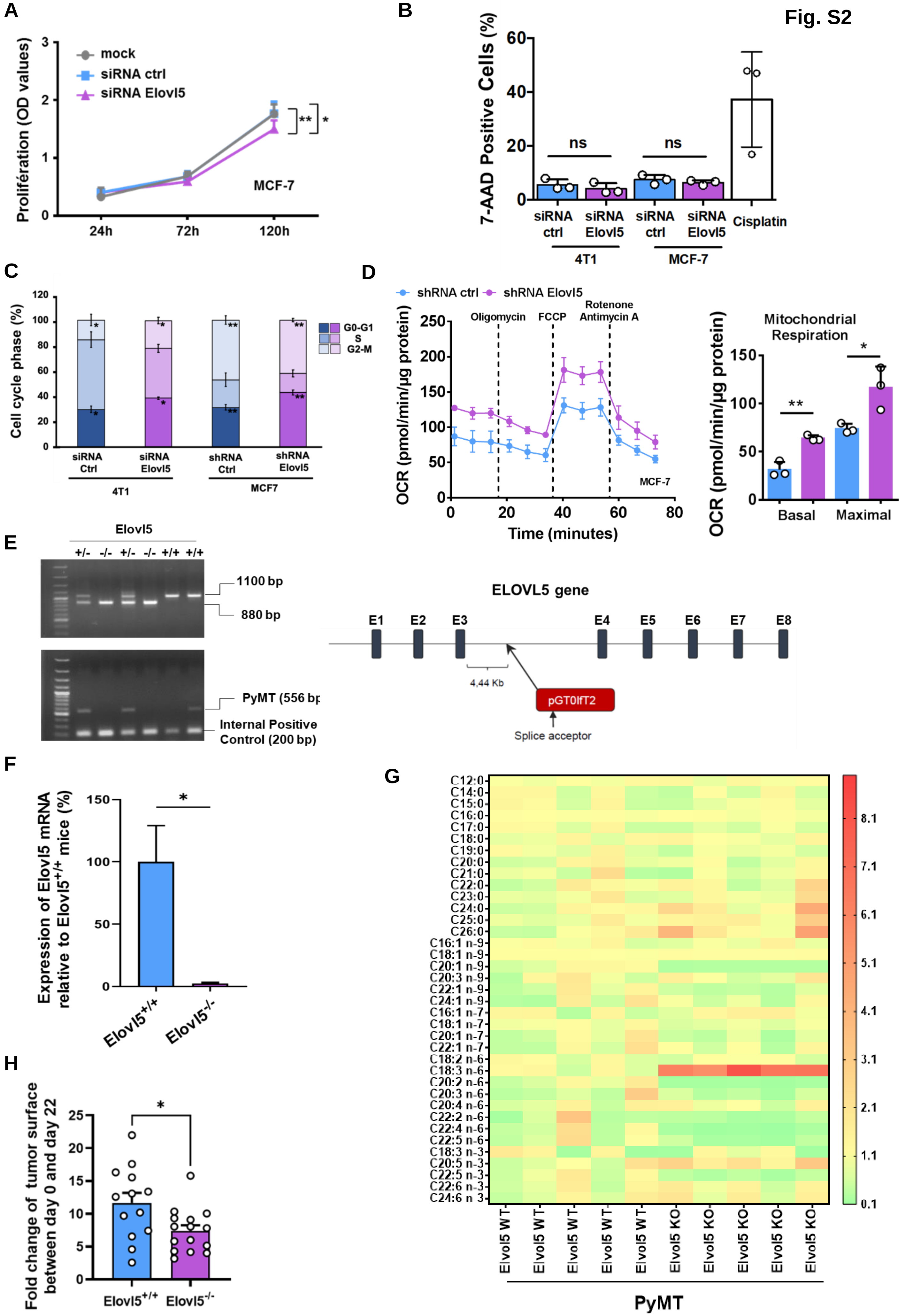

Fig. S2
A
mock
siRNA ctrl
siRNA Elovl5
Prolifération (OD values)
MCF-7
24h
72h
120h
B
7-AAD Positive Cells (%)
ns
siRNA ctrl
siRNA Elovl5
Cisplatin
4T1
MCF-7
C
Cell cycle phase (%)
G0-G1
S
G2-M
siRNA Ctrl
siRNA Elovl5
shRNA Ctrl
shRNA Elovl5
4T1
MCF7
D
shRNA ctrl
shRNA Elovl5
Oligomycin
FCCP
Rotenone Antimycin A
OCR (pmol/min/µg protein)
Time (minutes)
MCF-7
Mitochondrial Respiration
Basal
Maximal
E
Elovl5
+/- -/- +/- -/- +/+ +/+
1100 bp
880 bp
PyMT (556 bp
Internal Positive Control (200 bp)
ELOVL5 gene
E1 E2 E3 E4 E5 E6 E7 E8
4,44 Kb
pGT0lfT2
Splice acceptor
F
Expression of Elovl5 mRNA relative to Elovl5+/+ mice (%)
Elovl5+/+
Elovl5-/-
G
C12:0 C14:0 C15:0 C16:0 C17:0 C18:0 C19:0 C20:0 C21:0 C22:0 C23:0 C24:0 C25:0 C26:0
C16:1 n-9 C18:1 n-9 C20:1 n-9 C20:3 n-9 C22:1 n-9 C24:1 n-9
C16:1 n-7 C18:1 n-7 C20:1 n-7 C22:1 n-7
C18:2 n-6 C18:3 n-6 C20:2 n-6 C20:3 n-6 C20:4 n-6 C22:2 n-6 C22:4 n-6 C22:5 n-6
C18:3 n-3 C20:5 n-3 C22:5 n-3 C22:6 n-3 C24:6 n-3
Elvol5 WT
Elvol5 KO
PyMT
H
Fold change of tumor surface between day 0 and day 22
Elovl5+/+
Elovl5-/-

Figure S2: Proliferation and validation of the MMTV-PyMT;Elovl5 murine model. A. Proliferation of transient Elovl5-silenced MCF-7 cells analysed with crystal violet staining. Error bars represent the mean±SD of independent experiments (n=5 (A) with *p<0.05, ** p<0.01 and ***p<0.001 using a one-way Anova analysis with Tukey test. B. Cell death analyzed by 7-ADD staining in 4T1 and MCF-7 cells treated for 72 hours with siRNA ctrl or Elovl5. Cisplatin treatment was used as a positive control of cell death. C. Cell cycle analysis in control or Elovl5-depleted 4T1 and MCF-7 cells. Histograms and error bars show the mean±SD with $*p<0.05$ and $**p<0.01$ according to Student's t test. D. Oxygen consumption rate measured with Seahorse XFe96 analyzer in stable Elovl5-depleted MCF-7 cells. Error bars represent the mean±SD with $*p<0.05$ and **p<0.01 according to Student's t test. E. *Elovl5* gene targeting strategy. *Elovl5*$^{-/-}$ mice were obtained from the Mutant Mouse Regional Resource Center at UC Davis and were generated by inserting a genetrap cassette in the exon 3 (E3) of the *Elovl5* gene. Genotyping analysis of C57BL/6 mice by PCR. Elovl5 primers amplified a 1100 bp DNA fragment for wild-type allele and a 880 bp DNA fragment for the knockout allele. PyMT primers amplified a 556 bp DNA fragment in mice with the transgene. F. Relative expression of Elovl5 mRNA in mammary tumors of MMTV-PyMT;Elovl5$^{+/+}$ (n=5) and MMTV-PyMT;Elovl5$^{-/-}$ (n=5) mice. Shown are the mean±SEM of 5 mice per group. *p<0.05 defined with Student's t test. G. Heatmap showing the fold change of fatty acid content in MMTV-PyMT;Elovl5 knockout (KO) mice (n=5) relative to MMTV-PyMT;Elovl5 wild-type (WT) mice (n=5). H. Fold change of aggregated tumor surface on day 22 compared to day 0 (day on which the tumor lesion was first palpable and measured) in MMTV-PyMT;Elovl5$^{+/+}$ (Elovl5$^{+/+}$, n=23) and MMTV-PyMT;Elovl5$^{-/-}$ (Elovl5$^{-/-}$, n=20) mice. Fold change for each individual mouse is indicated by dots, the histogram and error bars show the mean±SEM with $*p<0.05$ according to Student's t test.

Figure S3: Development of lung metastases depends on Elovl5 expression. A. Number of metastases on the lung surface of 6-month-old MMTV-PyMT;Elovl5$^{+/+}$ (Elovl5$^{+/+}$, n=13) and MMTV-PyMT;Elovl5$^{-/-}$ (Elovl5$^{-/-}$, n=15) mice. Shown are the mean±SEM with *p<0.05 according to Student's t test. B. Graph representing the number of lung metastases and the cumulative surface of the primary mammary tumor for each 6-month-old MMTV-PyMT;Elovl5$^{+/+}$ (Elovl5$^{+/+}$, n=13) and MMTV-PyMT;Elovl5$^{-/-}$ (Elovl5$^{-/-}$, n=15) mice. C. Representative images of H&E staining of metastases in lungs of Balb-c mice with fat pad injection of Elovl5-overexpressing (Elovl5-0 and Elovl5-3) or control (ctrl1 and ctrl3) 4T1 cells correspondent to Figure 3B. D. Number of metastases on the lung surface of Elovl5-overexpressing (Elovl-0 and Elovl-3) and control (ctrl1 and ctrl3) 4T1 cells transplanted in the fourth fat pad of female Balb-c mice. Shown are the mean±SEM with **p<0,01 and ***p<0,001 values determined by a Kruskall-Wallis analysis with Dunn's test. E. Number of metastases on the lung surface in female NMRI-nude mice with tail vein injection of Elovl5 shRNA and control shRNA-expressing MCF-7 cells. Shown are the mean±SEM with *p<0.05 according to a Mann-Withney test. F-G. QuPath analysis and number of metastases in lungs of female Balb-c mice with tail vein injection of Elovl5-overexpressing (Elovl5-0 and Elovl5-3) or control (ctrl1 and ctrl3) 4T1 cells. Shown are the mean±SEM with **p<0.01, ***p<0,001, ****p<0,0001 and non-significant (ns) according to a Kuskall-Wallis analysis with Dunn's test. H. Representative images of Elovl5 IHC staining for H-score analysis. The red insert shows breast tumor tissue and the blue insert shows normal adjacent tissue. Breast cancer subtypes are indicated.

Fig. S3

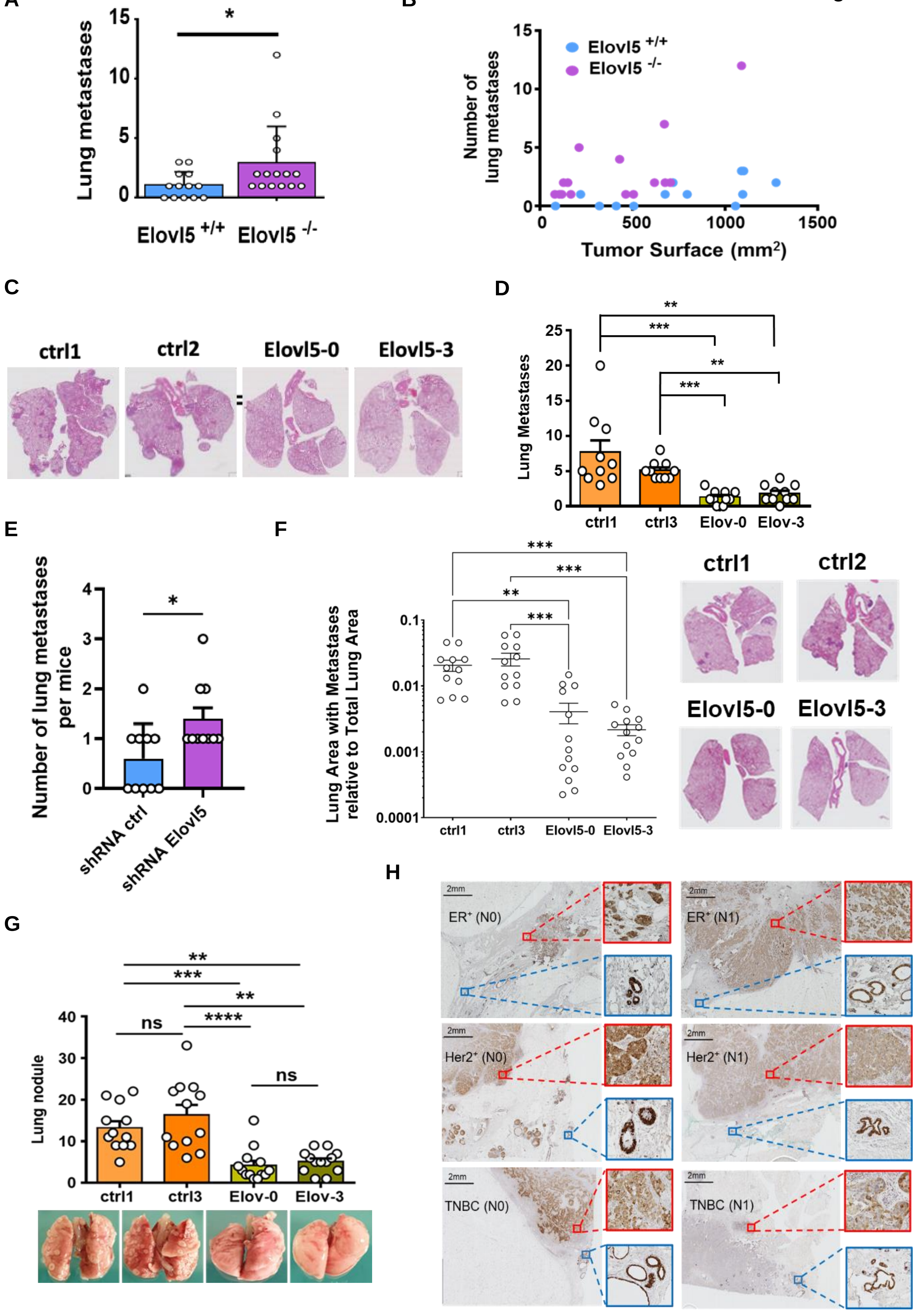
A
Lung metastases
15
10
5
0
*
Elovl5 +/+
Elovl5 -/-
B
Number of lung metastases
Elovl5 +/+
Elovl5 -/-
Tumor Surface (mm2)
0
500
1000
1500
C
ctrl1
ctrl2
Elovl5-0
Elovl5-3
D
Lung Metastases
25
20
15
10
5
0
**
***
**
***
ctrl1
ctrl3
Elov-0
Elov-3
E
Number of lung metastases per mice
4
3
2
1
0
*
shRNA ctrl
shRNA Elovl5
F
Lung Area with Metastases relative to Total Lung Area
0.1
0.01
0.001
0.0001
***
***
**
***
ctrl1
ctrl3
Elovl5-0
Elovl5-3
ctrl1
ctrl2
Elovl5-0
Elovl5-3
G
Lung nodule
40
30
20
10
0
**
***
**
ns
****
ns
ctrl1
ctrl3
Elov-0
Elov-3
H
2mm
ER+ (N0)
ER+ (N1)
Her2+ (N0)
Her2+ (N1)
TNBC (N0)
TNBC (N1)

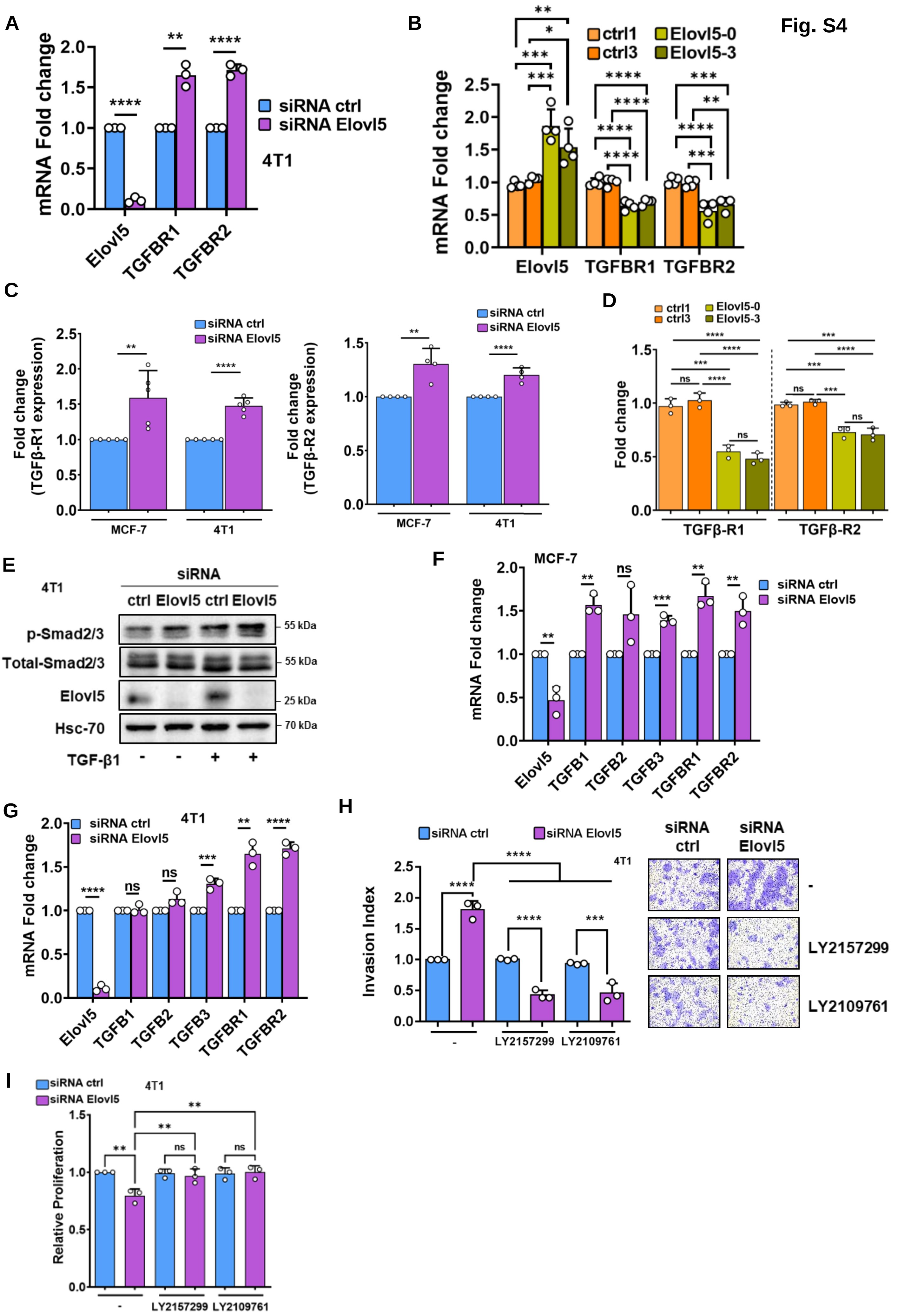
Fig. S4
A
mRNA Fold change
siRNA ctrl
siRNA Elovl5
4T1
Elovl5
TGFBR1
TGFBR2
B
ctrl1
ctrl3
Elovl5-0
Elovl5-3
mRNA Fold change
Elovl5
TGFBR1
TGFBR2
C
Fold change (TGFβ-R1 expression)
Fold change (TGFβ-R2 expression)
siRNA ctrl
siRNA Elovl5
MCF-7
4T1
D
Fold change
TGFβ-R1
TGFβ-R2
E
4T1
siRNA
ctrl Elovl5 ctrl Elovl5
p-Smad2/3
Total-Smad2/3
Elovl5
Hsc-70
TGF-β1
55 kDa
55 kDa
25 kDa
70 kDa
F
MCF-7
mRNA Fold change
Elovl5
TGFB1
TGFB2
TGFB3
TGFBR1
TGFBR2
G
4T1
mRNA Fold change
H
Invasion Index
LY2157299
LY2109761
siRNA ctrl
siRNA Elovl5
I
Relative Proliferation
LY2157299
LY2109761

Figure S4: Regulation of the TGF-β pathway by Elovl5. A. Analysis of TGFBR1 and TGFBR2 mRNA expression at 24 hours by RT-qPCR in Elovl5-silenced 4T1 cells using an siRNA against Elovl5 relative to non-targeting siRNA-treated 4T1 cells. Histograms and error bars represent the mean±SD of three independent experiments with **p<0.01 and ****p<0.0001 according to Student's t test. B. Analysis of TGFBR1 and TGFBR2 mRNA expression at 24 hours by RT-qPCR in Elovl5-overexpressing 4T1 cells (Elovl5-0 and Elovl5-3) relative to the mean of control 4T1 cells (ctrl1 and ctrl3). Histograms and error bars represent the mean±SD of five independent experiments with *p<0.05, **p<0.01, ***p<0.001 and ****p<0.0001 according to one-way Anova analysis with Tukey's multiple comparison test. C. Analysis of TGF-β receptor 1 and 2 expression at 48 hours by flow cytometry in Elovl5-depleted MCF-7 and 4T1 cells treated using an siRNA. Histograms and error bars represent the mean±SD of at least four independent experiments with ***p<0.001 and ****p<0.0001 according to Student's t test. D. Analysis of TGF-β receptor 1 and 2 expression at 48 hours by flow cytometry in Elovl5-overexpressing 4T1 cells (Elovl5-0 and Elovl5-3) relative to the mean of control 4T1 cells (ctrl1 and ctrl3). Histograms and error bars represent the mean±SD of three independent experiments with ****p<0.001, ****p<0.0001 and non-significant (ns) according to one-way Anova analysis with Tukey's multiple comparison test. E. Analysis of Smad2/3 phosphorylation (p-Smad2/3) and total Smad2/3 expression in Elovl5-silenced 4T1 cells treated with TGF-β1 (5 ng/ml) for 30 minutes by western-blotting. F-G. Analysis of TGFB1, TGFB2, TGFB3, TGFBR1 and TGFBR2 mRNA expression by RT-qPCR at 24 hours in Elovl5-silenced MCF-7 (F) and 4T1 (G) cells using an Elovl5 siRNA relative to a control siRNA. Shown are the mean±SD of three independent experiments with non-significant (ns), **p<0,01, ***p<0,001 and ****p<0,0001 according to Student's t test. H. Analysis of cell invasion through a Matrigel-coated membrane for Elovl5-depleted 4T1 cells with transient siRNA against Elovl5 relative to 4T1 treated with a control siRNA (ctrl).

***p<0.001 and ****p<0.0001 were determined by one-way Anova analysis with Tukey’s test. Histograms and error bars represent the mean±SD of three independent experiments. Representative images are shown. I. Analysis of proliferation by crystal violet staining in Elovl5-depleted 4T1 cells using an siRNA against Elovl5 relative to 4T1cells treated with a control siRNA (ctrl). **p<0.01 and non-significant (ns) were determined by one-way Anova analysis with Tukey’s test. Histograms and error bars represent the mean±SD of three independent experiments.

Fig. S5

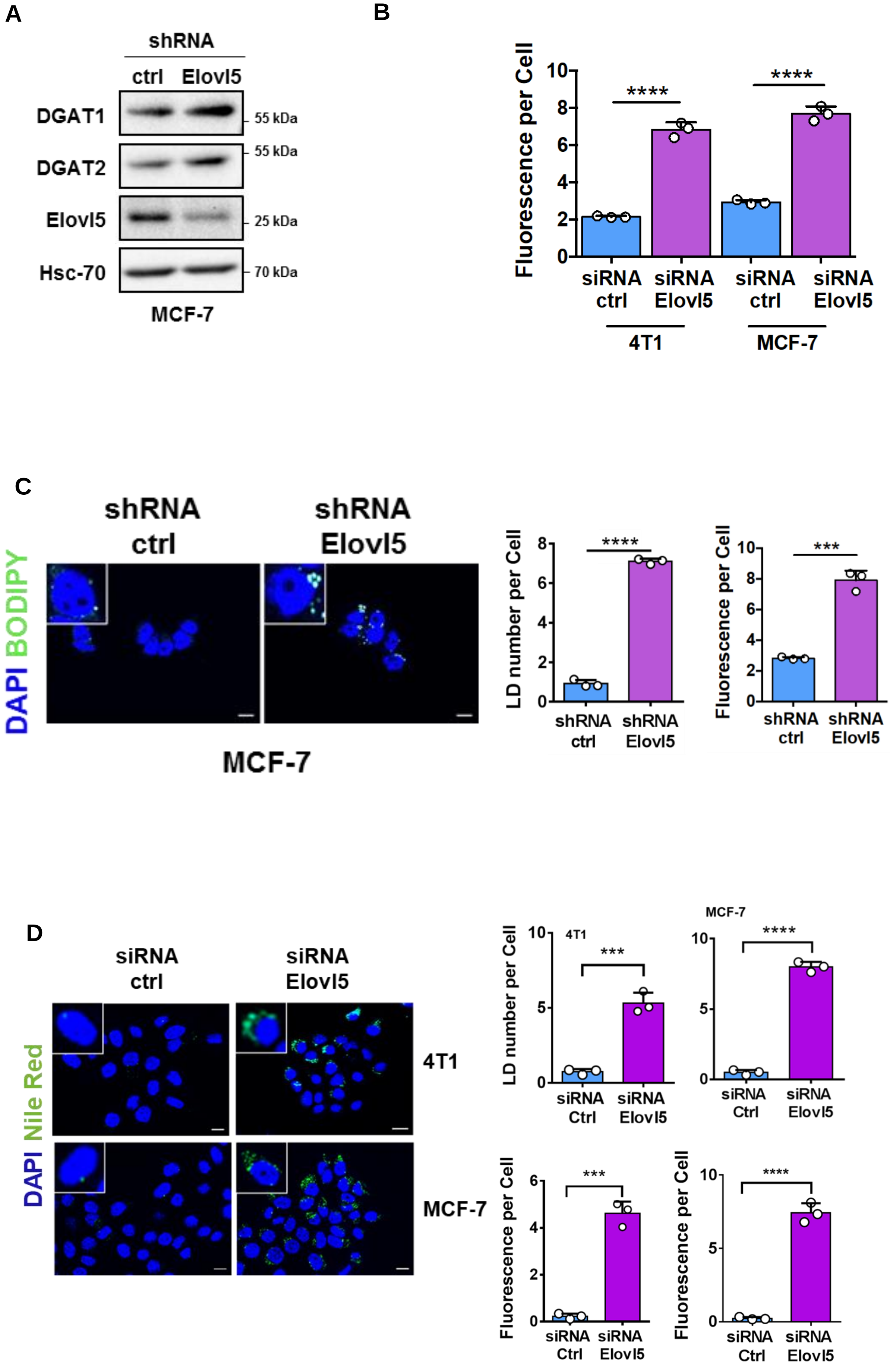
A
shRNA
ctrl
Elovl5
DGAT1
DGAT2
Elovl5
Hsc-70
55 kDa
55 kDa
25 kDa
70 kDa
MCF-7
B
Fluorescence per Cell
****
****
siRNA ctrl
siRNA Elovl5
siRNA ctrl
siRNA Elovl5
4T1
MCF-7
C
shRNA ctrl
shRNA Elovl5
DAPI BODIPY
MCF-7
LD number per Cell
Fluorescence per Cell
****
***
shRNA ctrl
shRNA Elovl5
D
siRNA ctrl
siRNA Elovl5
DAPI Nile Red
4T1
MCF-7
LD number per Cell
Fluorescence per Cell
***
****
siRNA Ctrl
siRNA Elovl5

Fig. S5

E

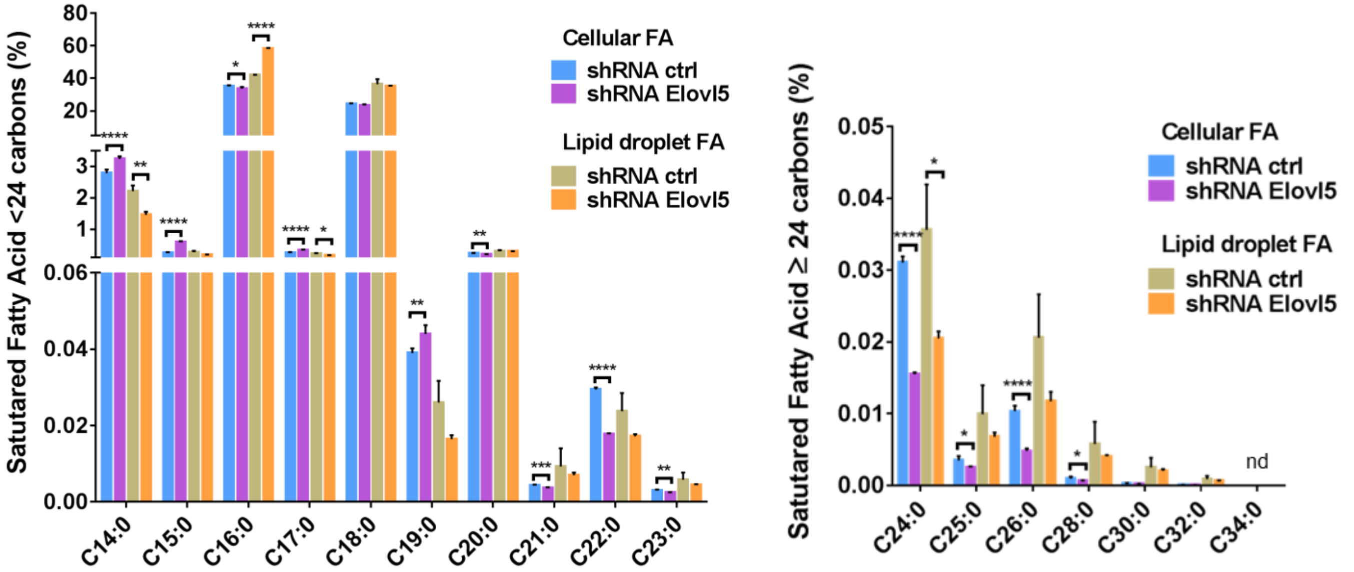
Satutared Fatty Acid <24 carbons (%)
Cellular FA
shRNA ctrl
shRNA Elovl5
Lipid droplet FA
shRNA ctrl
shRNA Elovl5
C14:0
C15:0
C16:0
C17:0
C18:0
C19:0
C20:0
C21:0
C22:0
C23:0
Satutared Fatty Acid ≥ 24 carbons (%)
Cellular FA
shRNA ctrl
shRNA Elovl5
Lipid droplet FA
shRNA ctrl
shRNA Elovl5
nd
C24:0
C25:0
C26:0
C28:0
C30:0
C32:0
C34:0

F

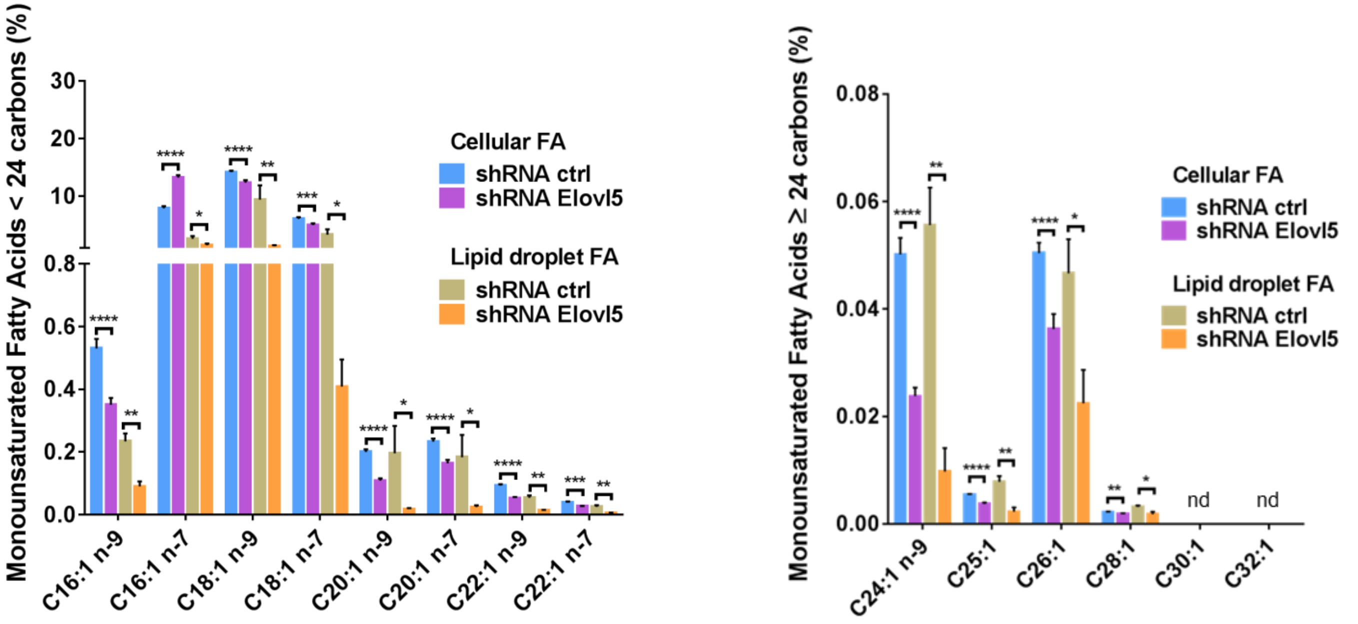
Monounsaturated Fatty Acids < 24 carbons (%)
Cellular FA
shRNA ctrl
shRNA Elovl5
Lipid droplet FA
shRNA ctrl
shRNA Elovl5
C16:1 n-9
C16:1 n-7
C18:1 n-9
C18:1 n-7
C20:1 n-9
C20:1 n-7
C22:1 n-9
C22:1 n-7
Monounsaturated Fatty Acids ≥ 24 carbons (%)
Cellular FA
shRNA ctrl
shRNA Elovl5
Lipid droplet FA
shRNA ctrl
shRNA Elovl5
nd
nd
C24:1 n-9
C25:1
C26:1
C28:1
C30:1
C32:1

G

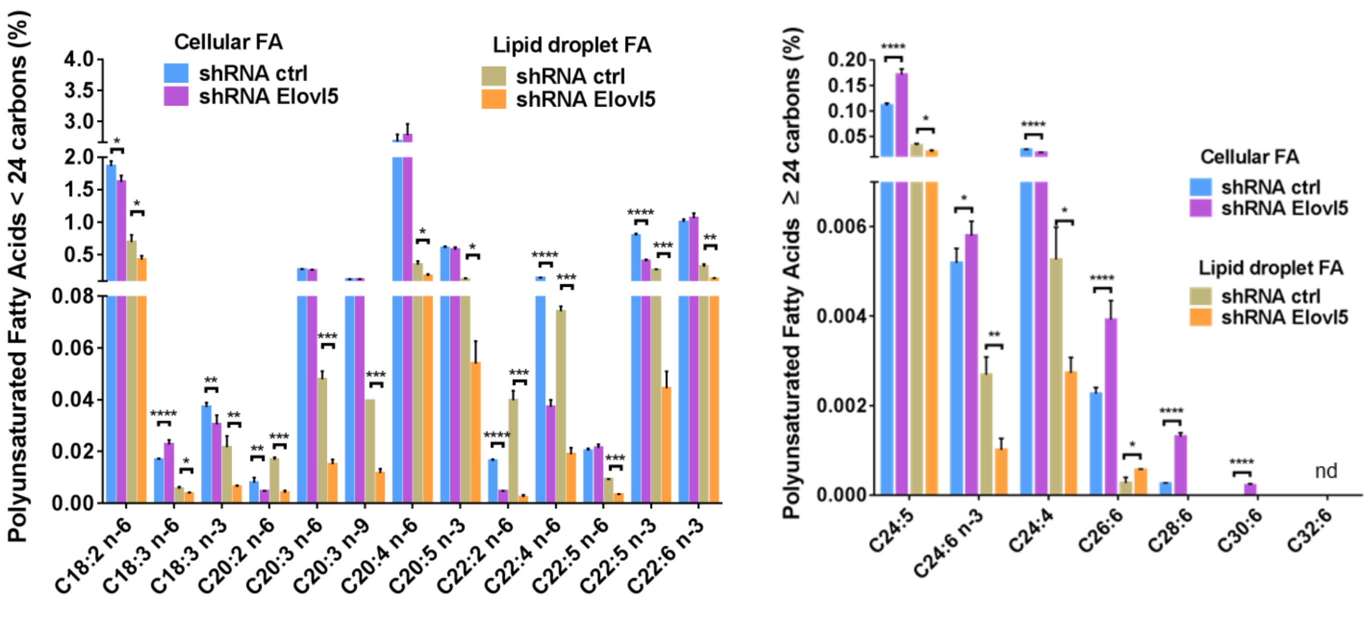
Polyunsaturated Fatty Acids < 24 carbons (%)
Cellular FA
shRNA ctrl
shRNA Elovl5
Lipid droplet FA
shRNA ctrl
shRNA Elovl5
C18:2 n-6
C18:3 n-6
C18:3 n-3
C20:2 n-6
C20:3 n-6
C20:3 n-9
C20:4 n-6
C20:5 n-3
C22:2 n-6
C22:4 n-6
C22:5 n-6
C22:5 n-3
C22:6 n-3
Polyunsaturated Fatty Acids ≥ 24 carbons (%)
Cellular FA
shRNA ctrl
shRNA Elovl5
Lipid droplet FA
shRNA ctrl
shRNA Elovl5
nd
C24:5
C24:6 n-3
C24:4
C26:6
C28:6
C30:6
C32:6

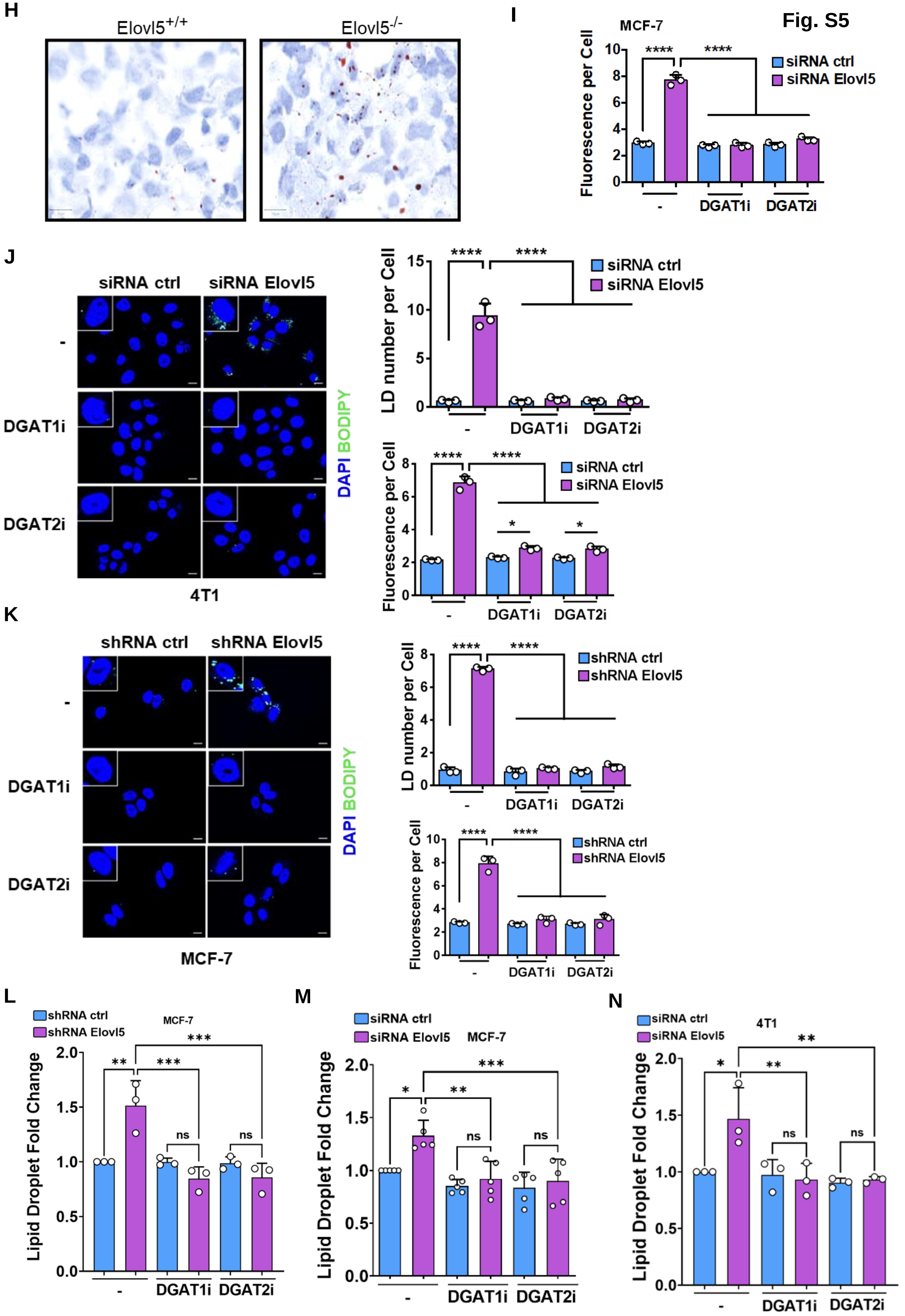
Fig. S5
H
Elovl5+/+
Elovl5-/-
I
MCF-7
Fluorescence per Cell
siRNA ctrl
siRNA Elovl5
****
-
DGAT1i
DGAT2i
J
siRNA ctrl
siRNA Elovl5
DAPI BODIPY
4T1
LD number per Cell
Fluorescence per Cell
*
K
shRNA ctrl
shRNA Elovl5
MCF-7
L
M
N
Lipid Droplet Fold Change
**
***
ns
4T1

Figure S5: Elovl5 downregulation increases lipid droplet abundance. A. Expression of DGAT1 and DGAT2 enzymes analyzed by western-blotting in MCF-7 cells with stable Elovl5 downregulation. B. Quantification of lipid droplet fluorescence intensity per cell in 4T1 and MCF-7 cells transfected with siRNA ctrl or against Elovl5 after Bodipy 493/503 staining (Images are shown in Fig. 5H). Data are indicated as the mean±SD of three independent experiments. ****p<0.0001 was determined by a Student's t test. C. Determination of lipid droplet content in stable Elovl5-depleted and control MCF-7 cells by fluorescence microscopy using a Bodipy 493/503 staining. The lipid droplet number was determined by assessing the number of total lipid droplet and the cell number (minimum 100 cells per experimental condition) in the field for the calculation of the average lipid droplet number per cell. In addition, a quantification of the average of fluorescence intensity per cell was performed in the randomly recorder fields using ImageJ software. Data are expressed as the mean±SD of three independent experiments. ***p<0.001 and ****p<0.0001 was determined by a Student's t test. Scale bar: 20 µm. D. Analysis of lipid droplet content in 4T1 and MCF-7 cells treated with control siRNA and Elovl5 siRNA. The nonpolar lipids of the lipid droplets were detected with a Nile Red staining by fluorescence microscopy. Data are expressed as the mean±SD of three independent experiments. ***p<0.001 and ****p<0.0001 was determined by a Student's t test. Scale bar: 20 µm. E-G. Percentage of total cellular or lipid droplet fatty acids in control and Elovl5-silenced MCF-7 cells. Shown are the mean±SD of three independent experiments with *p<0.05, **p<0.01, ***p<0.001 and ****p<0.0001 according to Student's t test. H. Representative images of Oil Red O staining of mammary tumors from MMTV-PyMT;Elovl5$^{+/+}$ (Elovl5$^{+/+}$) and MMTV-PyMT;Elovl5$^{-/-}$ (Elovl5$^{-/-}$) mice (Images correspond to the Figure 5J). Scale bar: 10 µm. I. Lipid droplet content in Elovl5-depleted MCF-7 with DGAT inhibitors or DMSO (control) treatment analyzed by Bodipy 493/503 staining and shown as the average of fluorescence intensity per cell (Images are shown in Figure

5K). Data are expressed as the mean±SD of three independent experiments with ****$p<0.0001$ according to Student's t test. J-K. Analysis of lipid droplet content by Bodipy 493/503 staining in MCF-7 cells with transient (J) and stable (K) Elovl5 depletion treated with or without DGAT inhibitors. Data are presented as average of LD number and fluorescence intensity per cell. Histograms and error bars represent the mean±SD of three independent experiments. ****$p<0.0001$ was determined by one-way Anova analysis with Tukey's test. L-N. Lipid droplet quantification by flow cytometry after Nile red staining in control or Elovl5-depleted MCF-7 (L-M) and 4T1 (N) cells. The detection of Nile Red fluorescence was performed with 488 nm excitation and 575 nm emission wavelengths. Data represent the mean±SD of 3-5 independent experiments with *$p<0.05$, **$p<0.01$ and non-significant (ns) according to One-way Anova analysis with Tukey's multiple comparison test.

Fig. S6

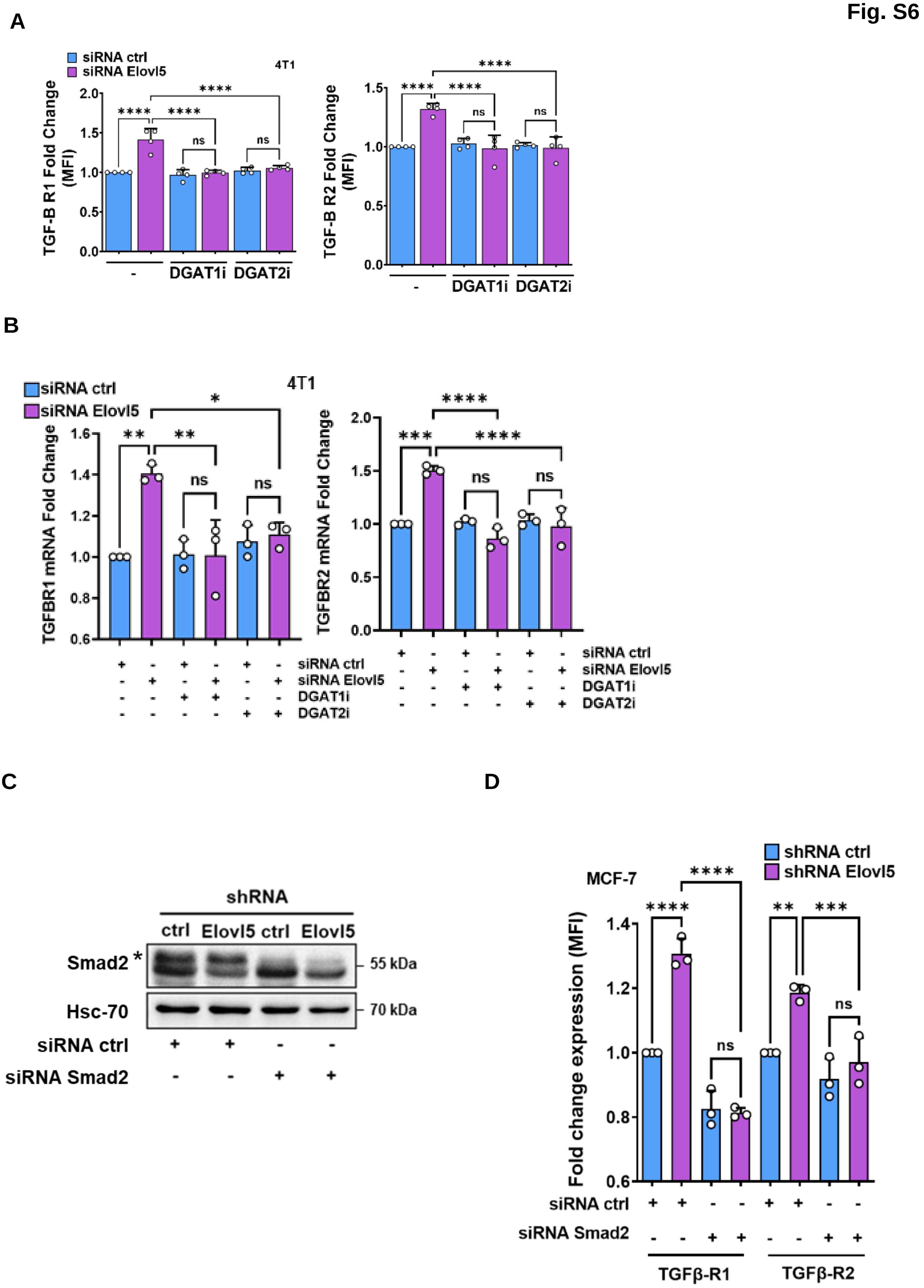

A
siRNA ctrl
siRNA Elovl5
4T1
TGF-B R1 Fold Change (MFI)
TGF-B R2 Fold Change (MFI)
DGAT1i
DGAT2i
B
siRNA ctrl
siRNA Elovl5
4T1
TGFBR1 mRNA Fold Change
TGFBR2 mRNA Fold Change
siRNA ctrl
siRNA Elovl5
DGAT1i
DGAT2i
C
shRNA
ctrl
Elovl5
ctrl
Elovl5
Smad2 *
Hsc-70
55 kDa
70 kDa
siRNA ctrl
siRNA Smad2
D
MCF-7
shRNA ctrl
shRNA Elovl5
Fold change expression (MFI)
siRNA ctrl
siRNA Smad2
TGFβ-R1
TGFβ-R2

Figure S6: Elovl5 dependent-LD accumulation controls expression of TGF-β receptors. A. Analysis of TGFβ-1 and 2 receptor expression by flow cytometry. The 4T1 cells with transient silencing of Elovl5 expression were treated for 48 hours with DGAT1 or 2 inhibitors. Values are represented as the fold change of mean of fluorescence intensity (MFI) relative to 4T1 cells treated with vehicle (DMSO) and a control shRNA or siRNA (ctrl). Data are expressed as the mean±SD of three independent experiments. ****$p<0,0001$ and non-significant (ns) determined by One-way Anova analysis with Tukey's test. B. Analysis of TGFR1 and 2 receptor mRNA expression in 4T1 cells by RT-qPCR. 4T1 cells transfected with siRNA were treated for 48 hours with DGAT1 and 2 inhibitors. Values are the fold change of Elovl5 mRNA expression relative to 4T1 cells treated with vehicle (DMSO) and a control siRNA (ctrl). Data are expressed as the mean±SD of three independent experiments. *$p<0.05$, **$p<0,01$, ***$p<0,001$, ****$p<0,0001$ and non-significant (ns) were determined by one-way Anova analysis with Tukey's test. C. Validation of Smad2 silencing by western-blotting in control and Elovl5 shRNA MCF-7 cells. D. Analysis of TGF-β receptor 1 and 2 expressions at 48 hours by flow cytometry in Elovl5-targeting shRNA and control shRNA MCF-7 cells treated by siRNA ctrl or against smad2. Values are the fold change in mean of fluorescence intensity (MFI) relative to shRNA control MCF-7 cells transfected with siRNA control. Histograms and error bars represent the mean±SD of three independent experiments with non-significant (ns), **$p<0.01$, ***$p<0.001$ and ****$p<0.0001$ according to one-way Anova analysis with Tukey's multiple comparison test.

Fig. S7

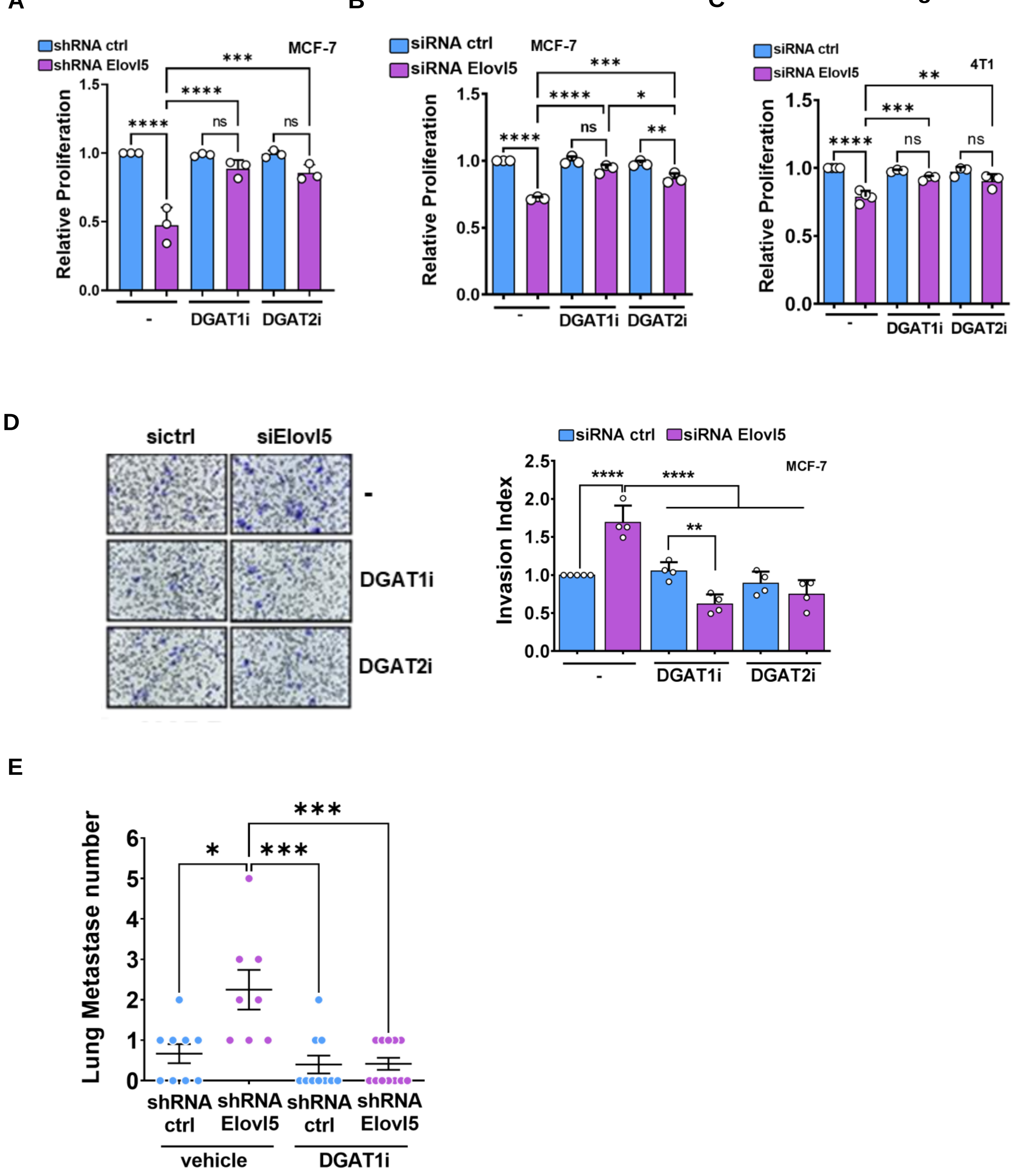
A
shRNA ctrl
shRNA Elovl5
MCF-7
Relative Proliferation
1.5
1.0
0.5
0.0
****
****
***
ns
ns
-
DGAT1i
DGAT2i
B
siRNA ctrl
siRNA Elovl5
MCF-7
Relative Proliferation
***
****
*
ns
**
-
DGAT1i
DGAT2i
C
siRNA ctrl
siRNA Elovl5
4T1
Relative Proliferation
**
***
****
ns
ns
-
DGAT1i
DGAT2i
D
sictrl
siElovl5
-
DGAT1i
DGAT2i
siRNA ctrl
siRNA Elovl5
MCF-7
Invasion Index
2.5
2.0
1.5
1.0
0.5
0.0
****
****
**
E
Lung Metastase number
6
5
4
3
2
1
0
*
***
***
shRNA ctrl
shRNA Elovl5
shRNA ctrl
shRNA Elovl5
vehicle
DGAT1i

Figure S7: Elovl5 dependent-LD accumulation controls proliferation, invasion and lung metastasis. A-C. Analysis of relative cell proliferation by crystal violet in MCF-7 (A-B) or 4T1 (C) cells with downregulation of Elovl5 and treated with DGAT1 or DGAT2 inhibitors. *$p<0,05$, **$p<0,01$, ***$p<0,001$, ****$p<0,0001$ and non-significant (ns) were determined by one-way Anova analysis with Tukey's test. Represented are the mean±SD of three independent experiments. D. Analysis of cell invasion through a Matrigel-coated membrane of Elovl5-depleted cancer MCF-7 cells and treated with DGAT inhibitors (DGAT1i and DGAT2i) relative to MCF-7 cells treated with a control siRNA (ctrl) and vehicle (DMSO). **$p<0,01$ and ****$p<0,0001$ were determined by One-way Anova analysis with Tukey's test. Histograms and error bars are the mean±SD of three independent experiments. Representative images are shown. E. Number of lung metastases in female NMRI-nude mice with tail vein injection of shRNA Elovl5 or shRNA control (ctrl) MCF-7 cells. DGAT1 inhibitor (10 µM) was loaded in MCF-7 cells 24 hours before injection. Lungs were collected 35-days post-injection. Data represent the mean±SEM with *$p<0.05$ and ***$p<0.001$ determined by Kruskall-Wallis analysis with a Dunn's multiple comparison test.
