# Supplementary material for: Downregulation of Elovl5 promotes breast cancer metastasis through a lipid-droplet accumulation-mediated induction of TGF-β receptors
Source: Cell Death Dis. 2022 Sep 2;13(9):758. doi: 10.1038/s41419-022-05209-6 (PMC9440092; doi:10.1038/s41419-022-05209-6)
Supplement: Supplementary file 3 — Table S2 [file 41419_2022_5209_MOESM3_ESM.docx]

Table S2: Subtypes of tumor breast tissues

| **Categories** | **Classification** | **Number of patients** |
| --- | --- | --- |
| **Gender** | Male | 0 |
|  | Female | 47 |
| **Age** | 30-50 | 8 |
|  | 51-70 | 21 |
|  | > 71 | 18 |
| **Subtypes** | TNBC | 10 |
|  | ER+/ HER2- | 28 |
|  | ER-/HER2+ | 9 |
| **Treatments** | Conservative surgery | 47 |
|  | Radiotherapy | 41 |
|  | Chemotherapy | 26 |
|  | Immunotherapy | 2 |
|  | Hormonotherapy | 31 |
| **TNM stage** | I | 20 |
|  | II | 19 |
|  | III | 8 |
|  | IV | 0 |
| **Lymph node invasion** | 0 (Negative) | 24 |
|  | 1 (Positive) | 23 |
| **Survival** | Alive | 46 |
|  | Death | 1 |
