# Supplementary material for: Downregulation of Elovl5 promotes breast cancer metastasis through a lipid-droplet accumulation-mediated induction of TGF-β receptors
Source: Cell Death Dis. 2022 Sep 2;13(9):758. doi: 10.1038/s41419-022-05209-6 (PMC9440092; doi:10.1038/s41419-022-05209-6)
Supplement: Supplementary file 5 — Table S4 [file 41419_2022_5209_MOESM5_ESM.docx]

Table S4: Fatty acid composition in control (shRNA ctrl) and Elovl5-silenced (shRNA Elovl5) MCF-7 cells. FA: fatty acids; SFA: saturated fatty acids; MUFA: monounsaturated fatty acids; PUFA: Polyunsaturated fatty acids.

|  | **Total Cellular FA** | | | **Total Lipid Droplet FA** | | |
| --- | --- | --- | --- | --- | --- | --- |
| % ±SD | shRNA ctrl | shRNA Elovl5 | p | shRNA ctrl | shRNA Elovl5 | p |
| SFA | 62,9 ±1,1 | 62,8 ±1,1 | ns | 81,6 ±3,6 | 95,7 ±0,6 | <0,001 |
| MUFA | 29,4 ±0,9 | 31,4 ±1,2 | <0,05 | 16,2 ±4 | 3,4 ±0,6 | <0,05 |
| PUFA | 7,7 ±0,3 | 7,1 ±0,5 | <0,05 | 2,2 ±0,4 | 0,9 ±0,08 | <0,05 |
| ≥ C24 MUFA | 0,10 ±0,005 | 0,06 ±0,004 | <0,0001 | 0,10 ±0,06 | 0,04 ±0,01 | <0,05 |
| ≥ C24 PUFA | 0,14 ±0,006 | 0,20  ±0,01 | <0,0001 | 0,04 ±0,006 | 0,02 ±0,004 | ns |
