# Supplementary material for: Downregulation of Elovl5 promotes breast cancer metastasis through a lipid-droplet accumulation-mediated induction of TGF-β receptors
Source: Cell Death Dis. 2022 Sep 2;13(9):758. doi: 10.1038/s41419-022-05209-6 (PMC9440092; doi:10.1038/s41419-022-05209-6)
Supplement: Supplementary file 6 — Table S5 [file 41419_2022_5209_MOESM6_ESM.docx]

Table S5: Primer sequences

| **Species** | **Gene name** | **Forward (5’-3’)** | **Reverse (5’-3’)** |
| --- | --- | --- | --- |
| **human** | Actine | AGCCTCGCCTTTGCCGA | CTGGTGCCTGGGGCG |
|  | ELOVL5 | TGAGGCAGTGGTCAAACAGGT | AGATATGTCATGAGTGGTTCCAAGA |
|  | Vimentin | GCAAAGATTCCACTTTGCGT | GAAATTGCAGGAGGAGATGC |
|  | CDH2 | TGTTTGACTATGAAGGCAGTGG | TCAGTCATCACCTCCACCAT |
|  | CDH1 | GAAAGCGGCTGATACTGACC | CGTACATGTCAGCCGCTTC |
|  | 18s | GTAACCCGTTGAACCCCATT | CCATCCAATCGGTAGTAGCG |
|  | Occludin | TTTGTGGGACAAGGAACACA | TCATTCACTTTGCCATTGGA |
|  | TGFβ1 | GAGCCTGAGGCCGACTACTA | TCGGAGCTCTGATGTGTTGA |
|  | TGFβ2 | AAGAAGCGTGCTTTGGATGCGG | ATGCTCCAGCACAGAAGTTGGC |
|  | TGFβ3 | ACTTGCACCACCTTGGACTTC | GGTCATCACCGTTGGCTCA |
|  | TGFβ1R | GCAGAGCTGTGAAGCCTTGAGA | TGCCTTCCTGTTGACTGAGTT |
|  | TGFβ2R | ATGACATCTCGCTGTAATGC | GGATGCCCTGGTGGTTGA |
| **mouse** | Actine | ATGGAGGGGAATACAGCCC | TTCTTTGCAGCTCCTTCGTT |
|  | Ncadherin | ATGTGCCGGATAGCGGGAGC | TACACCGTGCCGTCCTCGTC |
|  | Vimentin | CTTGAACGGAAAGTGGAATCCT | GTCAGGCTTGGAAACGTCC |
|  | Ecadherin | AACCCAAGCACGTATCAGGG | GAGTGTTGGGGGCATCATCA |
|  | ELOVL5 | TTCGATGCGTCACTCAGTACC | TGTCCAGGAGGAACCATCCTT |
|  | 18s | GTAACCCGTTGAACCCCATT | CCATCCAATCGGTAGTAGCG |
|  | Occludin | CCACCCCCATCTGACTATGC | TCGCTTGCCATTCACTTTGC |
|  | TGFβ1 | CCCAGTCTCCATACATTAACCC | CACCATCACCTTGAACTCTGAC |
|  | TGFβ2 | TTGTTGCCCTCCTACAGACTGG | GTAAAGAGGGCGAAGGCAGCAA |
|  | TGFβ3 | CCTGGCCCTGCTGAACTTG | TTGATGTGGCCGAAGTCCAAC |
|  | TGFβ1R | TCTGCATTGCACTTATGCTGA | AAAGGGCGATCTAGTGATGGA |
|  | TGFβ2R | TTGGATTGCCAGTGCTAACCC | AACAAGCCACAGTAACATGACA |
| **PyMT Genotyping** | internal control | CAAATGTTGCTTGTCTGGTG | GTCAGTCGAGTGCACAGTTT |
|  | PyMT transgene | GGA AGC AAG TAC TTC ACA AGG G | GGA AAG TCA CTA GGA GCA GGG |
| **Elovl5 Genotyping** | O Reverse |  | GGCTAGACACACCTAGCAGAGC |
|  | K Reverse |  | TGCGGTACCAGACTCTCCCAT |
|  | C' Forward | AGAAGACAGACCTGGTTTGGCATA |  |
