# Supplementary material for: Downregulation of Elovl5 promotes breast cancer metastasis through a lipid-droplet accumulation-mediated induction of TGF-β receptors
Source: Cell Death Dis. 2022 Sep 2;13(9):758. doi: 10.1038/s41419-022-05209-6 (PMC9440092; doi:10.1038/s41419-022-05209-6)
Supplement: Supplementary file 7 — Table S6 [file 41419_2022_5209_MOESM7_ESM.docx]

Table S6

| **Application** | **Targeted protein** | **Reference** | **Producer** | **Dilution** |
| --- | --- | --- | --- | --- |
| **Western blot** | DGAT1 | A6857 | AB clonal | 1/1000 |
|  | DGAT2 | A13890 | AB clonal |  |
|  | ELOVL5 | HPA047752 | Sigma-Aldrich |  |
|  | E-cadherin | 24E10-3195S | Cell Signaling Technologies |  |
|  | Hsc70 | sc-7298 | Santa Cruz Biotechnology |  |
|  | N-cadherin | ab18203 | Abcam |  |
|  | Occludin | GTX114949 | Genetex |  |
|  | Phospho ACC | s79-3661P | Cell Signaling Technologies |  |
|  | Total ACC | C83B10-3676P | Cell Signaling Technologies |  |
|  | p-smad 2/3 | D27F4-8828 | Cell Signaling Technologies |  |
|  | Total smad 2/3 | D7G7-8685 | Cell Signaling Technologies |  |
|  | Vimentin | D21H3-5741 | Cell Signaling Technologies |  |
|  | SCD1 | CDE10-sc58420 | Santa Cruz Biotechnology | 1/400 |
|  | TGFβ R2 | MAB532 | R&D Systems | 1/500 |
|  | β-actin | sc-47778 | Santa Cruz Biotechnology | 1/2500 |
| **Immunofluorescence** | TGFβ R1 | PA5-38718 | Invitrogen | 1/250 |
| **Flow cytometry** | human TGFBR2-PE | W170555 | BioLegend | 1/100 |
|  | IgG control-PE | RTK4530 | BioLegend |  |
|  | mouse TGFBR2-PE | FAB532P | R&D Systems |  |
|  | TGFβ R1 | ABF17-I | EMD Millipore |  |
