# Supplementary material for: Downregulation of Elovl5 promotes breast cancer metastasis through a lipid-droplet accumulation-mediated induction of TGF-β receptors
Source: Cell Death Dis. 2022 Sep 2;13(9):758. doi: 10.1038/s41419-022-05209-6 (PMC9440092; doi:10.1038/s41419-022-05209-6)
Supplement: Supplementary file 8 — Supplementary Materials and methods [file 41419_2022_5209_MOESM8_ESM.docx]

**Supplementary Materials and methods**

**Cell death assay**

Total cellular populations (adherent and floating cells ) were collected and centrifuged. The cells were incubated with 2 µl of 7-AAD (MiltenyiBiotec) for 8 minutes in RT. The staining cells were detected on LSR Fortessa cytometer and BD FACSDiva software (BD Biosciences). Data were analyzed using the FlowJo software (Tree Star, USA) and presented in percentage of positive cells. The data was obtained from three independent experiments.

**Cell cycle analysis**

4T1 and MCF7 cells were collected and fixed with 70% Ethanol overnight at -20^o^C. The cells were then stained with propidium iodide (40µg/ml) (#ICT-638; ImmunoChemistry) in presence of Ribonuclease A DNA-free (5µg/ml) (#EN0531; ThermoFisher Scientific). Single PI-positive cell was analyzed with BD FACSCanto (BD biosciences). Cell populations from each phase was determined by ModiFIT software presented in percentage of cells in the corresponding cell cycle phase. The data was obtained from three independent experiments.

**Nile Red and Bodipy 493/503 staining**

Cells on coverslips were fixed with 4% paraformaldehyde for 10 minutes at RT and incubated with 1 μM Nile Red (Sigma-Aldrich) or 10 μM of Bodipy 493/503 (Thermo Fisher Scientific) solution for 30 minutes. After washing, coverslips were mounted with ProlongTM diamond antifade mountant (Molecular Probes) containing 20 µM Hoechst (Thermo Fisher Scientific). The detection of neutral lipids using Nile Red or Bodipy 493/503 was carried out using 470 nm excitation and 525 nm emission wavelengths. The staining was observed with Axio Imager 2 (Carl Zeiss Microscopy GmbH, Jena, Germany) connected to an Apotome 2 module (Carl Zeiss GmbH). Images were taken with a AxioCam MRm monochrome CCD camera (Carl Zeiss GmbH). The lipid droplet number was determined by assessing the number of total lipid droplets and the cell number (minimum 100 cells per experimental condition) in the field for the calculation of the average lipid droplet number per cell. In addition, a quantification of the average of fluorescence intensity per cell was performed in the randomly recorded fields using imageJ software.

For flow cytometry analysis of Nile Red staining, the cells were incubated for 15 minutes with 1µM Nile Red solution (Sigma-Aldrich) at room temperature. Cells were washed and collected in PBS for analysis using an LSR Fortessa cytometer (excitation 488 nm and emission 575 nm) and the BD FACSDiva software (BD Biosciences). Gating strategies were designed to exclude negative staining, cellular debris and duplexes. Data were analyzed using the FlowJo software (Tree Star, USA) and presented as a mean of fluorescence intensity.
