# Supplementary material for: Downregulation of Elovl5 promotes breast cancer metastasis through a lipid-droplet accumulation-mediated induction of TGF-β receptors
Source: Cell Death Dis. 2022 Sep 2;13(9):758. doi: 10.1038/s41419-022-05209-6 (PMC9440092; doi:10.1038/s41419-022-05209-6)
Supplement: Supplementary file 9 — Original western-blots [file 41419_2022_5209_MOESM9_ESM.pdf]

Figure S1

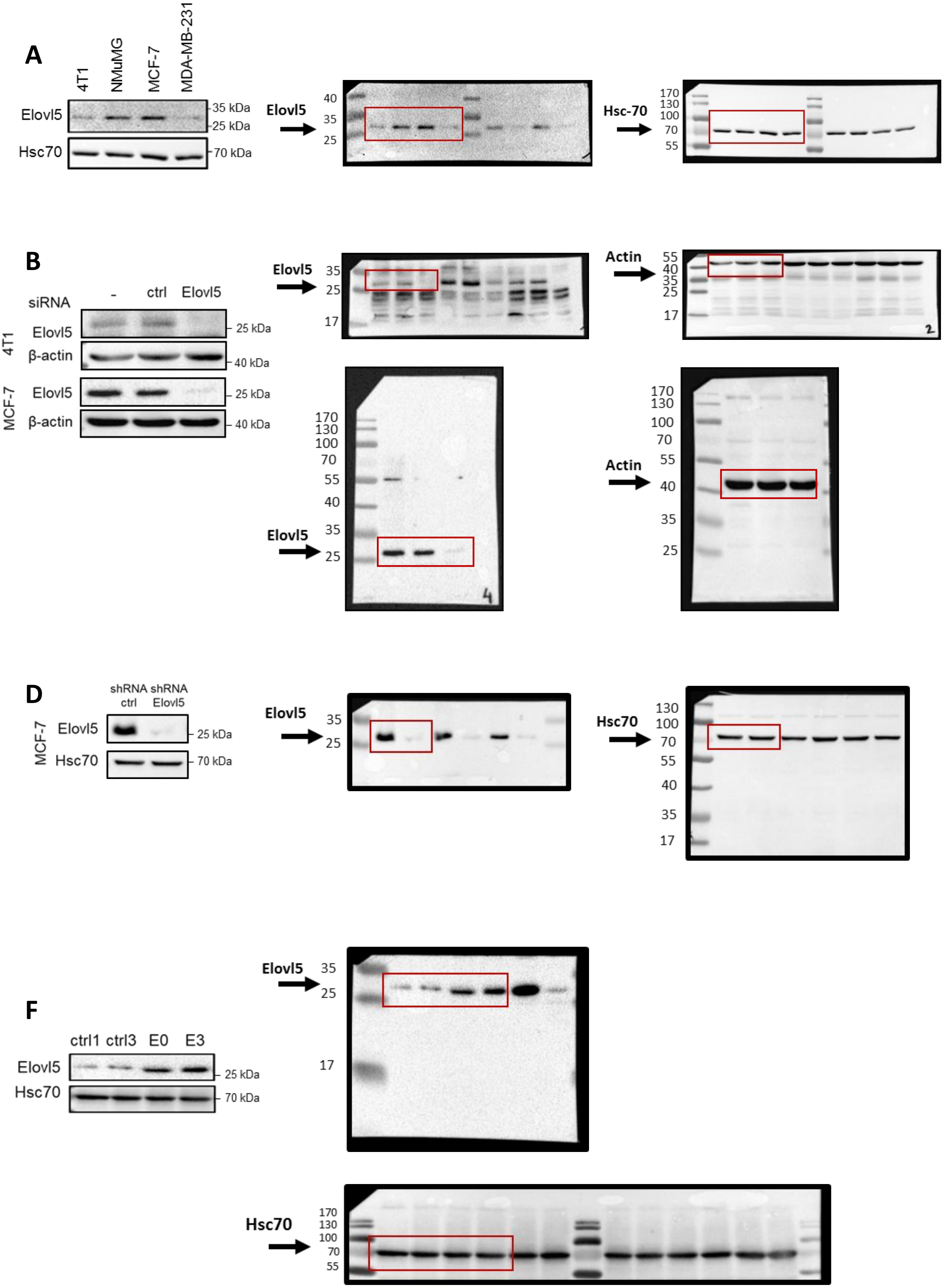

A
4T1
NMuMG
MCF-7
MDA-MB-231
Elovl5
Hsc70
35 kDa
25 kDa
70 kDa
Elovl5
40
35
25
Hsc-70
170
130
100
70
55
B
siRNA
-
ctrl
Elovl5
4T1
MCF-7
Elovl5
β-actin
25 kDa
40 kDa
Elovl5
35
25
17
Actin
55
40
35
25
17
170
130
100
70
55
40
35
25
Elovl5
Actin
D
shRNA ctrl
shRNA Elovl5
MCF-7
Elovl5
Hsc70
25 kDa
70 kDa
Elovl5
35
25
Hsc70
130
100
70
55
40
35
17
F
ctrl1
ctrl3
E0
E3
Elovl5
Hsc70
25 kDa
70 kDa
Elovl5
35
25
17
Hsc70
170
130
100
70
55

Figure 3

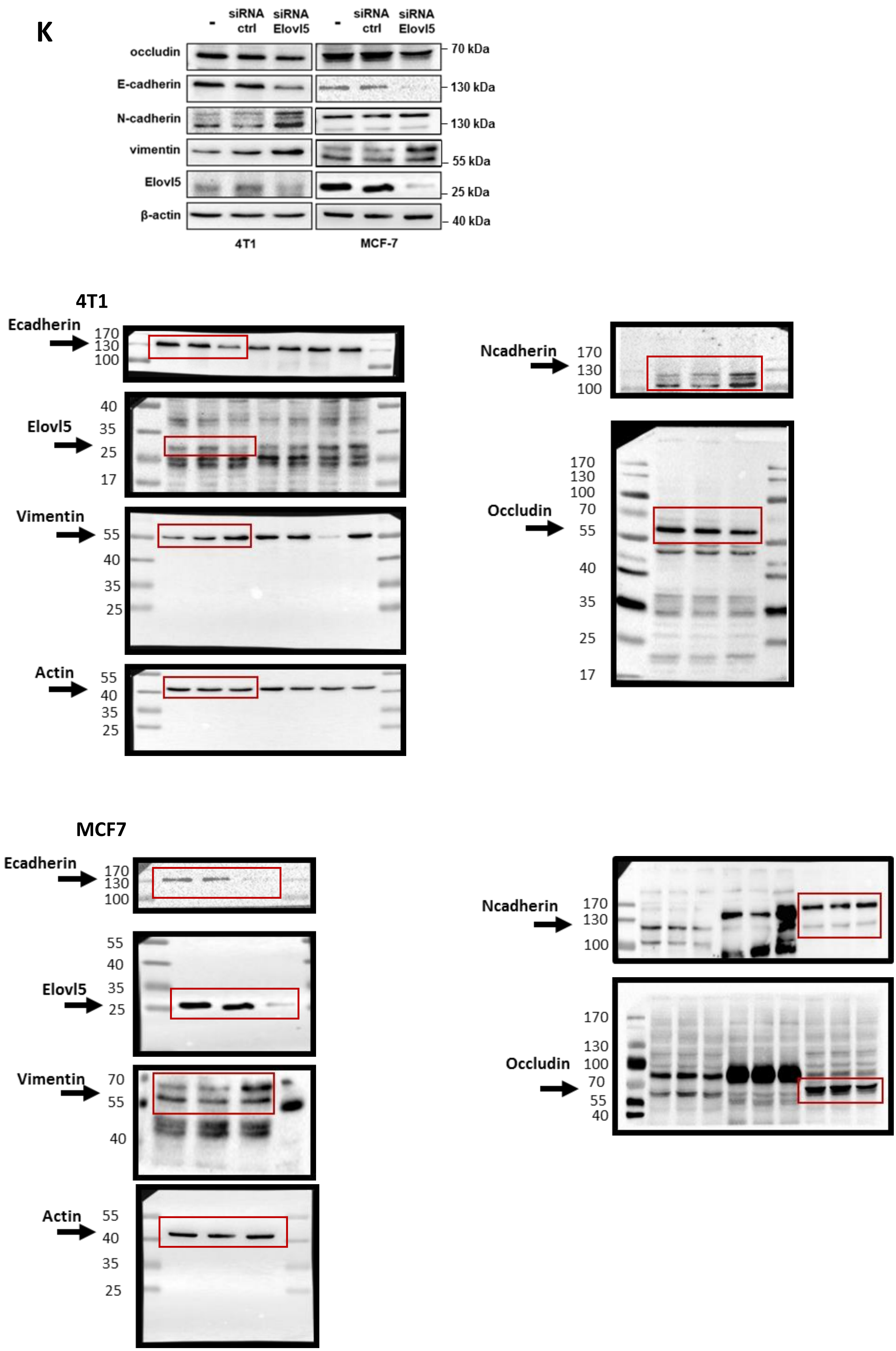
K
-
siRNA ctrl
siRNA Elovl5
-
siRNA ctrl
siRNA Elovl5
occludin
E-cadherin
N-cadherin
vimentin
Elovl5
β-actin
70 kDa
130 kDa
130 kDa
55 kDa
25 kDa
40 kDa
4T1
MCF-7
4T1
Ecadherin
170
130
100
Elovl5
40
35
25
17
Vimentin
55
40
35
25
Actin
55
40
35
25
Ncadherin
170
130
100
Occludin
170
130
100
70
55
40
35
25
17
MCF7
Ecadherin
170
130
100
Elovl5
55
40
35
25
Vimentin
70
55
40
Actin
55
40
35
25
Ncadherin
170
130
100
Occludin
170
130
100
70
55
40

Figure 3

L

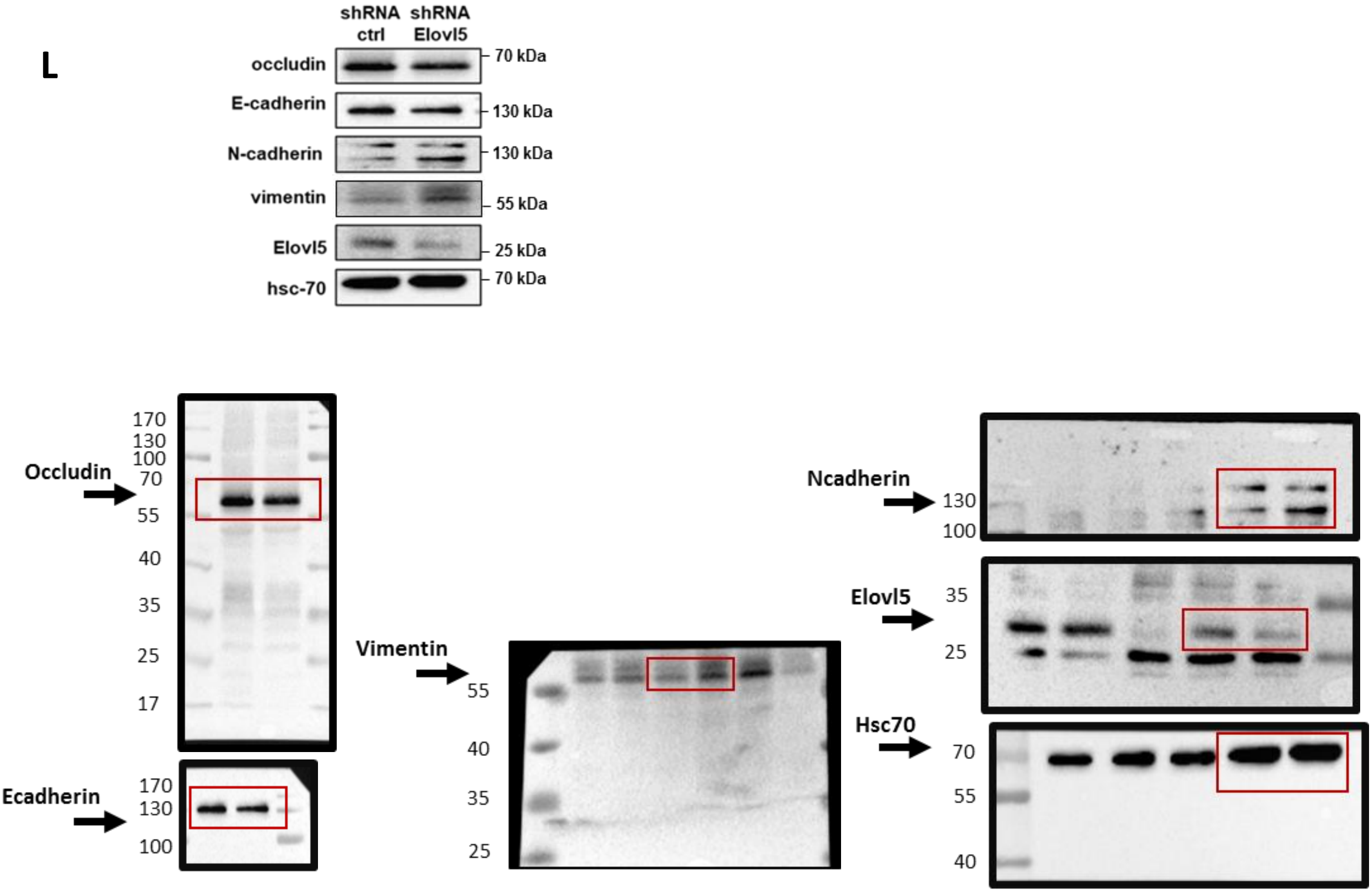
shRNA ctrl
shRNA Elovl5
occludin
E-cadherin
N-cadherin
vimentin
Elovl5
hsc-70
70 kDa
130 kDa
130 kDa
55 kDa
25 kDa
70 kDa
Occludin
170
130
100
70
55
40
35
25
17
Ecadherin
170
130
100
Vimentin
55
40
35
25
Ncadherin
130
100
Elovl5
35
25
Hsc70
70
55
40

M

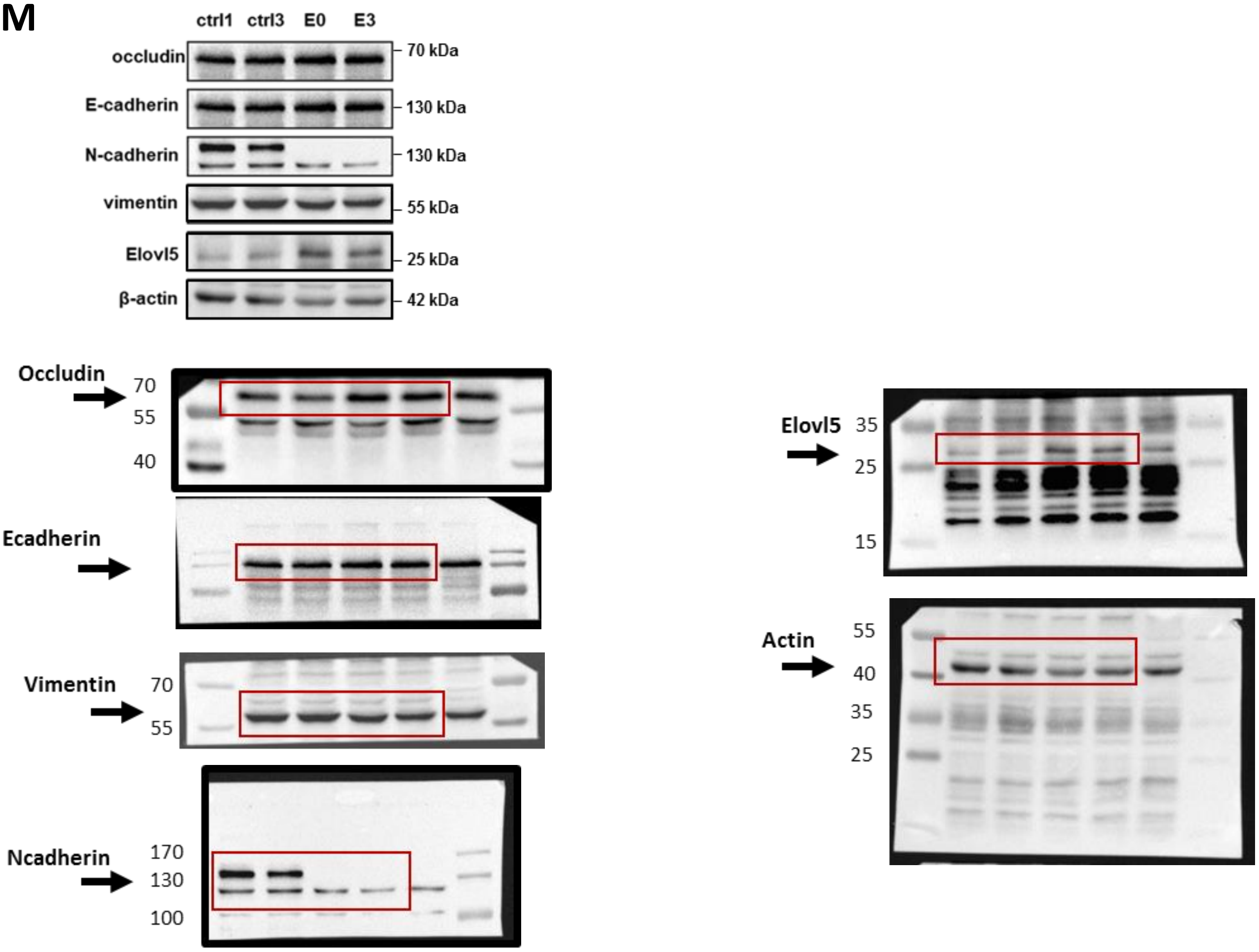
ctrl1
ctrl3
E0
E3
occludin
E-cadherin
N-cadherin
vimentin
Elovl5
β-actin
70 kDa
130 kDa
130 kDa
55 kDa
25 kDa
42 kDa
Occludin
70
55
40
Ecadherin
Vimentin
70
55
Ncadherin
170
130
100
Elovl5
35
25
15
Actin
55
40
35
25

Figure 4

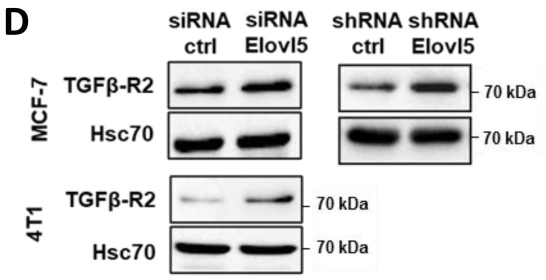
D
siRNA ctrl
siRNA Elovl5
shRNA ctrl
shRNA Elovl5
MCF-7
TGFβ-R2
Hsc70
70 kDa
70 kDa
4T1
TGFβ-R2
Hsc70
70 kDa
70 kDa

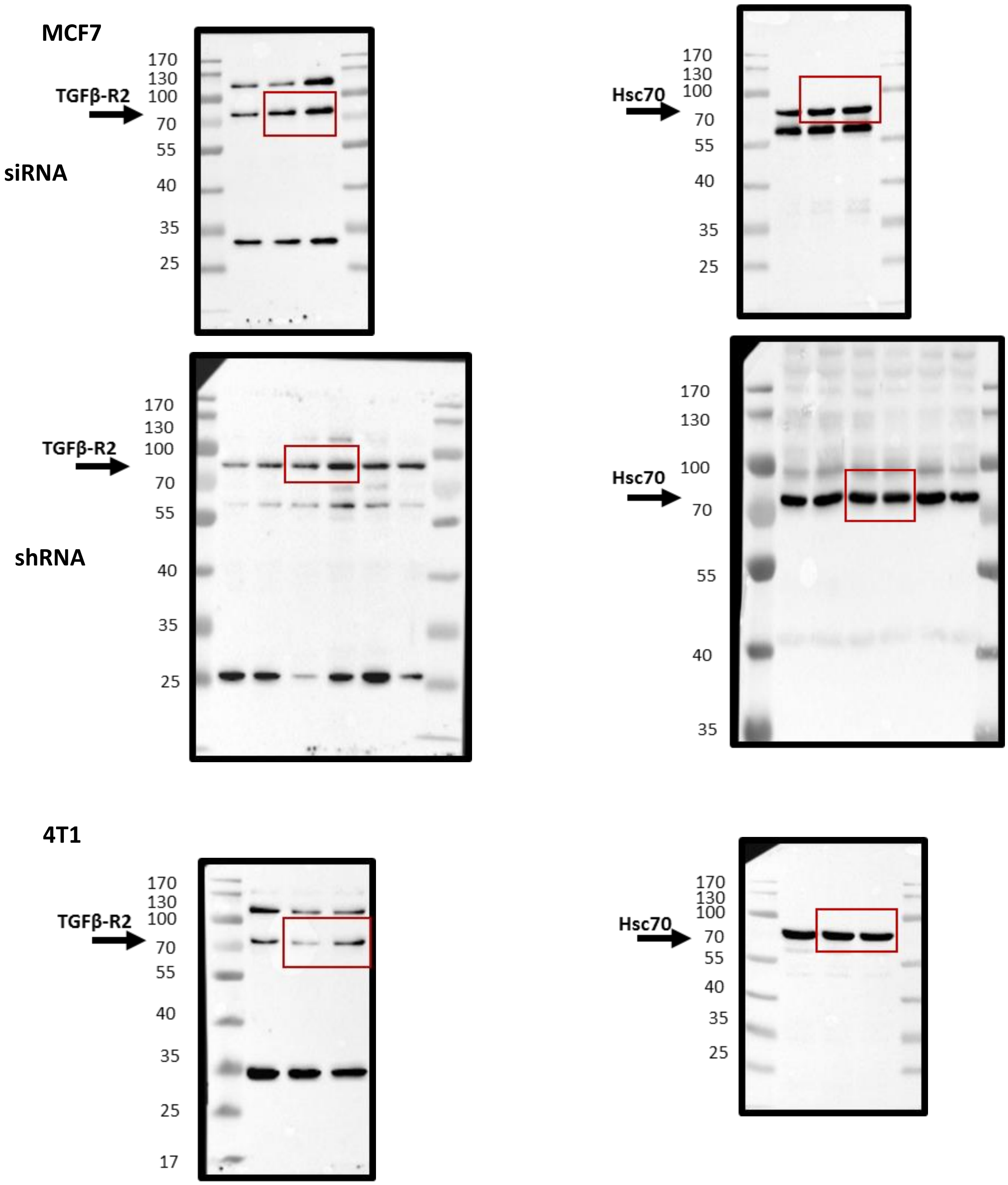
MCF7
siRNA
TGFβ-R2
170
130
100
70
55
40
35
25
Hsc70
170
130
100
70
55
40
35
25
shRNA
TGFβ-R2
170
130
100
70
55
40
35
25
Hsc70
170
130
100
70
55
40
35
4T1
TGFβ-R2
170
130
100
70
55
40
35
25
17
Hsc70
170
130
100
70
55
40
35
25

Figure 4

G

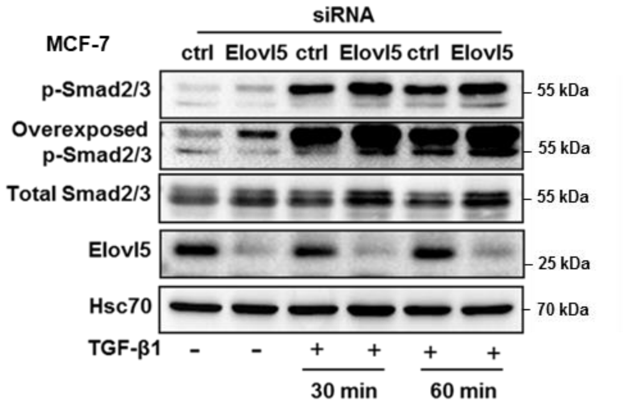
siRNA
MCF-7
ctrl Elovl5 ctrl Elovl5 ctrl Elovl5
p-Smad2/3
Overexposed p-Smad2/3
Total Smad2/3
Elovl5
Hsc70
55 kDa
55 kDa
55 kDa
25 kDa
70 kDa
TGF-β1 - - + + + +
30 min
60 min

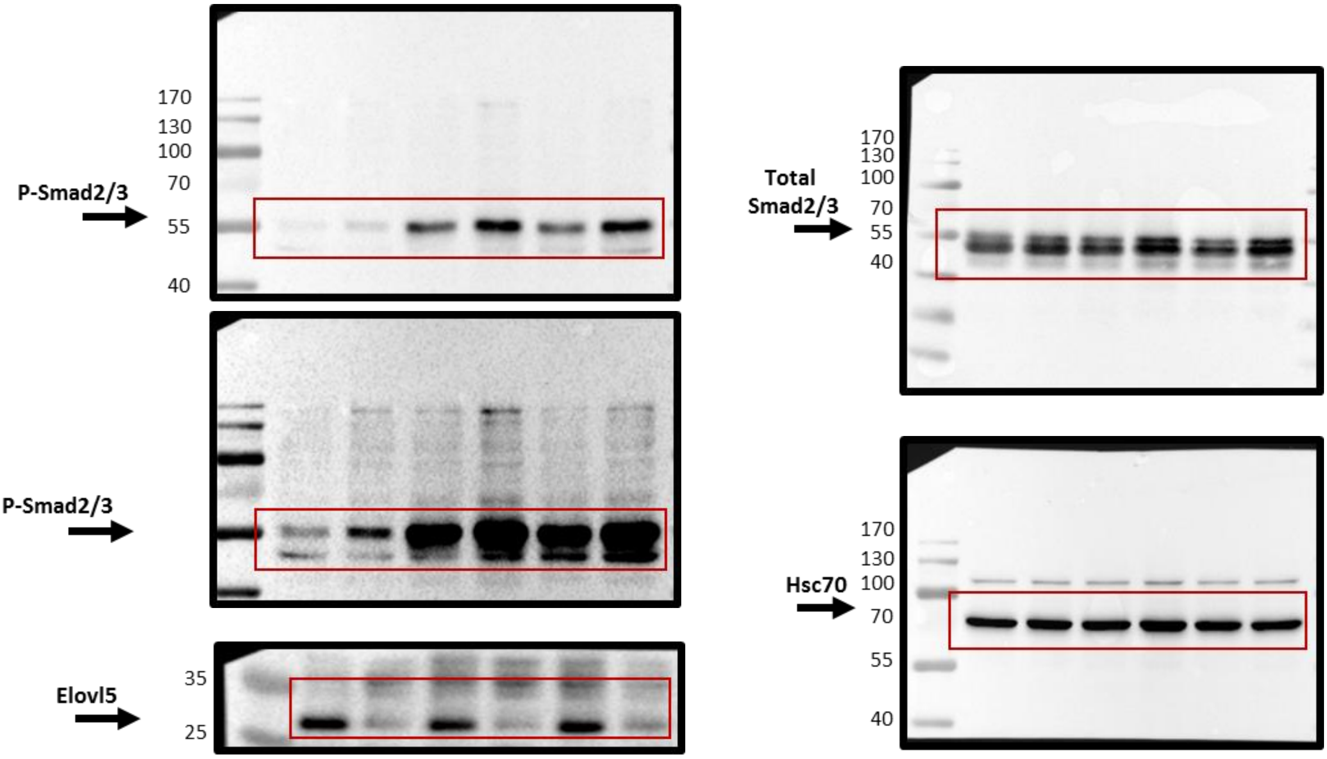
P-Smad2/3
170
130
100
70
55
40
P-Smad2/3
Elovl5
35
25
Total Smad2/3
170
130
100
70
55
40
Hsc70
170
130
100
70
55
40

Figure S4

E

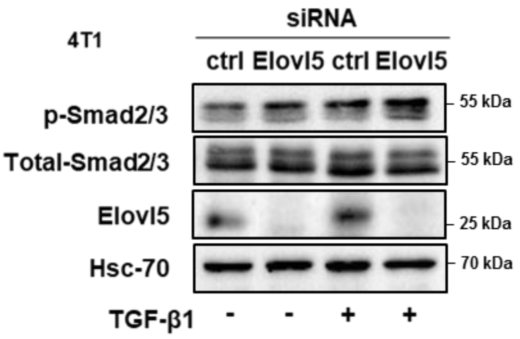
4T1
siRNA
ctrl Elovl5 ctrl Elovl5
p-Smad2/3
Total-Smad2/3
Elovl5
Hsc-70
55 kDa
55 kDa
25 kDa
70 kDa
TGF-β1 - - + +

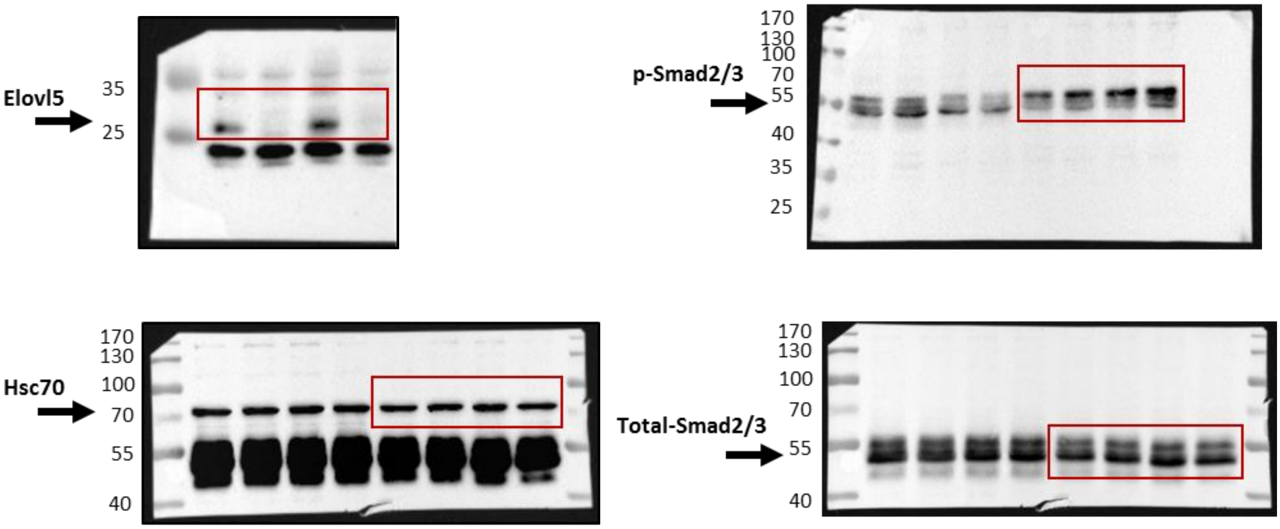
Elovl5
35
25
p-Smad2/3
170
130
100
70
55
40
35
25
Hsc70
170
130
100
70
55
40
Total-Smad2/3
170
130
100
70
55
40

Figure 5

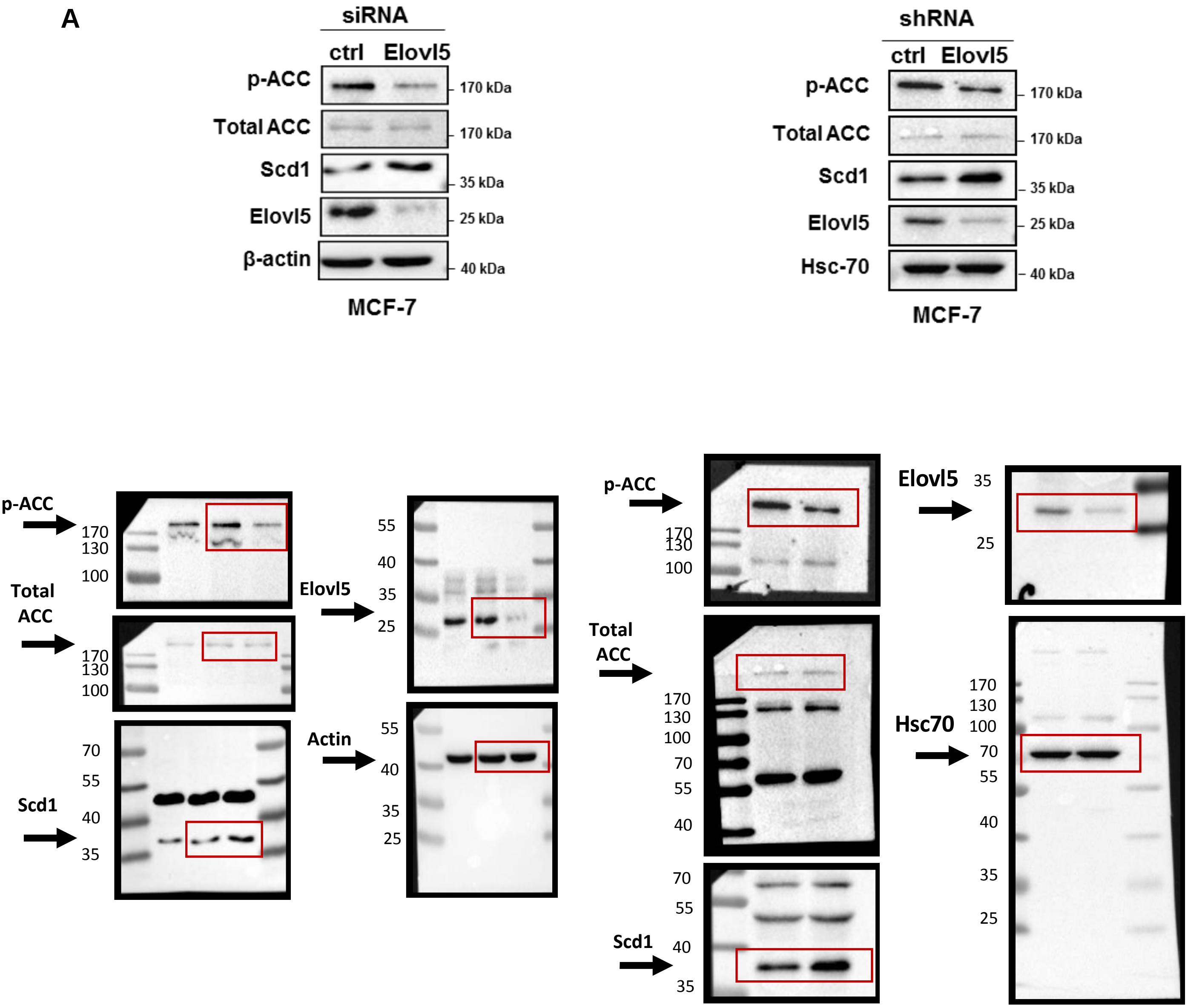
A
siRNA
ctrl
Elovl5
p-ACC
170 kDa
Total ACC
170 kDa
Scd1
35 kDa
Elovl5
25 kDa
β-actin
40 kDa
MCF-7
shRNA
ctrl
Elovl5
p-ACC
170 kDa
Total ACC
170 kDa
Scd1
35 kDa
Elovl5
25 kDa
Hsc-70
40 kDa
MCF-7
p-ACC
170
130
100
Total ACC
170
130
100
70
55
Scd1
40
35
55
40
Elovl5
35
25
Actin
55
40
35
25
p-ACC
170
130
100
Total ACC
170
130
100
70
55
40
70
55
Scd1
40
35
Elovl5
35
25
Hsc70
170
130
100
70
55
40
35
25

Figure 5

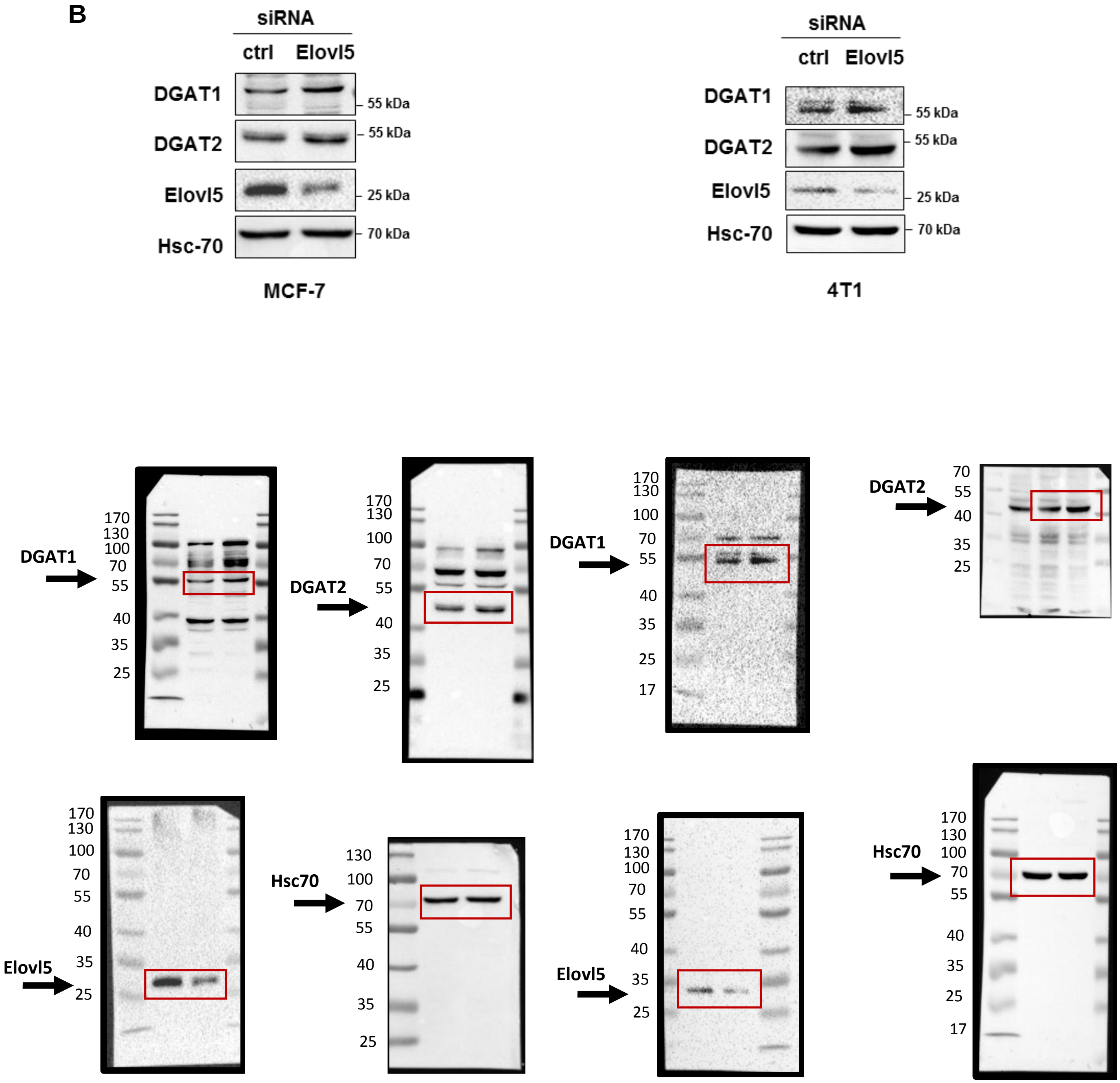

B
siRNA
ctrl
Elovl5
DGAT1
DGAT2
Elovl5
Hsc-70
55 kDa
55 kDa
25 kDa
70 kDa
MCF-7
4T1
DGAT1
DGAT2
Elovl5
Hsc70
170
130
100
70
55
40
35
25
17

Figure S5

A

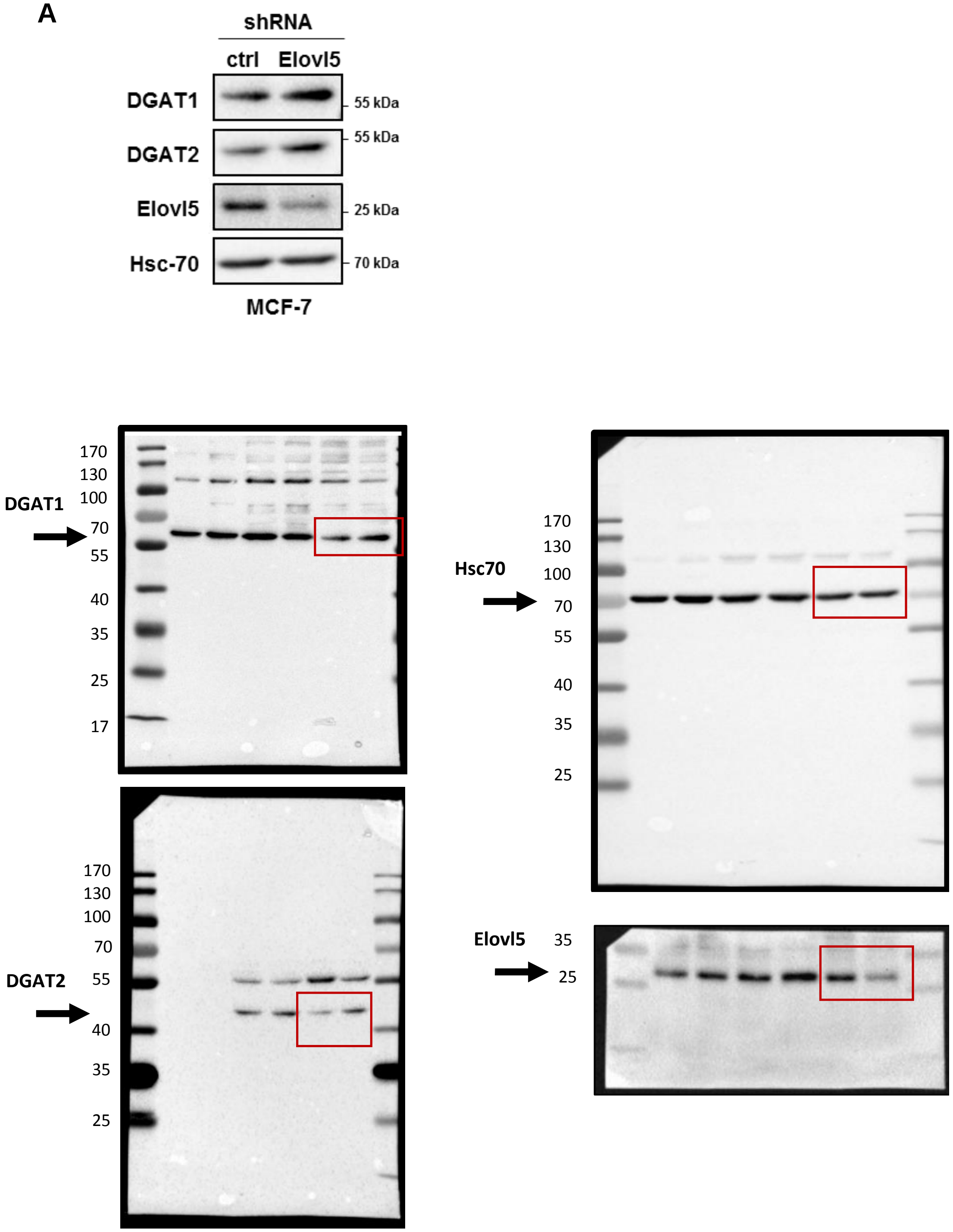

shRNA
ctrl
Elovl5
DGAT1
DGAT2
Elovl5
Hsc-70
55 kDa
55 kDa
25 kDa
70 kDa
MCF-7
DGAT1
170
130
100
70
55
40
35
25
17
Hsc70
170
130
100
70
55
40
35
25
DGAT2
170
130
100
70
55
40
35
25
Elovl5
35
25

Figure 6

F

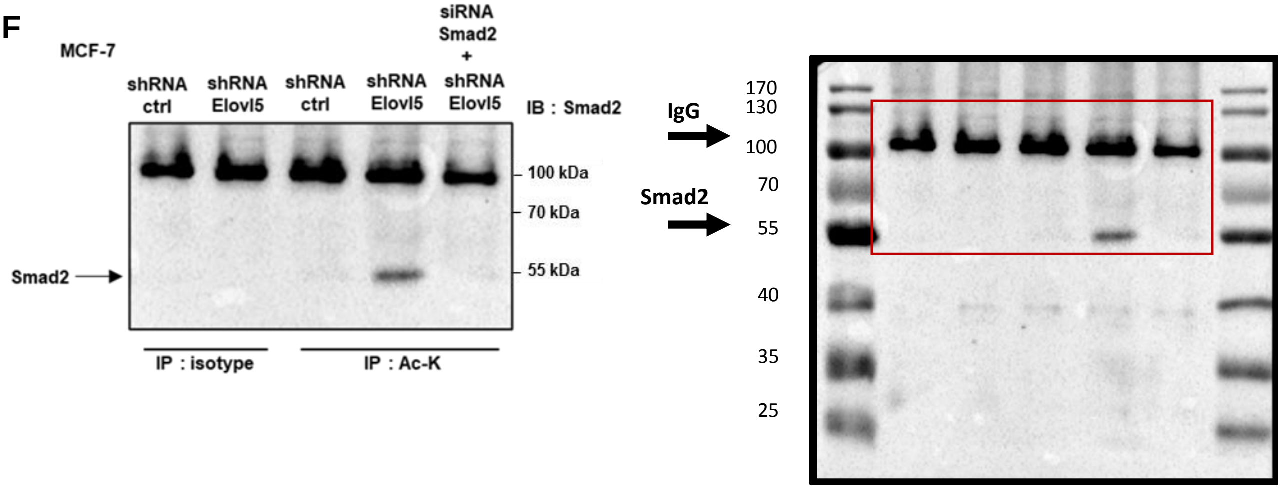
MCF-7
shRNA ctrl
shRNA Elovl5
shRNA ctrl
shRNA Elovl5
siRNA Smad2 + shRNA Elovl5
IB : Smad2
100 kDa
70 kDa
55 kDa
Smad2
IP : isotype
IP : Ac-K
IgG
Smad2
170
130
100
70
55
40
35
25

G

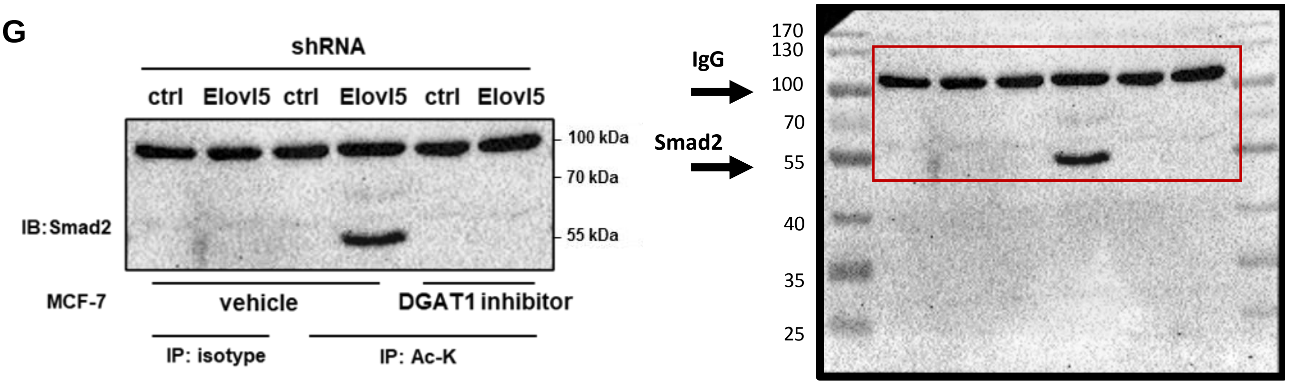
shRNA
ctrl
Elovl5
ctrl
Elovl5
ctrl
Elovl5
100 kDa
70 kDa
55 kDa
IB:Smad2
MCF-7
vehicle
DGAT1 inhibitor
IP: isotype
IP: Ac-K
IgG
Smad2
170
130
100
70
55
40
35
25

Figure S6

C

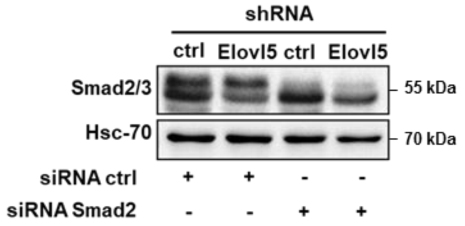
shRNA
ctrl
Elovl5
ctrl
Elovl5
Smad2/3
55 kDa
Hsc-70
70 kDa
siRNA ctrl
+ + - -
siRNA Smad2
- - + +

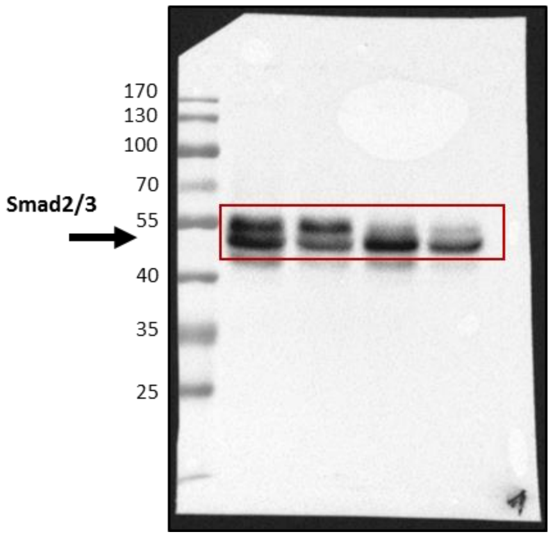
170
130
100
70
Smad2/3
55
40
35
25

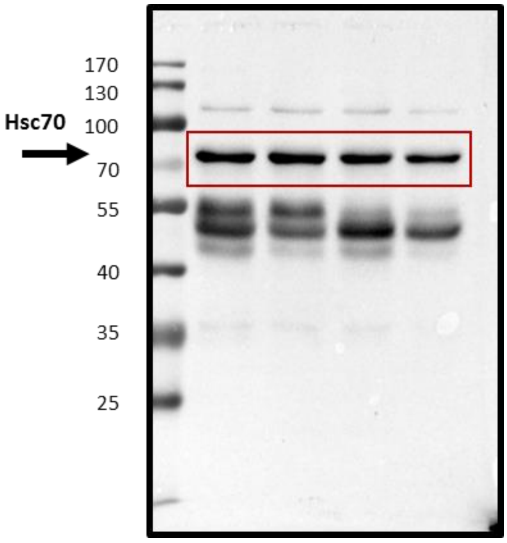
170
130
Hsc70
100
70
55
40
35
25

Figure 7

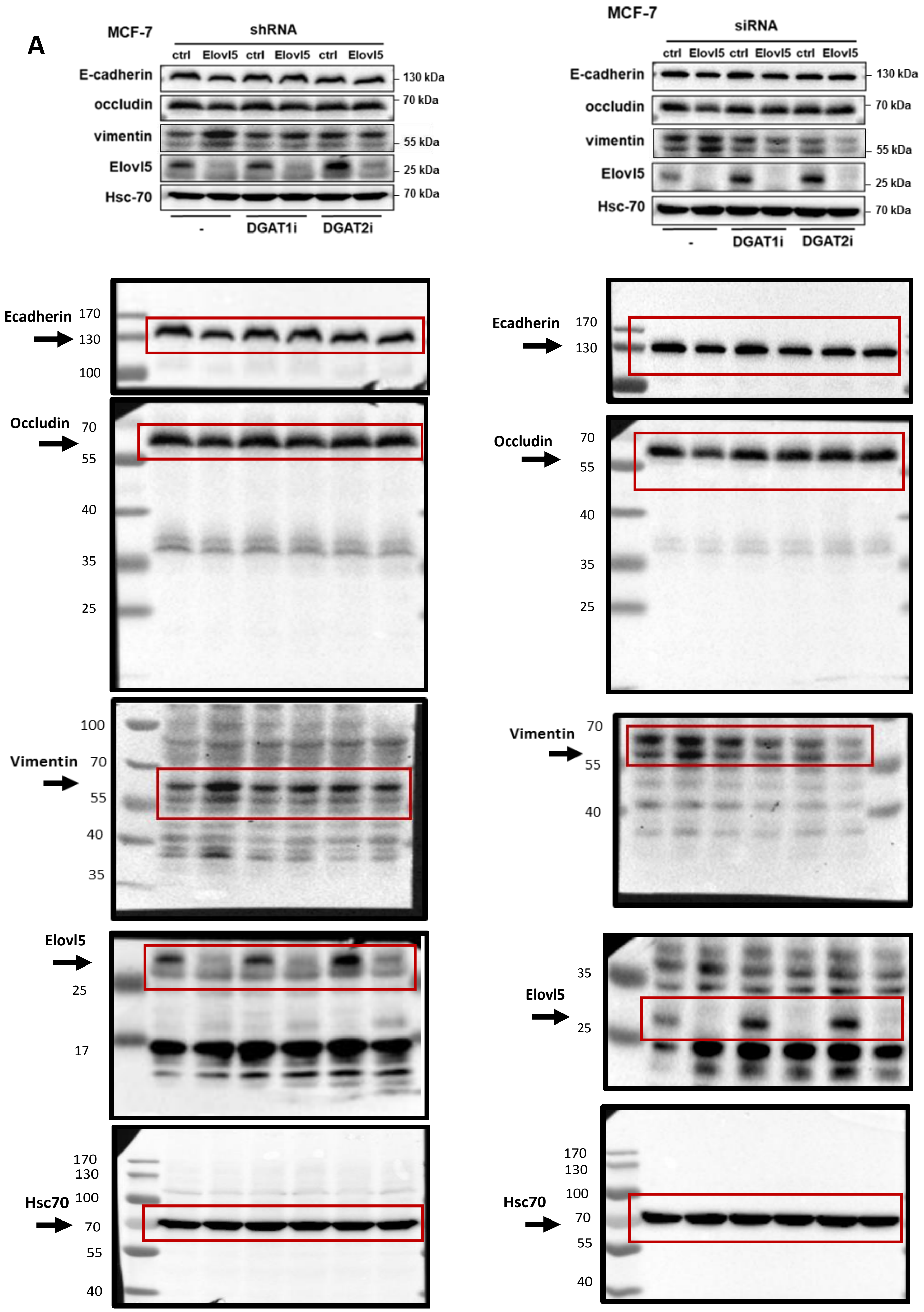
A
MCF-7
shRNA
ctrl Elovl5 ctrl Elovl5 ctrl Elovl5
E-cadherin
occludin
vimentin
Elovl5
Hsc-70
130 kDa
70 kDa
55 kDa
25 kDa
70 kDa
-
DGAT1i
DGAT2i
MCF-7
siRNA
ctrl Elovl5 ctrl Elovl5 ctrl Elovl5
Ecadherin
Occludin
Vimentin
Elovl5
Hsc70
170
130
100
70
55
40
35
25
17
